# Burden of bacterial antimicrobial resistance associated with modifiable human antibiotic consumption: An observational study

**DOI:** 10.1371/journal.pmed.1005140

**Published:** 2026-10-26

**Authors:** Benjamin J. Singer, Alec Gleason, Isabella Impalli, Mikkel D. Nagorsen, Chungman Chae, Lauren C. Granskog, Philip C. Lee, Ramanan Laxminarayan, Joseph A. Lewnard

**Affiliations:** 1 School of Public Health, University of California, Berkeley, California, United States of America; 2 One Health Trust, Bengaluru, India; 3 Computational Health Informatics Program, Boston Children’s Hospital, Boston, Massachusetts, United States of America; 4 School of Medicine, University of California, San Diego, California, United States of America; 5 Center for Computational Biology, College of Computing, Data Science & Society, University of California, Berkeley, California, United States of America; 6 School of Public and International Affairs, Princeton University, Princeton, New Jersey, United States of America; Boston University School of Public Health, UNITED STATES OF AMERICA

## Abstract

**Background:**

Human antibiotic use is widely believed to contribute to the evolution and maintenance of antimicrobial resistance (AMR) in bacterial pathogens. While antibiotic stewardship should thus discourage use in situations where AMR-related harms outweigh clinical benefit, evidence to inform this assessment of tradeoffs is lacking, most notably in low-resource settings where gaps in antibiotic access persist. Quantitative understanding of use-resistance relationships is thus necessary as countries navigate commitments from the 2024 UN General Assembly resolution to reduce AMR burden by 2030.

**Methods and findings:**

We used global surveillance data on AMR prevalence from 2000 to 2018 representing over 30 million bacterial isolates from 114 countries, along with antibiotic consumption estimates for each country and year, to estimate elasticity relationships between AMR and antibiotic exposure. Our analyses addressed 16 bacterial pathogens and antibiotic consumption across 7 drug classes, controlling for country-level health and development indicators via weighted regression models. Across pathogens, evidence that AMR is associated with population-level antibiotic exposure is strongest for *Acinetobacter* spp., *Streptococcus pneumoniae*, *Staphylococcus aureus*, *Pseudomonas aeruginosa,* and *Escherichia coli*. In a modeled counterfactual scenario, we estimate that 10% lower levels of antibiotic use, in comparison to current levels, across classes, would be associated with 38,000 (95% confidence interval: 36,000–41,000) fewer annual deaths attributable to AMR globally. Although substantial, this total represents only 0.50% of estimated bacterial disease mortality. In sub-Saharan Africa, where risk of death due to bacterial disease is greatest, the modeled avertible burden of AMR-attributable deaths comprises only 0.34% of bacterial disease mortality. Key limitations of the study include reliance on ecological relationships to infer modifiable burden, and under-representation of low-resource settings in surveillance data on antibiotic consumption and AMR.

**Conclusions:**

Our findings suggest near-term achievable reductions in AMR-attributable deaths may be modest relative to the persisting burden of bacterial disease mortality. These estimates should inform assessments of the potential benefits and harms of restrictive versus permissive antibiotic use paradigms in epidemiologically distinct settings.

## Author summary

**Why was this study done?:**

- Antimicrobial resistance (AMR) threatens our ability to treat bacterial diseases.
- Increased use of antibiotics is widely believed to contribute to higher levels of AMR.
- Quantitative estimates of the relationship between antibiotic use and AMR for different bacteria and antibiotics will help inform further research and public health decisions on how to tackle AMR and bacterial disease more broadly.

**What did the researchers do and find?:**

- We used several publicly available datasets on AMR prevalence in different bacterial species across the globe from 2000 to 2018, and paired these data with country-specific estimates of antibiotic use in different drug classes.
- We used a statistical model to estimate the relationship between changes in antibiotic use and changes in AMR prevalence in these data, finding evidence of a relationship between antibiotic use and AMR prevalence in several (but not all) types of bacteria and antibiotics.
- Our estimates imply that a global reduction in antibiotic use by 10% would be associated with 38,000 fewer annual deaths due to AMR, corresponding to 0.5% of all deaths due to bacterial disease.

**What do these findings mean?:**

- There is an observable relationship between AMR and antibiotic use across countries and over time—though our analysis does not reveal the mechanisms of this relationship or its consistency across times and places.
- While our findings imply fewer AMR-associated deaths may occur under a counterfactual scenario with lower antibiotic use, the estimated difference is modest relative to the total burden of bacterial disease.
- This understanding can help weigh the benefits and harms of expanding access to antibiotics.
- The study is limited by under-representation of low-resource settings in datasets monitoring antibiotic use and AMR profiles of bacterial pathogens.

## Abstract 3

In an observational study, Benjamin Singer and colleagues estimate the effects of changes in antibiotic use and prevalence of antimicrobial resistance, finding that a 10% decrease in global antibiotic use would reduce total bacterial infection deaths by only a very small percentage.

## Introduction

Antimicrobial resistance (AMR) in bacterial pathogens is an important cause of mortality globally, with 1.14 million excess deaths attributed to AMR (relative to those expected to occur if all infections were drug-susceptible) in 2021 [1]. Human antibiotic use is widely believed to contribute to the evolution and maintenance of AMR in bacterial pathogens [2,3]. Although ecological mechanisms underlying this relationship remain incompletely resolved [4], numerous examples document drug- or pathogen-specific associations between antibiotic consumption and AMR at varying spatial and temporal scales [5–11]. Concerns over antibiotic exposure as a driver of resistance have thus informed stewardship efforts—aimed at restricting antibiotic use in clinical situations where it may be unnecessary—within most national AMR action plans [12,13].

Although intended to align antibiotic use with clinical need, antibiotic stewardship interventions may raise challenges in situations where providers navigate uncertainty about causes of patients’ illness and the clinical value of treatment. For common syndromes precipitating most antibiotic consumption—such as acute respiratory and febrile illnesses—rapid diagnostic tools remain limited, and etiology may be difficult to ascertain from clinical signs and symptoms alone [14]. These challenges are compounded in low- and middle-income countries (LMICs), where healthcare systems may rely on informal facilities or medically unqualified personnel to distribute antibiotics [15], and where limitations in antibiotic access, quality, and availability [16–19] contribute to >3-fold higher age-standardized risk of bacterial disease mortality compared to that seen in high-income countries (HICs) [20]. Given these differences across epidemiologic contexts, it is also uncertain whether stewardship frameworks devised around benefits of antibiotic treatment and harms of AMR selection in HICs translate to LMICs [21–23]. For instance, findings that community-based mass antibiotic distribution reduces all-cause mortality among infants [24], young children [25,26], and pregnant mothers [27] in certain low-income settings suggests the burden of antibiotic-preventable deaths remains substantial.

Randomized studies assessing the burden of AMR preventable by changes in population-based antibiotic use are infeasible, and the mechanistic complexity of resistance selection restricts efforts to model downstream impacts of changes in use on AMR burden [4]. While recognition of ecological relationships between use and resistance has motivated efforts to address AMR burden through reduced antibiotic consumption [13], quantitative understanding of use-resistance relationships is needed to compare benefits and harms of restrictive or permissive antibiotic use strategies. To date, studies investigating associations of antibiotic consumption with AMR have typically focused on Europe [7,28–30] and the United States [10,31], where data availability is high. Significant associations between consumption and resistance have been observed variously for beta-lactams, macrolides, and fluoroquinolones in *Escherichia coli*, *Pseudomonas aeruginosa*, and *Streptococcus pneumoniae*, among other drug classes and pathogens—though in some studies confounders overwhelm these effects. There is little evidence of whether similar patterns hold globally and within LMICs, in particular, where AMR burden is greatest [32]. We sought to quantify the population-level association of antibiotic consumption over the period from 2000–2018 with resistance across bacterial species and drug classes, using AMR surveillance data and antibiotic use estimates across 114 countries. From the inferred relationships, we estimated the difference in AMR-attributable mortality expected under scenarios achieving lower community antibiotic consumption targets [33–35].

## Methods

### Data sources

We used publicly available data on the prevalence of resistance to 7 classes of antibiotics in bacterial pathogens previously identified as accounting for most resistant disease burden [1]. We abstracted data at country-year resolution on the numbers of resistant and tested bacterial isolates from four datasets: the WHO Global Antimicrobial Resistance and Use Surveillance System (GLASS) database [36], the CAESAR project [37], the ECDC AMR Surveillance atlas [38], and the ATLAS database [39]. The GLASS database aggregates data from a global effort to standardize AMR surveillance, including epidemiological, clinical, and population-level data from 110 countries. Included in GLASS is another WHO project, Central Asian and European Surveillance of Antimicrobial Resistance (CAESAR), which focuses on 14 countries in central Asia and Europe. The European Centre for Disease Prevention and Control (ECDC) AMR Surveillance atlas combines CAESAR data with data from the European Antimicrobial Resistance Surveillance Network, which uses compatible methodologies for data gathering and focuses on member states of the European Union (maximum of 28 countries over the study period). Finally, Antimicrobial Testing Leadership and Surveillance (ATLAS) is a project by Pfizer started in 2004, incorporating three separate surveillance programs (TEST, AWARE, and INFORM) covering 73 countries.

We excluded pathogen-antibiotic class combinations for which there was no recorded resistance; for which there were 10 or fewer independent observations (i.e., country-year-database combinations); for which fewer than 100 isolates were tested; and for sexually-transmitted infections (e.g., *Neisseria gonorrhoeae*), where population-level antibiotic consumption was expected to provide a poor proxy of antibiotic exposure in comparison to use within targeted risk groups [40]. These criteria excluded data for *Proteus* spp. and *Shigella* spp.; additionally, data on *Citrobacter* spp. were not available for the time period of 2000–2018. As some databases combined testing results for *Salmonella typhi*, *Salmonella paratyphi*, and non-typhoidal *Salmonella*, we aggregated data across these pathogens (where presented separately) and analyzed *Salmonella* spp. at a genus level. We used antibiotic consumption estimates by country and year from 2000 to 2018 from the GRAM dashboard for primary analyses [41]. We classified antibiotics according to anatomical therapeutic classification (ATC) letter codes (J01A–M), and conducted subgroup analyses using resistance data at higher-specificity drug class resolutions where available. These analyses distinguished resistance to glycyclines versus other tetracyclines; beta-lactam drugs including piperacillin-tazobactam, cephalosporins (by generation) as well as ceftazidime, carbapenems, monobactams; penicillins and oxacillin/methicillin (applicable for reporting of *S. aureus* resistance only); fluoroquinolones; and high-level aminoglycosides or amikacin.

We also conducted secondary analyses using unprocessed antibiotic sales data from 67 countries over the period 2008–2018 from the IQVIA MIDAS database, aggregated as DDDs per 1,000 person-days [42]. However, as both the measured distribution channels (e.g., retail, wholesale, or manufacturer-based) and data coverage differ across countries, these analyses were intended to provide conceptual validation of our primary findings rather than numerical estimates for head-to-head comparison.

### Regression

As a measure of the association between antibiotic pressure and AMR, we aimed to quantify the elasticity (ε) of AMR prevalence in each organism with respect to antibiotic consumption in each drug class. We defined this estimand as the ratio of the proportional change in AMR prevalence against a specific drug class, for a given pathogen, to the proportional change in antibiotic consumption for the same drug class, i.e., $\varepsilon =\frac{x}{y}\frac{\partial y}{\partial x}$, where y measures AMR prevalence (on a percentage scale) and x is the antibiotic consumption. We used regression to estimate elasticity coefficients relating consumption and resistance for each drug-pathogen pair. We fit a linear model to the natural log transforms of consumption and the percentage of isolates of each pathogen resistant to the same drug, weighting each observation proportionally to the total number of isolates tested for resistance. This approach is conceptually similar to use of count-based models with a log link function, where observations derived from greater numbers of isolates would contribute greater influence over the likelihood. To accommodate zero-valued observations, we applied a continuity correction of +1% to the percentage of isolates exhibiting resistance. Using the log-transform allowed us to estimate the elasticity directly from the gradient of the linear model, since $\frac{\partial \text{log}(y+\text{1})}{\partial \text{log}(x)}=\frac{x}{y+\text{1}}\frac{\partial y}{\partial x}\approx \;\varepsilon$, by the chain rule, when *y* ≫ 1%; our estimates of elasticity are thus most valid when AMR prevalence is high relative to 1%. We compared to results obtained with a continuity correction of +0.01% as a sensitivity analysis. These continuity corrections allow unrealistic values of 101% or 100.01% for observations with complete resistance. As a sensitivity analysis, we also estimated elasticity coefficients using binomial and beta-binomial models (accommodating overdispersion) defining counts of resistant and total isolates as the outcome to avoid the need for continuity correction; however, as these models use a logit link function, coefficient estimates relate antibiotic use consumption to the log odds of resistance rather than the log prevalence.

For all models, we estimated confidence intervals using country-clustered bootstrapping, resampling countries for inclusion in each of 10,000 iterations; all reported confidence intervals are based on these bootstraps. We present point estimates and 95% confidence intervals to illustrate effect size estimates, and apply the Benjamini–Hochberg correction to adjust *p*-values for multiple testing when assessing statistical significance of estimates for each pathogen-drug pair. For primary analyses, we paired consumption and resistance data from the same year, based on evidence from prior studies that have detected selection for AMR on short (e.g., seasonal) scales [43–45]. As a sensitivity analysis addressing longer-term trends, we also estimated elasticity of AMR with respect to consumption one, three, or five years prior.

To adjust for potential confounding related to differences across countries or over time in population characteristics (S5 Fig), socioeconomic factors, health systems infrastructure, and related issues, we extracted data on 328 health and economic variables by country and year from the World Bank Health, Nutrition, and Population Statistics (WBHNP) database [46] for the full study period. Because including all measured variables in our unregularized models could lead to overfitting, we performed principal components analyses of these data, extracting the first three linear combinations of all variables for inclusion in our regression analyses (beyond which each additional principal component accounted for a small proportion of variance; S5, S6, and S7 Tables; S6 Fig). We used the missMDA package [47] to derive principal components via a joint procedure implementing multiple imputation of missing values in the WBHNP data, and we excluded variables for which >30% of country-year observations were missing (*n* = 155 variables). Broadly summarizing these derived principal components based on the variables with greatest absolute loadings, the first relates to early childhood vaccinations and mortality, the second to population age distribution, and the third to mortality from accidents, suicide, and non-communicable illness. Sensitivity analyses tested inclusion of the first 10 linear combinations, and restriction to the first due to potential multicollinearity among predictors included in regression models (as reflected by variance inflation factors). Models additionally controlled for countries’ inflation-adjusted annual gross domestic product per capita and calendar year. Because health, nutrition, and population covariates did not provide a strong basis for imputing resistance prevalence, we restricted analyses to country-years with available AMR surveillance data for each drug and pathogen. This resulted in over-representation of HICs in our dataset (S2 Fig); to understand potential selection bias, we repeated analyses within HIC and LMIC strata.

To generate pooled estimates of elasticity at the level of antibiotic classes, we fit linear models using the *lme4* R package, assuming for parsimony uncorrelated random intercepts and slopes by pathogen species. Similarly, to generate pooled estimates at the level of pathogens across drug classes, we allowed for random intercepts and slopes by antibiotic class. To enable comparisons across drug classes with differing levels of use, we scaled consumption data for each antibiotic class by mean global consumption of drugs within the same class in 2018, in addition to performing a sensitivity analysis scaling by same-class consumption in 2000.

We used the type II ANOVA method to determine the proportion of variation explained by each covariate included in the regression analysis [48,49]. We also investigated influential observations with Cook’s distance above 1, and compared our results to estimates obtained in analyses excluding these data points.

### Permutation testing for specificity of consumption and AMR relationships

If the relationship between antibiotic consumption and AMR prevalence is not specific by drug class, then it may reflect an artefactual relationship of AMR with other features of health systems predicting levels of antibiotic use. To examine this possibility, we repeated analyses with random permutations of drug class within the antibiotic consumption data. We created a set of permutations of antibiotic consumption data by drug class comprising each possible incorrect assignment of drug class within the dataset. We re-calculated elasticities using the same regression analysis framework described using these permuted data, generating a sample of elasticities calculated with antibiotic consumption measured in antibiotic classes other than those where resistance was measured. We then used the Wilcoxon signed-rank test [50]—a non-parametric paired difference test—to determine whether the distributions of elasticity coefficients estimated with the true data differed from the elasticities calculated using the permuted data. This approach tests for, but cannot rule out, unmeasured confounding variables in the relationship between antibiotic consumption and AMR.

### Counterfactual burden estimation

Reductions of 10% or more in antibiotic consumption are estimated to be achievable under recently adopted commitments to address AMR through expanded access to a suite of health interventions encompassing vaccination and water, sanitation, and hygiene (WASH) services, which are anticipated to reduce the incidence of acute illnesses responsible for substantial volumes of antibiotic use [33–35]. To assess implications for resistant disease burden, we used our elasticity coefficient estimates to quantify AMR-attributable mortality expected under a counterfactual scenario involving 10% lower levels of consumption of each antibiotic class. While reductions in antibiotic use associated with differing interventions may vary by drug and setting, the 10% benchmark represents a realistic target for reducing all-cause antibiotic use through interventions aimed at infection prevention or enhanced stewardship [33,51–54]. Recent targets for reductions aimed at aligning countries’ antibiotic use with bacterial disease burden currently exceed 10% for most countries and antibiotics [55].

While the elasticities that we estimated in the first part of our analysis were associative in nature, ecological relationships between use and resistance prevalence provide a rational basis for projecting counterfactual disease burden under scenarios involving modest changes in antibiotic use, absent other forms of evidence. To quantify potential differences in mortality implied by this scenario, we used prior Global Burden of Disease estimates of the number of deaths attributable to bacterial disease and AMR by region, year, pathogen and antibiotic class [1]. These estimates are globally comprehensive and derived from a consistent methodology, even when data are sparse, and were reported with uncertainty intervals. We used our elasticity estimates to evaluate a counterfactual scenario in which some treatment-resistant infections were instead treatable (this hypothetical derives from the definition of AMR-attributable deaths). We assumed reductions in AMR were evenly distributed between patients at risk of death and not at risk of death and defined the proportion of AMR-attributable deaths avoided with x% lower antibiotic use as 1−exp(ε×ln[1−x/100]), where ε represented the pathogen- and class-specific elasticity coefficient. For pathogens not included in our regression analyses, we sampled from the distribution of pathogen-specific random effects for the corresponding antibiotic class to generate estimates for ε. We propagated uncertainty by sampling from Gamma distributions fitted to reported means and 95% uncertainty intervals around estimates of AMR-attributable deaths, stratified by setting, pathogen, and drug, and multiplied draws from the resulting distributions by independent draws from ε estimates. All reported uncertainty intervals for counterfactual burden estimates thus accommodate uncertainty in elasticity coefficients and in underlying AMR-attributable mortality. We summed estimates of total avertible burden across settings, drugs, and pathogens.

In addition to overall analyses by drug class, we estimated upper- and lower-bound effect sizes for counterfactual burden under scenarios where numerical estimates of elasticity informed the targeting of each drug class for reductions in use. For upper bounds, we assumed that the x% reduction in antibiotic use in each setting was concentrated in the drug class with highest overall ε, from the model assigning random slopes by pathogen. We assumed a maximum reduction of 90% for any class. Provided a drug class made up less than 0.9×x% of total antibiotic use for a given setting, we assigned the remaining reduction to the drug class with the second highest ε, and so on. For lower bounds, we did the same but starting with the drug class with the lowest ε. We also explored scenarios in which elasticity was 2-fold higher or lower than our estimates, i.e., with elasticity equal to 2ε or ε/2, which could arise under violation of assumptions such as non-reversibility of resistance, or presence of feedback loops that accelerated or hampered pathogen responses to changes in antibiotic pressure.

We estimated the relationship between counterfactual burden and GDP per capita, and the relationship between counterfactual burden and current total antibiotic consumption, via random-effects metaregression models defining the estimated difference between current and counterfactual burdens (aggregated across all pathogens and drug classes) as an outcome variable. Hypothesizing non-linear effects, we tested linear and quadratic formulations of the predictor variables and selected the model that minimized the Bayesian Information Criterion (BIC).

### Within-hospital antibiotic use estimates

While the greatest volume of antibiotic consumption occurs in outpatient and community settings [7], selective pressure resulting from in-hospital antibiotic use may be of particular concern for pathogens transmitted via nosocomial pathways [56]. To supplement our analyses relating AMR prevalence to population-wide antibiotic use with observations limited to hospital settings, we reviewed published studies on the population-level association of resistance in specific pathogen species with consumption of antibiotics of corresponding drug classes in hospitalized patients. To identify articles potentially providing eligible data, we reviewed all articles included in a previous (2014) systematic review and meta-analysis on the subject [2], and searched for new articles published from 2013 to 2023 via PubMed using the string *antibiotic* AND (*susceptibility* OR *nonsusceptibility* OR *non-susceptibility* OR *resistance* OR *sensitivity* OR *nonsensitivity*) AND (*consumption* OR *prescription* OR *defined daily dose*) AND (*association* OR *prevalence* OR *correlation*) NOT (*poultry* OR *livestock* OR *animal* OR *agriculture* OR *mouse* OR *swine* OR *cattle*) NOT (*meta-analysis* OR *opinion* OR *review*). We supplemented this search with all articles identified as citing the previous systematic review on this subject [2] identified via Google Scholar and published through the end of 2023. We included data from all English-language studies that measured within-hospital antibiotic use in DDD/1,000 patient-days, or a unit that could be converted to DDD/1,000 patient-days. Our analyses of the association of antimicrobial consumption with AMR prevalence in hospital settings used the same log-log regression framework as our primary analyses, and included the same variables for adjustment. As data on underlying counts of isolates tested were not reported in all studies, regression analyses for within-hospital patterns did not include weights by isolate counts. This analysis may therefore be sensitive to variability arising in small studies, as well as heterogeneities in study design.

### Reporting and transparency

This study was reported in accordance with RECORD and GATHER guidelines; the accompanying checklists are included with the supporting information (S1 and S2 Checklists). Data and code to reproduce the analyses are available from GitHub https://github.com/benjaminjohnsinger/ModifiableAMR and archived in Zenodo with DOI https://doi.org/10.5281/zenodo.21925465. Code was produced using R version 4.5.1, with packages lme4 version 1.1.37 and MuMIn version 1.48.19. LLMs were used to produce some of the code for this project, with appropriate checks and testing by human programmers; they were not used to define the study objectives, to produce figures, or in writing the manuscript; LLMs used included Gemini 2.0–3.6 Thinking, Gemini 2.0–3.1 Pro, and models accessed through GitHub Copilot in VSCode (listed individually in S4 Text). As the study used pre-aggregated data from publicly available sources, without the possibility of disaggregation to individual-level resolution or linkage to individual identifiers, the project did not require review under the Common Rule (45 CFR 46). The study proceeded without a prespecified analysis plan.

## Results

### Data characteristics

The best-represented pathogens in our combined dataset were *Escherichia coli* (10,285 country-year observations) and *Klebsiella pneumoniae* (9,718 observations), while non-penicillin beta-lactams comprised the drug class with the most extensive susceptibility characterization (20,167 observations); we tabulate data coverage for each pathogen and antibiotic class in S1 Table. In total, data represented 27,352,898 bacterial isolates from high-income countries (HICs) and 2,701,141 isolates from LMICs. The most-represented region was western Europe, with 15,746,612 isolates, and the least-represented region was central Asia, with 1,461 isolates. There were 69,998 isolates from sub-Saharan Africa, and 263,277 from South Asia. The total number of isolates per year increased by about 67,000 each year from 2000 (19,384 isolates) to 2016 (1,091,921 isolates), then was much higher in 2017 (9,791,723 isolates) and 2018 (10,658,932 isolates). Only countries in western and central Europe were represented before 2004.

### Relationship between antibiotic consumption and resistance

Quinolones demonstrated the greatest elasticity in AMR prevalence with respect to antibiotic consumption, with *ε* = 0.53 (95% confidence interval: 0.33 to 0.68) indicating that a 1.1-fold increase in quinolone DDDs corresponded to a 1.053-fold (1.033 to 1.068) increase in the proportion of isolates of the studied pathogens exhibiting quinolone resistance, allowing for random effects by bacterial species (Fig 1). Other antibiotic classes for which we observed high elasticity in AMR with respect to consumption included penicillins (*ε* = 0.29 [0.06 to 0.69]) and non-penicillin beta-lactams (*ε* = 0.23 [0.02 to 0.42]). Elasticity point estimates were larger than 0.2 for macrolides (*ε* = 0.43 [–0.02 to 0.96]) and aminoglycosides (*ε* = 0.22 [–0.01 to 0.40]) although estimates showed wider uncertainty. For the remaining drug classes, population-level increases in consumption did not correspond to large increases in resistance prevalence. Elasticity coefficients were 0.09 (–0.19 to 0.31) for sulfonamides and trimethoprim, and 0.02 (–0.07 to 0.15) for tetracyclines.

**Fig 1:**
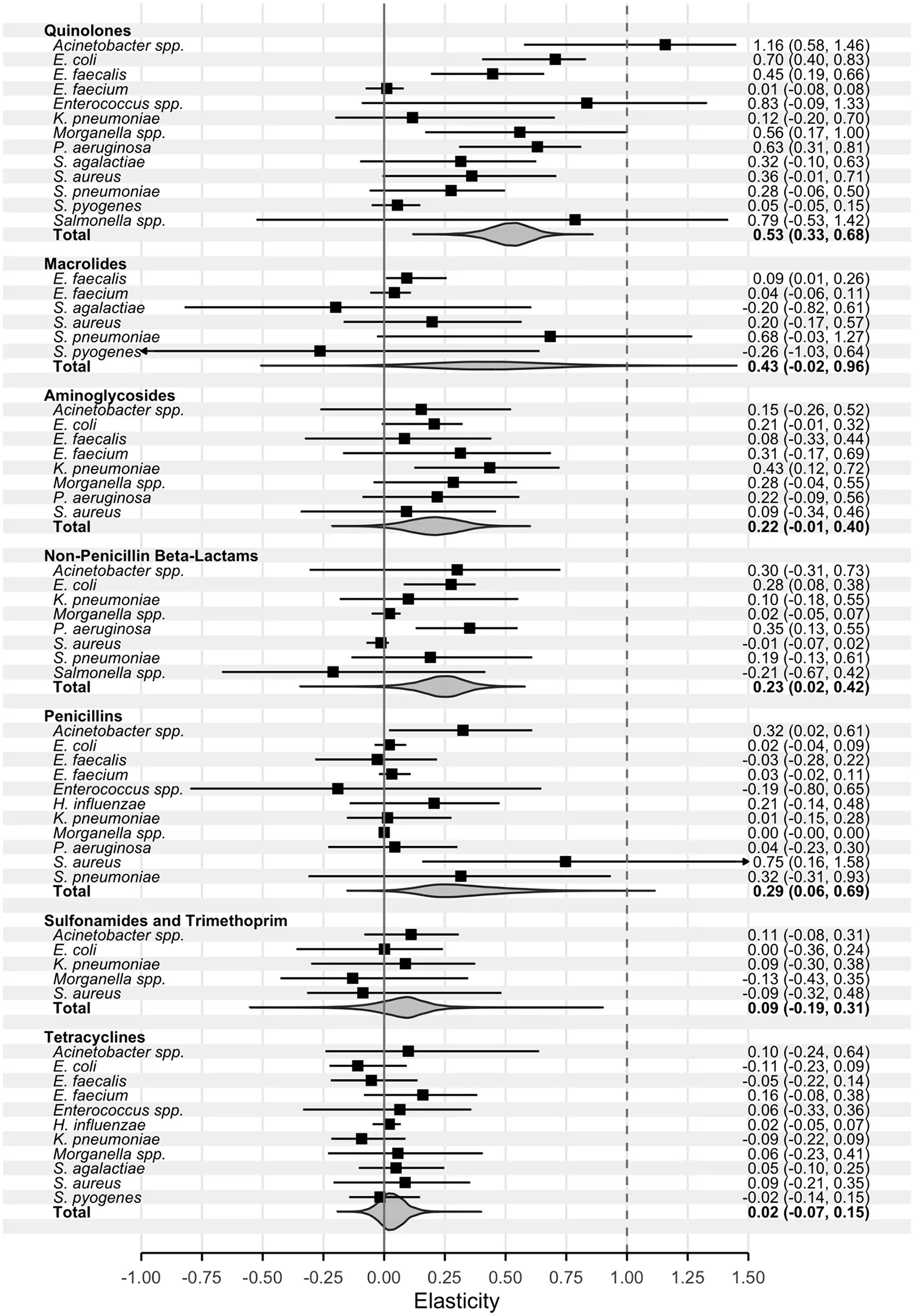
Pathogen- and class-specific elasticity. We illustrate estimated elasticity coefficients (with 95% confidence intervals) of AMR (antimicrobial resistance) prevalence with respect to population-wide consumption of antibiotics. The elasticity is the proportional increase in resistance expected from corresponding proportional unit change in consumption. We plot estimates for specific combinations of pathogen and antibiotic class, as well as violin plots of the overall (“Total”) estimates for each antibiotic class, treating pathogens as a source of random effects. We present estimates disaggregated by country income stratum (S10 and S11 Figs) as well as estimates categorizing resistance profiles at subclass-level resolution (S12 Fig) in the supporting information.

Disaggregated analyses identified differences in estimates across drugs within certain classes. Among beta-lactams, we estimated high elasticity for carbapenems (*ε* = 0.30 [0.09 to 0.45]) in comparison to piperacillin-tazobactam (*ε =* 0.12 [–0.02 to 0.31]), monobactams (*ε* = 0.07 [–0.08 to 0.23]), and cephalosporins (*ε* point estimates spanning 0.02–0.10 for third through fifth generation cephalosporins and ceftazidime; S12 Fig). Consistent with our high class-level estimates for aminoglycosides and quinolones, we estimated *ε =* 0.19 (0.01 to 0.35) for amikacin and 0.54 (0.33 to 0.68) for fluoroquinolones.

Across the antibiotic classes studied, *Acinetobacter* spp. exhibited the greatest elasticity (*ε* = 0.43 [0.19 to 0.56], overall, allowing random effects for antibiotic class; Fig 2). Point estimates for elasticity coefficients were above zero for all pathogens; other pathogens showing prominent elasticity across drug classes included *S. pneumoniae, S. aureus, P. aeruginosa, E. coli, K, pneumoniae,* and *Morganella spp.* The elasticities estimated for each pathogen were robust to changes in the choice of scaling for the antibiotic consumption variable, with any changes to point estimates for *ε* remaining within a margin below ±0.01 (S16 Table).

**Fig 2:**
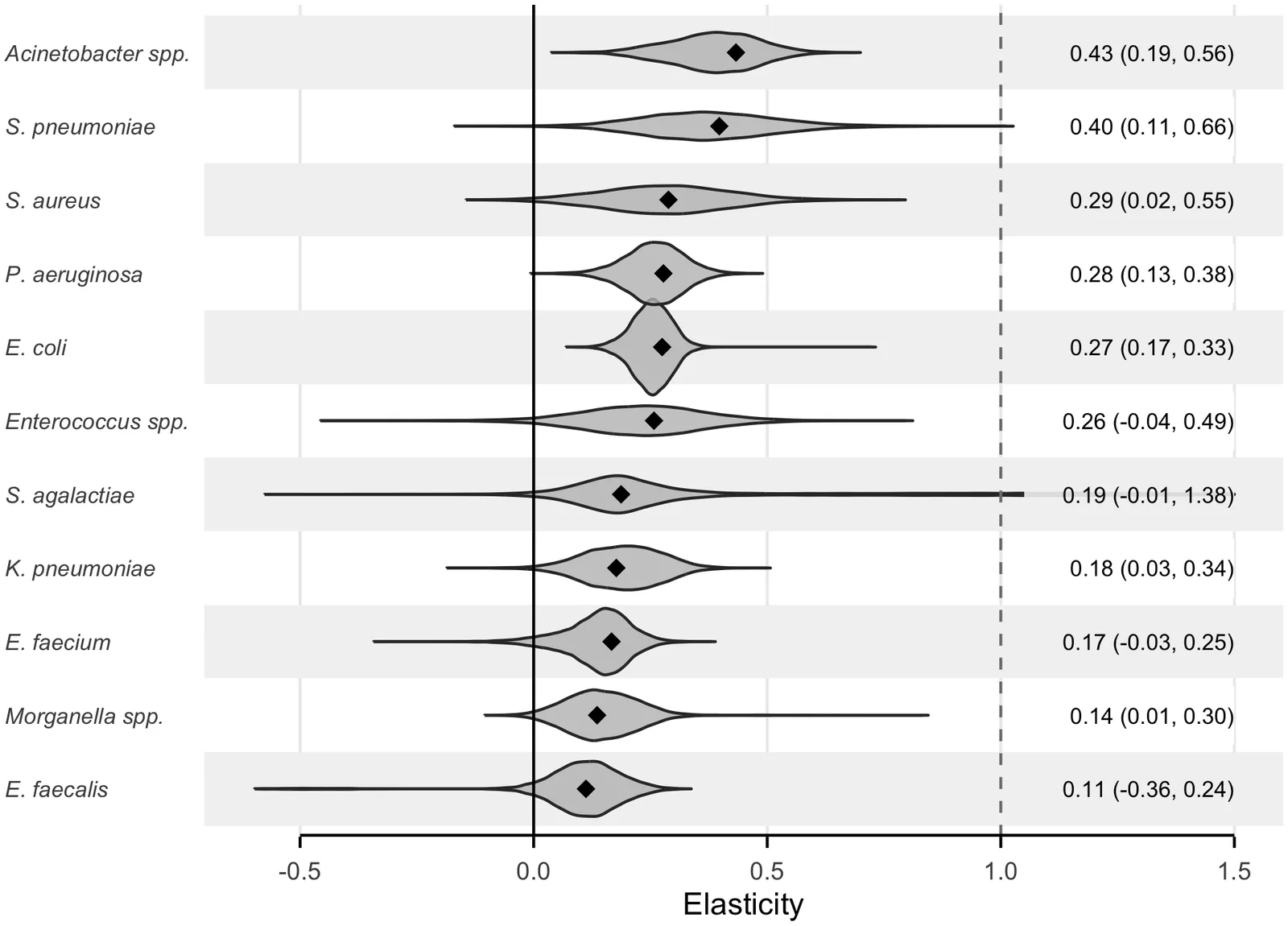
Elasticity by pathogen. Violin plots illustrate bootstrapped distributions conveying estimated elasticity of AMR (antimicrobial resistance) prevalence with respect to consumption of antibiotics, estimated for each pathogen in the data set, treating antibiotic class as a source of random effects. Pathogens for which the random effects model is singular are excluded, i.e., *H. influenzae* and *S. pyogenes*. Reported values (right column) indicate estimates with 95% confidence intervals.

Overall, our estimates indicate that elasticity varies more by drug class than by pathogen, as demonstrated by the standard deviations in estimated elasticity coefficients (sample average standard deviations: 0.18 [0.14 to 0.22] and 0.10 [0.06 to 0.15], respectively; Fig 1). Applying the Benjamini–Hochberg algorithm for significant relationships under multiple testing across all pathogen-drug pairs revealed four significant relationships (at a corrected *p* < 0.05 false-discovery threshold) between antibiotic consumption and AMR, all for consumption of quinolones with AMR in *Acinetobacter* spp., *E. coli*, *E. faecalis*, and *P. aeruginosa*.

The observed elasticities were similar in analyses including additional economic and healthcare variables, using different scaling of consumption, or incorporating a lag between antibiotic consumption and AMR observations (S16 Table). We also obtained similar results in analyses adjusting for only the first principal component of the WBHNP data based on potential multicollinearity among predictors (S13, S14, and S15 Tables; S15 Fig). In an analysis using a smaller continuity correction (+0.01%), some elasticities were much higher—for instance, an elasticity of 2.23 (0.57 to 2.91) was observed for quinolones in *S. agalactiae*, compared to *ε* = 0.32 (–0.10 to 0.63) in the main analysis. Overall, elasticities for *S. agalactiae*, *Enterococcus* spp*.*, and *S. pneumoniae* were higher in this analysis, revealing dependence of primary findings on observations from settings with low AMR prevalence.

We identified 31 influential observations across all analyses (Cook’s distance >1; S12 Table). Excluding these observations weakened the elasticity estimate for resistance to non-penicillin beta lactams in *E. coli* (*ε* = 0.01 [–0.31 to 0.43]) but strengthened estimates for associations within the same class with *Acinetobacter* spp. (*ε* = 0.42 [0.01 to 0.81]) and for quinolone resistance in *K. pneumoniae* (*ε* = 0.55 [0.28 to 0.76]; S16 Table; S14 Fig).

Sensitivity analyses fitting binomial models to count data from isolates yielded 95% confidence intervals around log-odds regression coefficients that excluded 0 more often than the elasticity estimates from our primary analyses, although dispersion coefficients exceeded 1 for all drug-pathogen pairs, supporting the use of beta-binomial models for count-based analyses (S17 Table; S15 Fig). In comparison to our primary analyses, estimates from beta-binomial models provided stronger evidence of use-resistance relationships in penicillins for *Morganella* spp., in other beta lactams for *S. pneumoniae*, in aminoglycosides for *P. aeruginosa*, and in quinolones for *K. pneumoniae*, *S. aureus*, and *S. pneumoniae*, although weaker evidence of relationships in non-pencillin beta-lactams for *E. coli* and *P. aeruginosa*. Drug classes associated with relatively larger or smaller coefficient estimates, or no clear evidence of use-resistance relationships, were the same for analyses using beta-binomial models as for our primary analyses.

For quinolones, non-penicillin beta-lactams, and aminoglycosides, elasticity point estimates differed within a ±0.06 margin between HICs and LMICs (S10 and S11 Figs). However, positive elasticity coefficients for macrolides and penicillins, as estimated in the primary analyses, were apparent only in analyses restricted to HICs (*ε* = 0.53 [0.11 to 1.06] and 0.19 [–0.03 to 0.62], respectively, in HICs; *ε* = –0.25 [–0.83 to 0.81] and –0.02 [–0.12 to 0.14], respectively, LMICs).

Repeating our analyses using unprocessed sales data from the IQVIA MIDAS database to quantify class-specific consumption (DDDs per 1,000 person-days) identified positive elasticity coefficients for 10 of 62 pathogen-drug class combinations (S16 Fig). Consistent with findings of our primary analyses, quinolones and non-penicillin beta-lactams were the drug classes associated with the greatest elasticity in *E. coli* (*ε* = 0.19 [0.00 to 0.28] and *ε* = 0.19 [0.05 to 0.25], respectively), whereas penicillins were associated with the greatest elasticity in *S. aureus* (*ε* = 0.36 [0.16 to 0.58]).

### Specificity of antibiotic use as a predictor of resistance prevalence

Permutation tests yielded significantly lower estimates of elasticity coefficients according to the Wilcoxon signed rank test statistic at two-sided *p* < 0.05 for 26 of the 50 pathogen-drug pairs for which we estimated positive elasticity coefficients (S2 Table), an outcome expected with probability 5.5 × 10^–21^ under the null hypothesis that use-resistance relationships lack specificity by drug class. This total included all 11 of the pathogen-drug pairs for which 95% confidence intervals around our original estimates excluded 0%.

### Relationship between antibiotic consumption in hospitals and resistance

We included data from 14 relevant studies, of which 12 analyzed resistance to non-penicillin beta-lactams, 8 analyzed resistance to penicillins, 7 analyzed resistance to quinolones, and 6 analyzed resistance to aminoglycosides (S3 Table). Contrary to our findings from analyses considering antibiotic use across all settings, elasticity estimates based on in-hospital antibiotic use were generally close to zero (S17 Fig). Class-specific elasticity coefficients were −0.11 (−0.25 to 0.47) and 0.16 (−0.26 to 0.42) for penicillins and non-penicillin beta-lactams, respectively.

### Counterfactual burden of deaths due to resistance

The estimated difference in AMR-attributable deaths associated with 10% lower consumption of each drug class, within each setting, totaled 38,000 (95% uncertainty interval: 36,000 to 41,000) per year (Fig 3). Here, 8.25% of the estimated difference in deaths relied on extrapolation using the antibiotic class-level random effects. In sensitivity analyses modifying the elasticity coefficients—including using alternative covariates, lagged consumption data, and lower continuity correction values—the estimated difference varied from 37,000 to 54,000 fewer deaths. An upper-bound estimate where reductions antibiotic use within each setting were concentrated in drugs with the highest elasticity yielded a difference of 110,000 (100,000 to 120,000) deaths. The lower bound estimate concentrating reductions in drugs with the lowest elasticity was a difference of 7,400 (320 to 13,000) deaths. In the scenario where elasticity is half or double our estimates, we estimated differences of 20,000 (18,000 to 21,000) or 74,000 (69,000 to 79,000) deaths.

**Fig 3:**
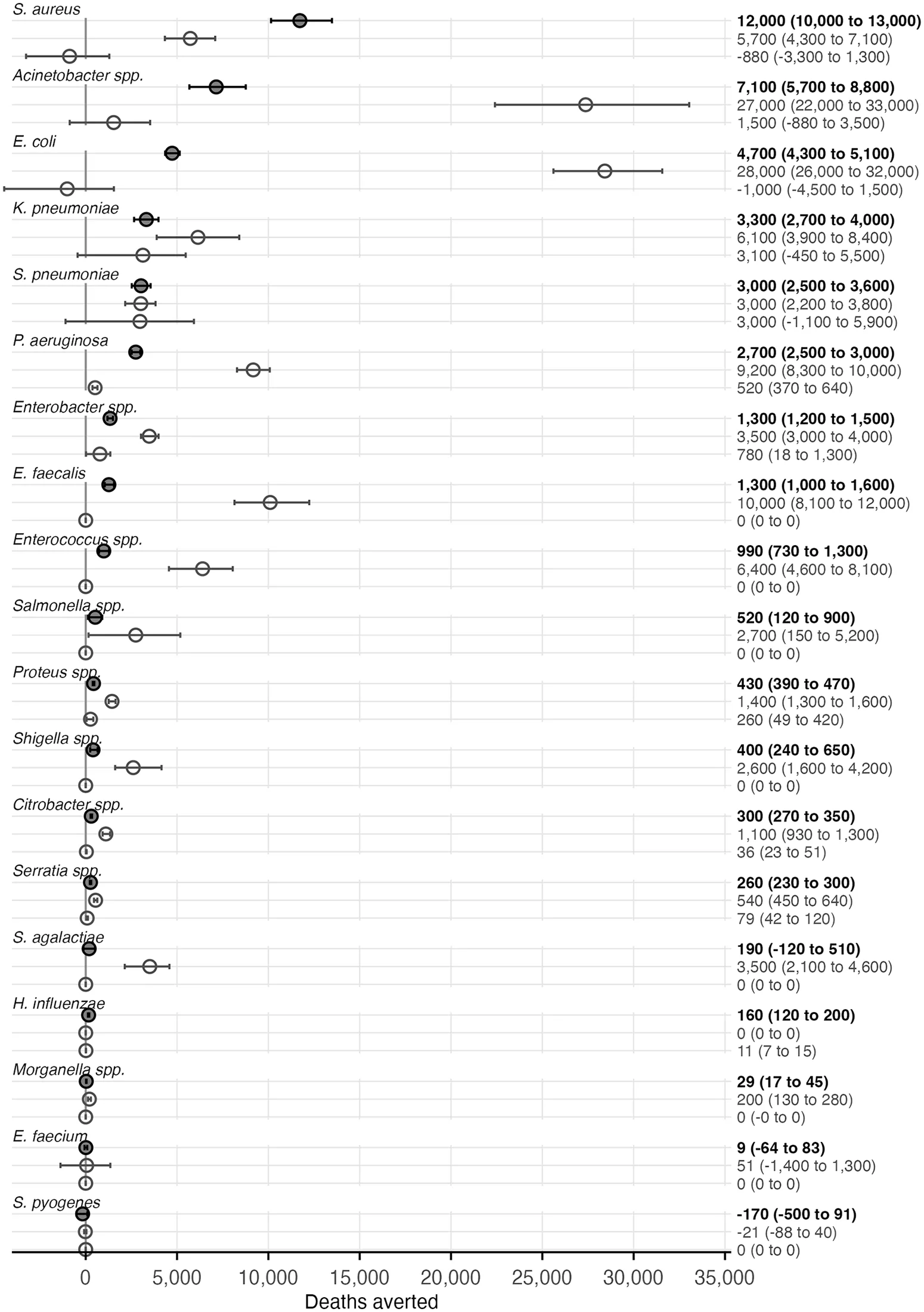
Difference in burden of AMR-attributable mortality. We illustrate estimates (with 95% confidence intervals) for differences in global annual AMR (antimicrobial resistance)-attributable deaths under a scenario with 10% lower antibiotic use, stratified by pathogen, and under even, high-elasticity, and low-elasticity targeting scenarios from top to bottom. Estimates project differences in AMR prevalence within each pathogen expected with 10% lower antibiotic use based on estimated elasticity in the pathogen and antibiotic class-specific relationship between AMR and consumption (Fig 1). We present estimates disaggregated by pathogen and region and by drug and region in the supporting information (S18 and S19 Figs).

Across regions, we estimated the greatest differences in counterfactual AMR-attributable mortality in South Asia and Western sub-Saharan Africa. In South Asia, we estimated 0.65 (0.57 to 0.75) fewer deaths per 100,000 person-years and 12,000 (10,000 to 14,000) fewer deaths annually, in total (S19 Fig); in Western sub-Saharan Africa, we estimated 0.70 (0.61 to 0.79) fewer deaths per 100,000 person-years and 3,100 (2,700 to 3,500) fewer deaths annually, in total. Stratifying by pathogen, *S. aureus* accounted for the greatest difference in deaths under a scenario with lower antibiotic use (12,000 [10,000 to 13,000]), while quinolone resistance accounted for the greatest number when stratifying by drug class (16,000 [15,000 to 17,000]; S18 Fig).

Although substantial, the estimated difference in AMR-attributable deaths accounts for only 0.50% (0.47%–0.53%) of estimated deaths due to bacterial diseases, globally [20]. This proportion varied by region, with the highest proportion in South Asia (0.64% [0.56%–0.74%]; Fig 4A and 4B), and correlated non-linearly with per-capita GDP and levels of antibiotic consumption across regions (BICs for linear and quadratic specifications, respectively, were −216 and −218 for GDP, and −171 and −175 for consumption). Measured as a proportion of bacterial disease burden, the estimated difference was greatest in settings with per-capita GDP of 29,400 US dollars at purchasing power parity (a level at which countries were considered high-income), and in settings with antibiotic consumption totaling 12.6 DDD per 1,000 person-days, corresponding roughly to levels of consumption in the Eastern Europe (12.5), Southern Latin America (12.9), South Asia (13.5), and North Africa and the Middle East (13.8) regions. Estimates of the difference in mortality rates per 100,000 persons under counterfactual scenarios declined across settings with increasing GDP per capita and antibiotic use, consistent with the lower burden of bacterial disease mortality in such settings (Fig 4C and 4D).

**Fig 4:**
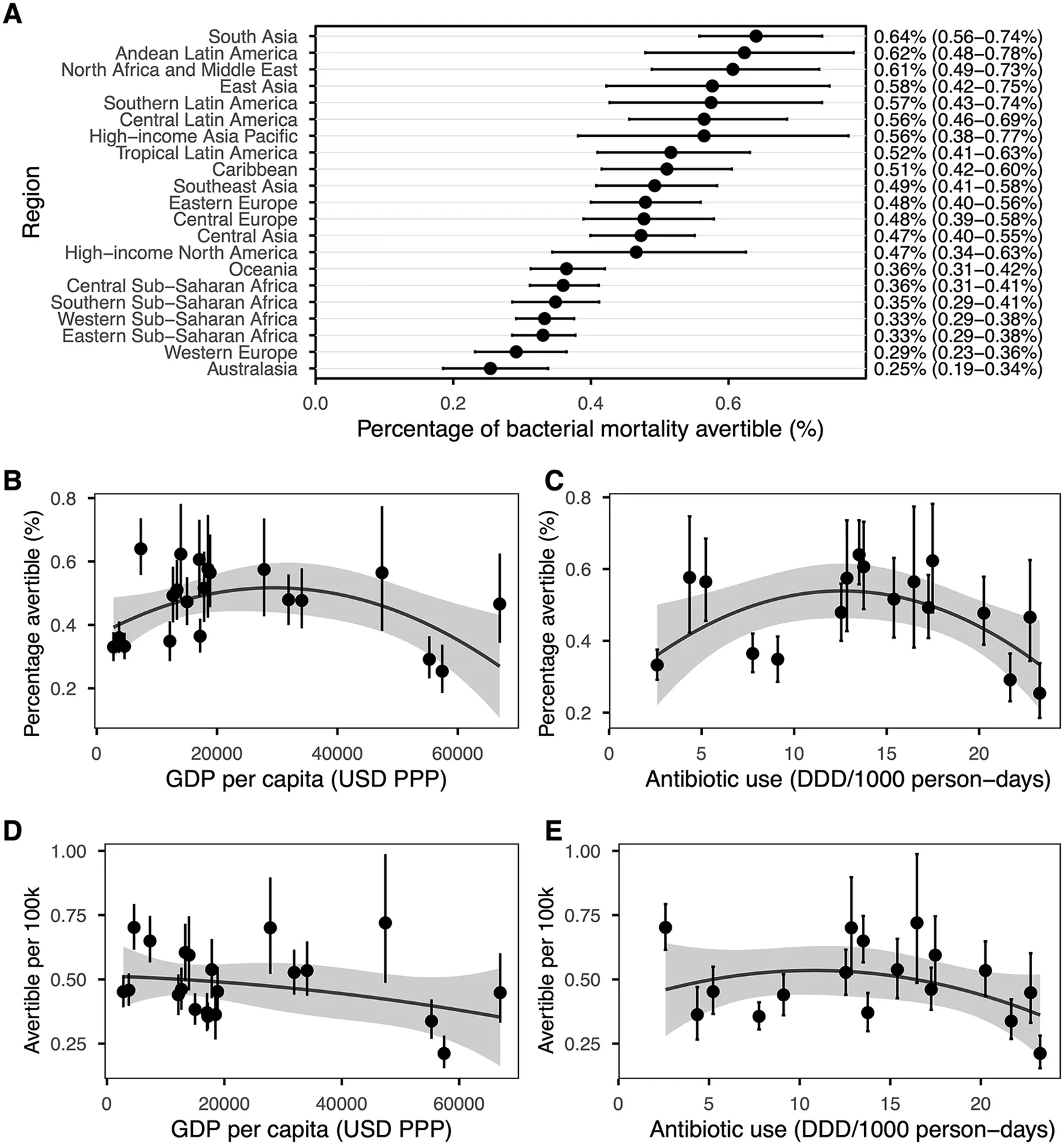
Difference in burden as a percentage of bacterial disease mortality. **(A)** Estimates (with 95% confidence intervals) of the difference between current and counterfactual AMR (antimicrobial resistance)-attributable mortality with 10% lower antibiotic use, measured as a proportion of current bacterial disease mortality and stratified by region. **(B)** Relationship of this difference in mortality, measured as a proportion of total bacterial mortality, with GDP per capita by region. **(C)** Relationship of this difference in mortality, measured as a proportion of total bacterial mortality, with antibiotic consumption (in DDD, defined daily dose) by region. **(D)** Relationship of the difference in AMR-attributable mortality per 100,000 persons with GDP (gross domestic product) per capita by region. **(E)** Relationship of difference in AMR-attributable mortality per 100,000 persons with antibiotic consumption by region. In panels B–E, shaded areas delineate 95% confidence intervals around mean estimates (lines) for the continuous relationship between the predictor (horizontal axis) and the outcome variable (vertical axis). Vertical lines illustrate 95% confidence intervals around country-specific measures plotted via points.

## Discussion

Our analysis demonstrates elasticity in AMR prevalence associated with antibiotic consumption across a wide range of bacterial pathogens and antibiotic classes. Drug classes for which differences in use were associated with the greatest differences in AMR prevalence within bacterial pathogens included quinolones (and particularly fluoroquinolones), aminoglycosides (including amikacin), macrolides, penicillins, and other beta-lactam antibiotics (notably including carbapenems piperacillin-tazobactam). For other classes, associations were more often specific to individual pathogens or settings. Elasticity in AMR prevalence appeared drug-specific on the basis of our permutation analyses. These findings broadly support the hypothesis that resistance profiles of bacterial pathogens accounting for most AMR-attributable mortality may be modifiable by changes in antibiotic consumption. It is important to note that our estimates are ecological in nature and do not provide decisive causal inferences; instead, we use observed differences in AMR prevalence across countries, adjusted for confounding variables related to health and socioeconomic conditions, to project differences under counterfactual scenarios involving lower antibiotic consumption. Nonetheless, the level of elasticity we estimate implies that differences in AMR-attributable mortality achievable through realistic reductions in consumption may be modest relative to the persisting global burden of bacterial diseases, and in comparison to reductions in disease burden achievable through improved treatment and prevention measures. Impacts of interventions favoring permissive or restrictive antibiotic use on AMR-preventable mortality merit consideration in the context of their overall impact on the burden of bacterial diseases, particularly in low-access contexts where this burden is greatest.

As our analyses address population-level antibiotic consumption as an exposure, the elasticity we measure may reflect selection pressure driven by the treatment of non-severe infections in outpatient or community settings, which account for the vast majority of consumption measured in surveys and drug sales datasets [41,42,57,58]. Selection for resistance occurs not only during treatment of targeted pathogens but also among “bystander” organisms colonizing the host [59]. Such exposures have been estimated to account for a vast majority of antibiotic exposure occurring in bacteria such as *S. pneumoniae*, *S. aureus*, *K. pneumoniae*, *Streptococcus agalactiae*, *P. aeruginosa*, *E. coli*, and *Shigella* spp. [60,61], each of which were associated with positive elasticity coefficients for multiple drug classes in our analyses. Absence of similar associations in other pathogens may suggest distinct epidemiologic pathways underlying AMR selection. For instance, our meta-analysis of data from hospital-based studies did not identify consistent associations of AMR prevalence with in-hospital antibiotic use. We may expect these associations to be weak if resistant infections are often imported from the community, such that antibiotic use in hospital settings accounts only partially for selective pressure. Additionally, it is important to note that use patterns measured in the hospital-based studies do not represent individual patients’ treatment histories for individuals whose infections were characterized as drug-resistant or susceptible.

Prevalence of AMR within organisms such as *Streptococcus pyogenes*, *Enterococcus faecium*, and *Enterococcus faecalis* was weakly associated with differences in antibiotic use in comparison to our observations for other pathogens. All three are less prevalently carried than the organisms listed above, and their weaker AMR elasticity with respect to national-level antibiotic use may reflect a scenario in which resistance selection typically occurs through direct treatment of infections rather than bystander exposures [40]. In contrast, *Acinetobacter* spp. was associated with greater elasticity estimates than other pathogens for nearly all drug classes. Although consistent with the high genomic plasticity of *Acinetobacter* spp., which facilitates spontaneous, highly efficient recombination and horizontal gene transfer [62,63], epidemiologic pathways facilitating resistance selection in this organism remain poorly understood [64].

Speculatively interpreting elasticity coefficients in our study as causal estimates of the effects of antibiotic consumption on AMR prevalence, our findings suggest that a 10% reduction in antibiotic use [34]—achievable through prevention of infections through vaccination and WASH interventions in LMIC settings [33], and consistent with reductions in antibiotic use achievable through enhanced stewardship interventions in higher-income settings [51–54]—would be expected to prevent roughly 38,000 annual deaths attributable to AMR [1]. This estimate corresponds to a scenario in which the same infections, counterfactually, were drug-susceptible (and thus responsive to available treatment). While the estimated difference in burden is substantial, it is important to note that AMR is estimated to be a causal factor in <20% of deaths due to bacterial diseases [20], and the difference represents only 0.50% of all bacterial disease mortality currently estimated to occur annually. In a scenario where reductions in antibiotic use are concentrated in drugs with high elasticity, our estimate of the difference increases to 110,000 AMR-attributable deaths, which represents 1.5% of bacterial disease mortality. These figures assume that the relationship between antibiotic use and AMR is symmetric between increases and decreases—if decreases in fact have a smaller effect on AMR than increases, then there is lower effective elasticity for decreases and the difference in burden may be closer to our low-elasticity scenario estimate of 20,000 deaths. On the other hand, feedback loops in AMR dynamics could lead to higher effective elasticity [65], bringing the difference closer to our high-elasticity scenario of 74,000 deaths.

Quantitative estimates of impacts on deaths attributable to AMR, and on bacterial disease deaths generally, should inform assessments of the benefits and harms of permissive or restrictive antibiotic use interventions. For instance, several recent trials have evaluated the effects of antibiotic prophylaxis with azithromycin on mortality in differing populations within LMICs. Within low-income African settings, community-based mass drug administration reduced all-cause infant mortality by 25% [24] and reduced all-cause mortality among young children by 13%–18% [25,26]. Across African, Asian, and Latin American countries, azithromycin prophylaxis in pregnant mothers reduced the risk of maternal sepsis or death due to any cause by 33% [27]. While specific to the study settings, the prevented burden of mortality in these studies likely exceeds the burden of AMR-attributable deaths that our elasticity estimates suggest could result from the associated increases in antibiotic use. Follow-up studies of AMR prevalence in colonizing bacterial pathogens from the intervention communities demonstrate increases in AMR prevalence [66,67], although with limited spillover geographically [68] and across age groups [69]. Community-based mass drug administration represents an extreme scenario for increasing antibiotic use which may thus be associated with greater risks of AMR selection than other interventions targeting antibiotics to individuals with suspected bacterial infections. However, findings from these studies—that benefits of mortality prevention outweigh potential harms driven by resistance selection—align with our findings that differences in AMR-attributable mortality across settings with differing antibiotic use are modest relative to differences in overall bacterial disease mortality.

Our study has several limitations. While analyzing data at the level of country-years enabled us to assess patterns across a broad range of epidemiologically distinct settings, this coarse level of aggregation may fail to capture relationships between antibiotic use reported at more focal geographical [6,11] and temporal resolutions [5,70]. Since data are reported at the country level, our analysis is vulnerable to the ecological fallacy, and the observed population-level associations may not map to smaller units of aggregation. The specific clinical settings represented within the AMR surveillance data we analyzed may have differed by country, in association with differences in patients’ access to healthcare access as well as criteria informing specimen collection in differing countries. Antibiotic consumption estimates used for our primary analyses were generated through analyses of multiple input data sources with gaps in coverage across geography, time, and clinical settings [41]; however, analyses relating AMR prevalence directly to antibiotic sales data provided similar qualitative conclusions. Finally, our model is a static statistical model that does not incorporate dynamical aspects of the disease transmission and pathogen evolution processes, meaning that it cannot be used to predict the impact of interventions over long time periods, or potential asymmetric effects of increasing versus reducing consumption. Cumulative effects of bacterial disease spread or AMR evolution over time may lead to non-linearities and feedback loops which cause the impact of antibiotic use to deviate from our estimates. However, absent detailed mechanistic understanding of these underlying processes, the phenomenological relationships between antibiotic use and AMR identified by our analyses provide a first-order basis for anticipating impacts of changes in antibiotic use.

Aligning antibiotic availability with need remains central to efforts aimed at mitigating the burden of severe bacterial infections. While antibiotic consumption has expanded in recent decades in various upper- and lower-middle-income countries [42,71,72], gaps in availability and use of appropriate drugs persist [18,73]. Lack of antibiotic access thus remains a significant contributor to mortality in some of the lowest-income settings globally [74], and appropriate levels of antibiotic use in such settings should not be dictated by standards from HICs with lower disease burden [55]. By demonstrating elasticity in AMR to changes in drug consumption, our study complements others addressing the roles of socioeconomic, environmental, healthcare, and governance factors in AMR burden [30,32,75,76]. While associations we identify between drug-specific consumption and AMR prevalence suggest the burden of disease attributable to AMR is modifiable, anticipated impacts of reduced antibiotic consumption must also be weighed against potential effects of reduced antibiotic access on the global burden of bacterial diseases [1,20]. Quantitative assessments of the impacts of changes in antibiotic use on AMR burden merit consideration when weighing the benefits and potential harms of interventions modifying antibiotic use.

## Supporting information

S1 Checklist GATHER checklist. The GATHER checklist template may be obtained from its original source at https://doi.org/10.1016/S0140-6736(16)30388-9 or https://www.who.int/data/gather.
(PDF)

S2 Checklist RECORD checklist. (PDF)

S1 Table Included data for estimation of elasticity by pathogen and drug class. (DOCX)

S2 Table Results of permutation tests for class specificity in the relationship between antimicrobial resistance prevalence and antibiotic consumption. (DOCX)

S3 Table Studies assessing the relationship between within-hospital antimicrobial consumption and resistance prevalence. (DOCX)

S4 Table A measure of distortion of AMR due to continuity correction in each pathogen and antibiotic class, ordered by total deaths attributable to AMR. (DOCX)

S5 Table Loadings of World Bank Health, Nutrition, and Population Statistics variables for the first principal component. (DOCX)

S6 Table Loadings of World Bank Health, Nutrition, and Population Statistics for the second principal component. (DOCX)

S7 Table Loadings of World Bank Health, Nutrition, and Population Statistics for the third principal component. (DOCX)

S8 Table Correlation between consumption and other model variables for each antibiotic class. (DOCX)

S9 Table Measures of model fit for each regression at the antibiotic class-pathogen level, along with *p*-values of the White test for homoskedasticity. (DOCX)

S10 Table Measures of model fit for each regression at the pathogen level, along with *p*-values of the White test for homoskedasticity. (DOCX)

S11 Table Measures of model fit for each regression at the antibiotic class level, along with *p*-values of the White test for homoskedasticity. (DOCX)

S12 Table Influential observations with Cook’s distance greater than 1. (DOCX)

S13 Table Type II ANOVA measure of variance explained by each variable for each regression at the antibiotic class-pathogen level. (DOCX)

S14 Table Variance inflation factor measure of collinearity by each variable for each regression at the antibiotic class-pathogen level. (DOCX)

S15 Table Elasticity, variance inflation factor measure of collinearity, and type II ANOVA measure of variance explained by each variable for each regression at the antibiotic class-pathogen level, with PC2 and PC3 excluded. (DOCX)

S16 Table Elasticity estimates from each regression at the antibiotic class-pathogen level, under various sensitivity analyses. (DOCX)

S17 Table Calculated elasticities with log-odds coefficients in binomial and beta-binomial models. (DOCX)

S18 Table Difference in AMR burden estimated using elasticities calculated under different sensitivity analyses. (DOCX)

S1 Fig Distribution of percent resistant isolates in unweighted and weighted data. (PDF)

S2 Fig Distribution of covariates in all countries compared to the distribution in observations used for the regression analyses. (PDF)

S3 Fig Distribution of antibiotic consumption in all consumption data compared to the distribution across country-years included in the regression analyses. (PDF)

S4 Fig Difference in the frequency of increases and decreases in AMR prevalence seen within countries over different time horizons, by antibiotic class and pathogen combination. (PDF)

S5 Fig Directed acyclic graph. (PDF)

S6 Fig Scree plot of the principal component analysis of the World Bank country-level variables. (PDF)

S7 Fig Distribution of resistance levels observed for each antibiotic class-pathogen combination. (PDF)

S8 Fig Scatterplot of AMR attributable burden and the continuity factor. (PDF)

S9 Fig Residuals plots by antibiotic consumption for the main regression analysis by antibiotic class-pathogen combination. (PDF)

S10 Fig Elasticity by antibiotic class, stratified by country income stratum. (PDF)

S11 Fig Elasticity by pathogen, stratified by country income stratum. (PDF)

S12 Fig Pathogen- and class-specific elasticity, calculated using subclass-level resistance measurements. (PDF)

S13 Fig Pathogen- and class-specific elasticity, calculated including only the first principal component of WBHNP data in the adjustment set. (PDF)

S14 Fig Pathogen- and class-specific elasticity, calculated excluding influential observations with Cook’s distance greater than 1. (PDF)

S15 Fig Pathogen- and class-specific elasticity, calculated on the log-odds scale using a beta-binomial regression model. (PDF)

S16 Fig Pathogen- and class-specific elasticity, calculated using raw aggregated measures of antibiotic use from the IQVIA MIDAS dataset. (PDF)

S17 Fig Pathogen- and class-specific elasticity, calculated using within-hospital measures of antibiotic use and prevalence of AMR among infections observed in the same facilities. (PDF)

S18 Fig Counterfactual burden of AMR-attributable mortality, by region. (PDF)

S19 Fig Counterfactual burden of AMR-attributable mortality, by pathogen and region. (PDF)

S1 Text List of inclusions and aliases in antibiotic classes. (PDF)

S2 Text List of countries (identified by ISO3 codes) included in the study, by pathogen. (PDF)

S3 Text List of countries (identified by ISO3 codes) included in the study using raw IQVIA consumption data, by pathogen. (PDF)

S4 Text Models accessed via GitHub copilot to support statistical programming. (PDF)

## Abbreviations

AMR: Antimicrobial resistance
ATC: anatomical therapeutic classification
ATLAS: Antimicrobial Testing Leadership and Surveillance
AWaRe: Access, Watch, Reserve
BIC: Bayesian Information Criterion
CAESAR: Central Asian and European Surveillance of Antimicrobial Resistance
ECDC: European Centre for Disease Prevention and Control
HICs: high-income countries
LMICs: low- and middle-income countries
WASH: water, sanitation, and hygiene
WBHNP: World Bank Health, Nutrition, and Population Statistics
