# Supplementary material for: Burden of bacterial antimicrobial resistance associated with modifiable human antibiotic consumption: An observational study
Source: PLoS Med. 2026 Oct 26;23(10):e1005140. doi: 10.1371/journal.pmed.1005140 (PMC13719055; doi:10.1371/journal.pmed.1005140)
Supplement: Supplementary file 2 [file pmed.1005140.s002.pdf]

The RECORD statement – checklist of items, extended from the STROBE statement, that should be reported in observational studies using routinely collected health data.

	Item No.	STROBE items	RECORD items	Location in manuscript where items are reported
	1	(a) Indicate the study's design with a commonly used term in the title or the abstract (b) Provide in the abstract an informative and balanced summary of what was done and what was found	RECORD 1.1: The type of data used should be specified in the title or abstract. When possible, the name of the databases used should be included.<br><br>RECORD 1.2: If applicable, the geographic region and timeframe within which the study took place should be reported in the title or abstract.<br><br>RECORD 1.3: If linkage between databases was conducted for the study, this should be clearly stated in the title or abstract.	Abstract, beginning of Methods and Findings (As there are multiple distinct data sources, we enumerate these specifically in the main text rather than the abstract)
Background rationale	2	Explain the scientific background and rationale for the investigation being reported		Introduction, paragraphs 2 and 3.
Objectives	3	State specific objectives, including any prespecified hypotheses		Introduction, paragraph 3.
Study Design	4	Present key elements of study design early in the paper		Methods, *Regression*.
Setting	5	Describe the setting, locations, and relevant dates, including periods of recruitment, exposure, follow-up, and data collection		Specified as global scope, measured at country-year resolution, over period 2000–2018 (Introduction paragraph 3, Methods, *Data sources*, paragraph 1); specified as 67 countries for secondary analyses with IQVIA data (Methods, *Data sources*, paragraph 3).

Participants	6	*(a) Cohort study* - Give the eligibility criteria, and the sources and methods of selection of participants. Describe methods of follow-up<br>*Case-control study* - Give the eligibility criteria, and the sources and methods of case ascertainment and control selection. Give the rationale for the choice of cases and controls<br>*Cross-sectional study* - Give the eligibility criteria, and the sources and methods of selection of participants<br><br>*(b) Cohort study* - For matched studies, give matching criteria and number of exposed and unexposed<br>*Case-control study* - For matched studies, give matching criteria and the number of controls per case	RECORD 6.1: The methods of study population selection (such as codes or algorithms used to identify subjects) should be listed in detail. If this is not possible, an explanation should be provided.<br><br>RECORD 6.2: Any validation studies of the codes or algorithms used to select the population should be referenced. If validation was conducted for this study and not published elsewhere, detailed methods and results should be provided.<br><br>RECORD 6.3: If the study involved linkage of databases, consider use of a flow diagram or other graphical display to demonstrate the data linkage process, including the number of individuals with linked data at each stage.	N/A: Data were pre-aggregated from population-based surveillance sources (antimicrobial resistance surveillance in bacterial infections), from prior model-based estimates of antibiotic use (GRAM data), and from aggregated antimicrobial sales (IQVIA). We did not have access to individual-level data to construct study populations via any algorithmic approach. We describe eligibility criteria for inclusion of country-years on given drug/bug combinations in Methods, *Data sources*, paragraph 2.
Variables	7	Clearly define all outcomes, exposures, predictors, potential confounders, and effect modifiers. Give diagnostic criteria, if applicable.	RECORD 7.1: A complete list of codes and algorithms used to classify exposures, outcomes, confounders, and effect modifiers should be provided. If these cannot be reported, an explanation should be provided.	Methods, *Data sources*, paragraph 2 (ATC codes for antibiotic classes).
Data sources/ measurement	8	For each variable of interest, give sources of data and details of methods of assessment (measurement).<br>Describe comparability of assessment methods if there is more than one group		Methods, *Data sources*, and Methods, *Within-hospital antibiotic use estimates*. Comparability described in Methods, *Regression*, paragraph 4.

Bias	9	Describe any efforts to address potential sources of bias		Methods, *Regression*, paragraph 3 (adjustment); Methods, *Permutation testing for specificity of consumption and AMR relationships*.
Study size	10	Explain how the study size was arrived at		Methods, *Data Sources*.
Quantitative variables	11	Explain how quantitative variables were handled in the analyses. If applicable, describe which groupings were chosen, and why		Methods, *Regression*.
Statistical methods	12	(a) Describe all statistical methods, including those used to control for confounding<br>(b) Describe any methods used to examine subgroups and interactions<br>(c) Explain how missing data were addressed<br>(d) *Cohort study* - If applicable, explain how loss to follow-up was addressed<br>*Case-control study* - If applicable, explain how matching of cases and controls was addressed<br>*Cross-sectional study* - If applicable, describe analytical methods taking account of sampling strategy<br>(e) Describe any sensitivity analyses		(a) Methods, *Regression*<br>(b) Methods, *Regression*, paragraph 4<br>(c) Methods, *Regression*, paragraph 3<br>(d) N/A (no matching, no loss to follow-up; limitation acknowledged of reliance on country-years with available data (Methods, *Regression*, paragraph 3).<br>(e) Permutation testing (Methods, *Permutation testing*); IQVIA data analysis (Methods, *Data sources*, paragraph 3); in-hospital antibiotic use

				analysis (Methods, *Within-hospital*)
Data access and cleaning methods		..	RECORD 12.1: Authors should describe the extent to which the investigators had access to the database population used to create the study population.<br><br>RECORD 12.2: Authors should provide information on the data cleaning methods used in the study.	Specified that authors did not have access to underlying individual-level information (just reported counts) for this analysis (Methods, *Reporting and transparency*).
Linkage		..	RECORD 12.3: State whether the study included person-level, institutional-level, or other data linkage across two or more databases. The methods of linkage and methods of linkage quality evaluation should be provided.	N/A (no linkage possible; study lacked individual-level data)
Participants	13	(a) Report the numbers of individuals at each stage of the study (*e.g.*, numbers potentially eligible, examined for eligibility, confirmed eligible, included in the study, completing follow-up, and analysed)<br>(b) Give reasons for non-participation at each stage.<br>(c) Consider use of a flow diagram	RECORD 13.1: Describe in detail the selection of the persons included in the study (*i.e.,* study population selection) including filtering based on data quality, data availability and linkage. The selection of included persons can be described in the text and/or by means of the study flow diagram.	Table S1 (numbers of country-years and isolates included, by pathogen and drug); Table S4 (studies included in within-hospital antibiotic use analysis).
Descriptive data	14	(a) Give characteristics of study participants (*e.g.*, demographic, clinical, social) and information on exposures and potential confounders<br>(b) Indicate the number of participants with missing data for each variable of interest		Supporting information text S2, S3: countries included for each analysis, by pathogen; Results, *Data characteristics* for narrative description.

		(c) *Cohort study* - summarise follow-up time (*e.g.*, average and total amount)		
Outcome data	15	*Cohort study* - Report numbers of outcome events or summary measures over time<br>*Case-control study* - Report numbers in each exposure category, or summary measures of exposure<br>*Cross-sectional study* - Report numbers of outcome events or summary measures		N/A —no outcome events or exposure categories analogous to a conventional observational epidemiological study.
Main results	16	(a) Give unadjusted estimates and, if applicable, confounder-adjusted estimates and their precision (e.g., 95% confidence interval). Make clear which confounders were adjusted for and why they were included<br>(b) Report category boundaries when continuous variables were categorized<br>(c) If relevant, consider translating estimates of relative risk into absolute risk for a meaningful time period		(a) Figure 1; Results, *Relationship between antibiotic consumption and resistance*<br>(b) N/A (no categorical variables)<br>(c) Present absolute estimates of avertible mortality in Figure 3; Results, *Avertible burden of deaths due to resistance*.
Other analyses	17	Report other analyses done—e.g., analyses of subgroups and interactions, and sensitivity analyses		Results, *Relationship between antibiotic consumption and resistance*, paragraph 4–5, Results, *Specificity of antibiotic use as a predictor of resistance prevalence*, Results, *Relationship between antibiotic consumption in hospitals and resistance*

Key results	18	Summarise key results with reference to study objectives		Discussion, paragraphs 1 and 4
Limitations	19	Discuss limitations of the study, taking into account sources of potential bias or imprecision. Discuss both direction and magnitude of any potential bias	RECORD 19.1: Discuss the implications of using data that were not created or collected to answer the specific research question(s). Include discussion of misclassification bias, unmeasured confounding, missing data, and changing eligibility over time, as they pertain to the study being reported.	Discussion, paragraph 6
Interpretation	20	Give a cautious overall interpretation of results considering objectives, limitations, multiplicity of analyses, results from similar studies, and other relevant evidence		Discussion, paragraphs 1–5
Generalisability	21	Discuss the generalisability (external validity) of the study results		Discussion, paragraph 6
Funding	22	Give the source of funding and the role of the funders for the present study and, if applicable, for the original study on which the present article is based		Funding
Accessibility of protocol, raw data, and programming code		..	RECORD 22.1: Authors should provide information on how to access any supplemental information such as the study protocol, raw data, or programming code.	Methods, *Reporting and transparency*

*Reference: Benchimol EI, Smeeth L, Guttmann A, Harron K, Moher D, Petersen I, Sørensen HT, von Elm E, Langan SM, the RECORD Working Committee. The REporting of studies Conducted using Observational Routinely-collected health Data (RECORD) Statement. *PLoS Medicine* 2015; in press.

*Checklist is protected under Creative Commons Attribution (CC BY) license.
