# Supplementary material for: Burden of bacterial antimicrobial resistance associated with modifiable human antibiotic consumption: An observational study
Source: PLoS Med. 2026 Oct 26;23(10):e1005140. doi: 10.1371/journal.pmed.1005140 (PMC13719055; doi:10.1371/journal.pmed.1005140)
Supplement: Supplementary file 3 [file pmed.1005140.s021.pdf]

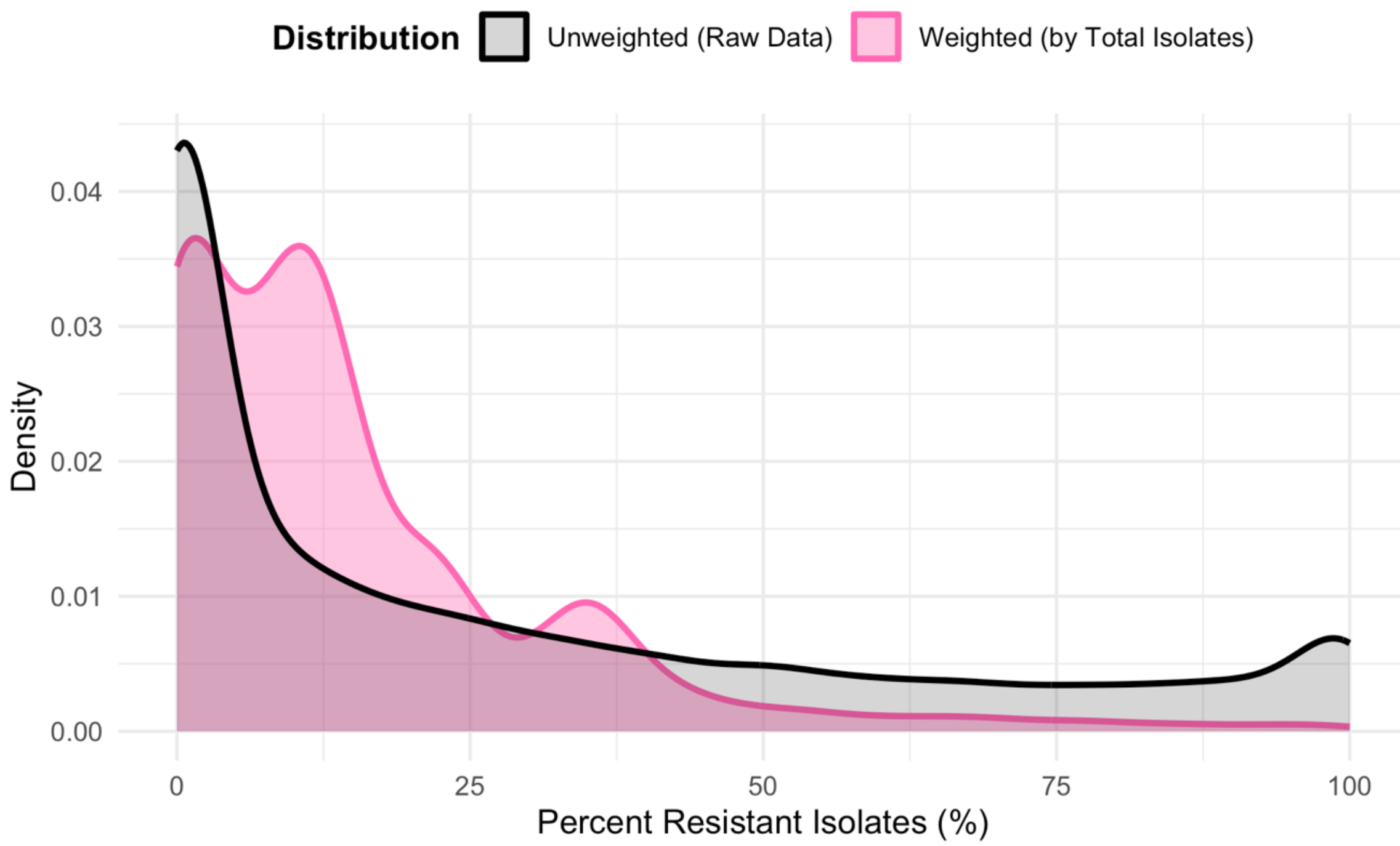

S1 Figure: Distribution of percent resistant isolates in unweighted and weighted data. We illustrate the distribution of the proportion of isolates exhibiting resistance, across country-year combinations for all pathogens and drugs. We distinguish distributions of values across all country-years (grey) from distributions accounting for weights by the count of isolates included (pink).
