# Supplementary material for: Burden of bacterial antimicrobial resistance associated with modifiable human antibiotic consumption: An observational study
Source: PLoS Med. 2026 Oct 26;23(10):e1005140. doi: 10.1371/journal.pmed.1005140 (PMC13719055; doi:10.1371/journal.pmed.1005140)
Supplement: Supplementary file 4 [file pmed.1005140.s022.pdf]

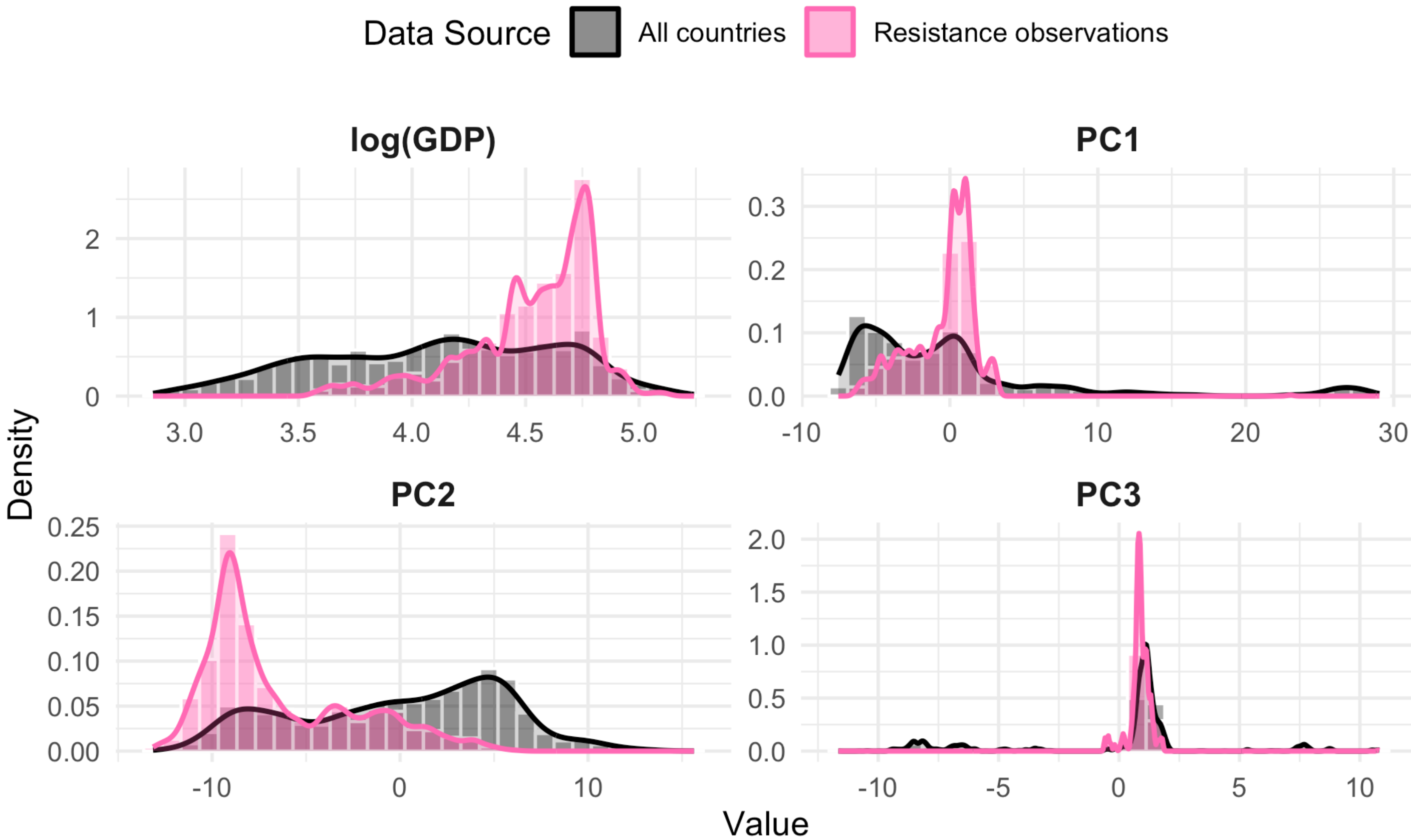

S2 Figure: Distribution of covariates in all countries compared to the distribution in observations used for the regression analyses. We illustrate distributions of each covariate across all country-years (grey) and across country-years for which resistance data were available (pink), which were thus included in analyses. Data in our analysis are biased towards countries with high GDP per capita. The values are also skewed for PC1 and PC2, which correlate strongly with GDP (**S5 Table**, **S6 Table**).
