# Supplementary material for: Burden of bacterial antimicrobial resistance associated with modifiable human antibiotic consumption: An observational study
Source: PLoS Med. 2026 Oct 26;23(10):e1005140. doi: 10.1371/journal.pmed.1005140 (PMC13719055; doi:10.1371/journal.pmed.1005140)
Supplement: Supplementary file 5 [file pmed.1005140.s023.pdf]

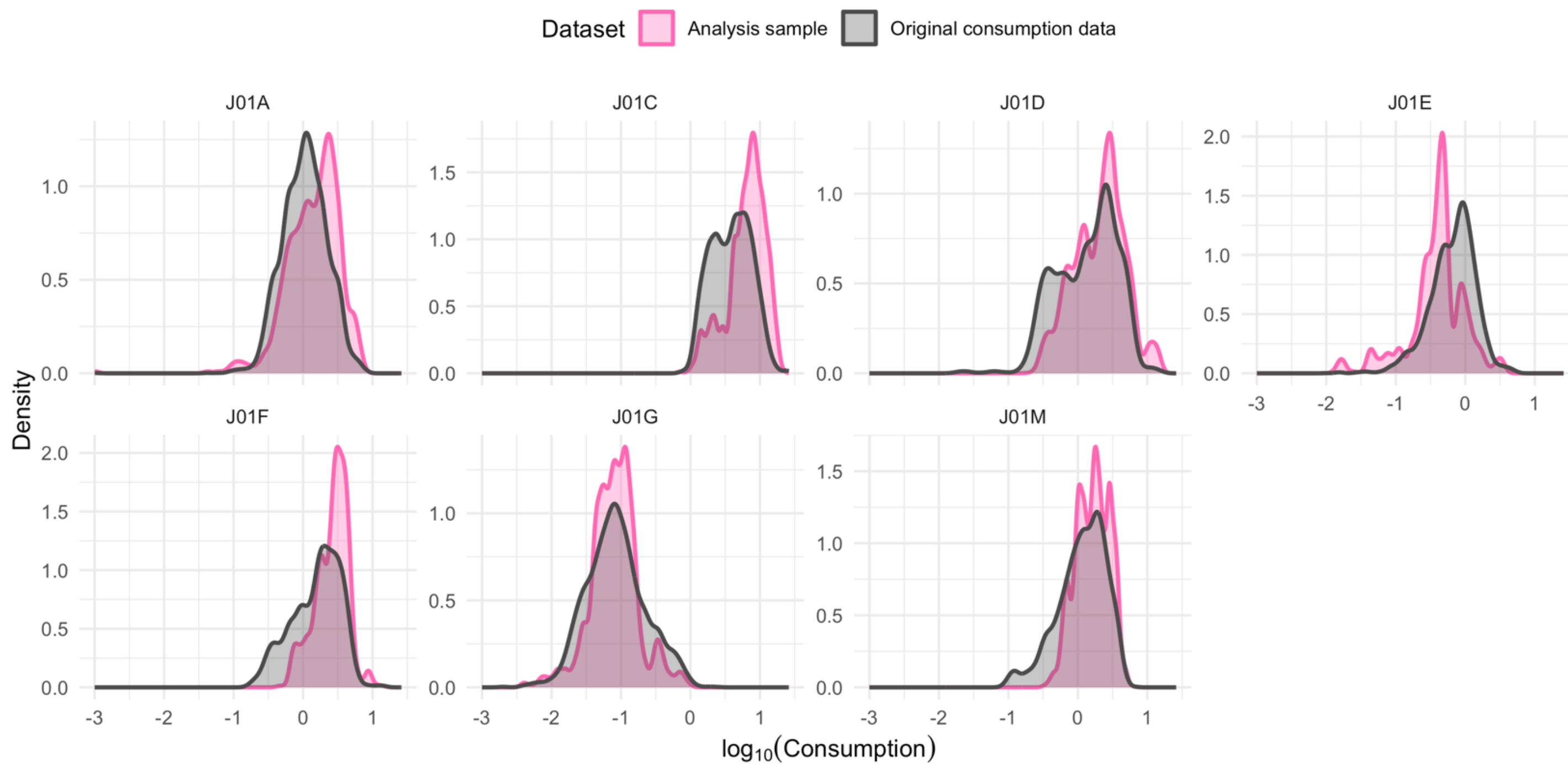

S3 Figure: Distribution of antibiotic consumption in all consumption data compared to the distribution across country-years included in the regression analyses. We illustrate distributions of rates of consumption (in DDD per person-year) for each drug class, distinguishing estimates across all country-years (grey) from those for which consumption data were available (pink), which were thus included in regression analyses.
