# Supplementary material for: Burden of bacterial antimicrobial resistance associated with modifiable human antibiotic consumption: An observational study
Source: PLoS Med. 2026 Oct 26;23(10):e1005140. doi: 10.1371/journal.pmed.1005140 (PMC13719055; doi:10.1371/journal.pmed.1005140)
Supplement: Supplementary file 6 [file pmed.1005140.s024.pdf]

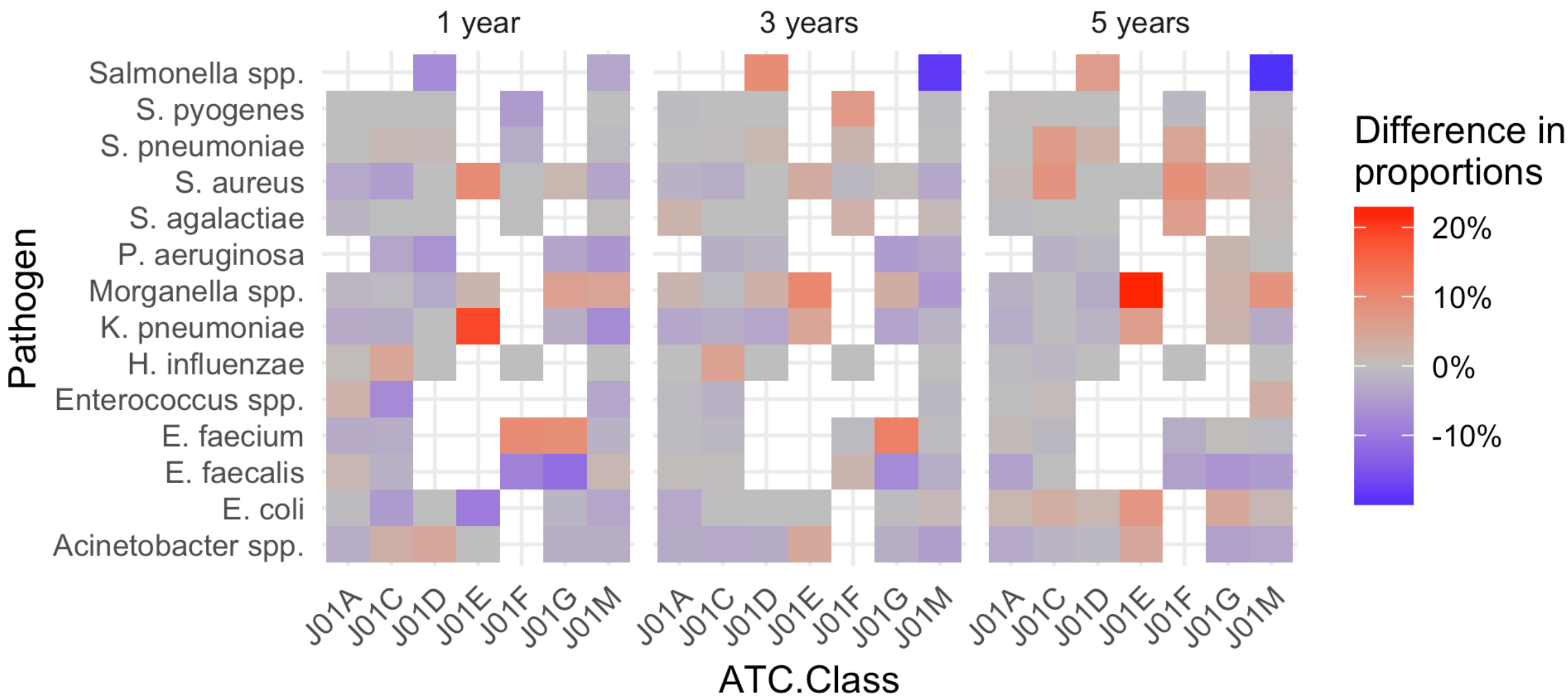

S4 Figure: Difference in the frequency of increases and decreases in AMR prevalence seen within countries over different time horizons, by antibiotic class and pathogen combination. Shaded values indicate differences in the proportion of country-year observations where, at the indicated time horizons, trends signal increases or decreases in the prevalence of resistance for each pathogen-drug combination. Values illustrated in grey signal balanced frequencies of increases and decreases; values in red and blue, respectively, indicate greater frequencies of increases and decreases.
