# Supplementary material for: Burden of bacterial antimicrobial resistance associated with modifiable human antibiotic consumption: An observational study
Source: PLoS Med. 2026 Oct 26;23(10):e1005140. doi: 10.1371/journal.pmed.1005140 (PMC13719055; doi:10.1371/journal.pmed.1005140)
Supplement: Supplementary file 7 [file pmed.1005140.s025.pdf]

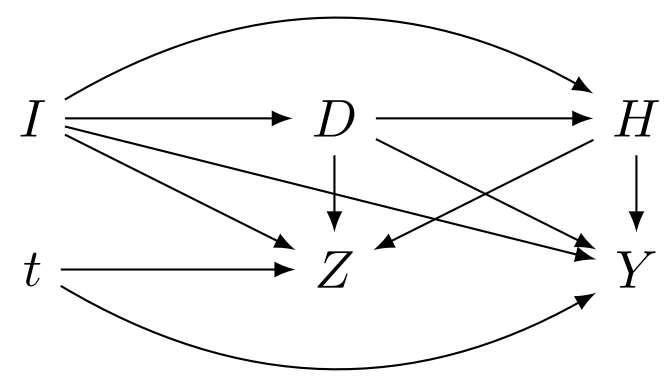

S5 Figure: Directed acyclic graph. We illustrate the causal process of interest to our analyses. We anticipate that income (*I*), development status (*D*), healthcare system capacity (*H*), and secular trends over time (*t*) impact both antibiotic use (*Z*) and AMR prevalence (*Y*). We further anticipate that income independently impacts both development status and healthcare system capacity, and that development status independently impacts healthcare system capacity. The causal relationship of interest is the effect of antibiotic use on AMR prevalence ($Z \rightarrow Y$). Estimation of this effect requires adjustment for *I*, *D*, *H*, and *t*. We measure *I* via countries' per-capita gross domestic product, and measure *t* by calendar year. Many candidate variables could proxy countries' development status and healthcare systems strength. We derive these variables via principal component decomposition of 173 indicators tracked across countries and over time by the World Bank Population Health, Nutrition, and Population database; primary analyses included the first 3 principal components (accounting for 59% of total variance across all indicators) and sensitivity analyses included the first 10, yielding similar results.
