# Supplementary material for: Burden of bacterial antimicrobial resistance associated with modifiable human antibiotic consumption: An observational study
Source: PLoS Med. 2026 Oct 26;23(10):e1005140. doi: 10.1371/journal.pmed.1005140 (PMC13719055; doi:10.1371/journal.pmed.1005140)
Supplement: Supplementary file 8 [file pmed.1005140.s026.pdf]

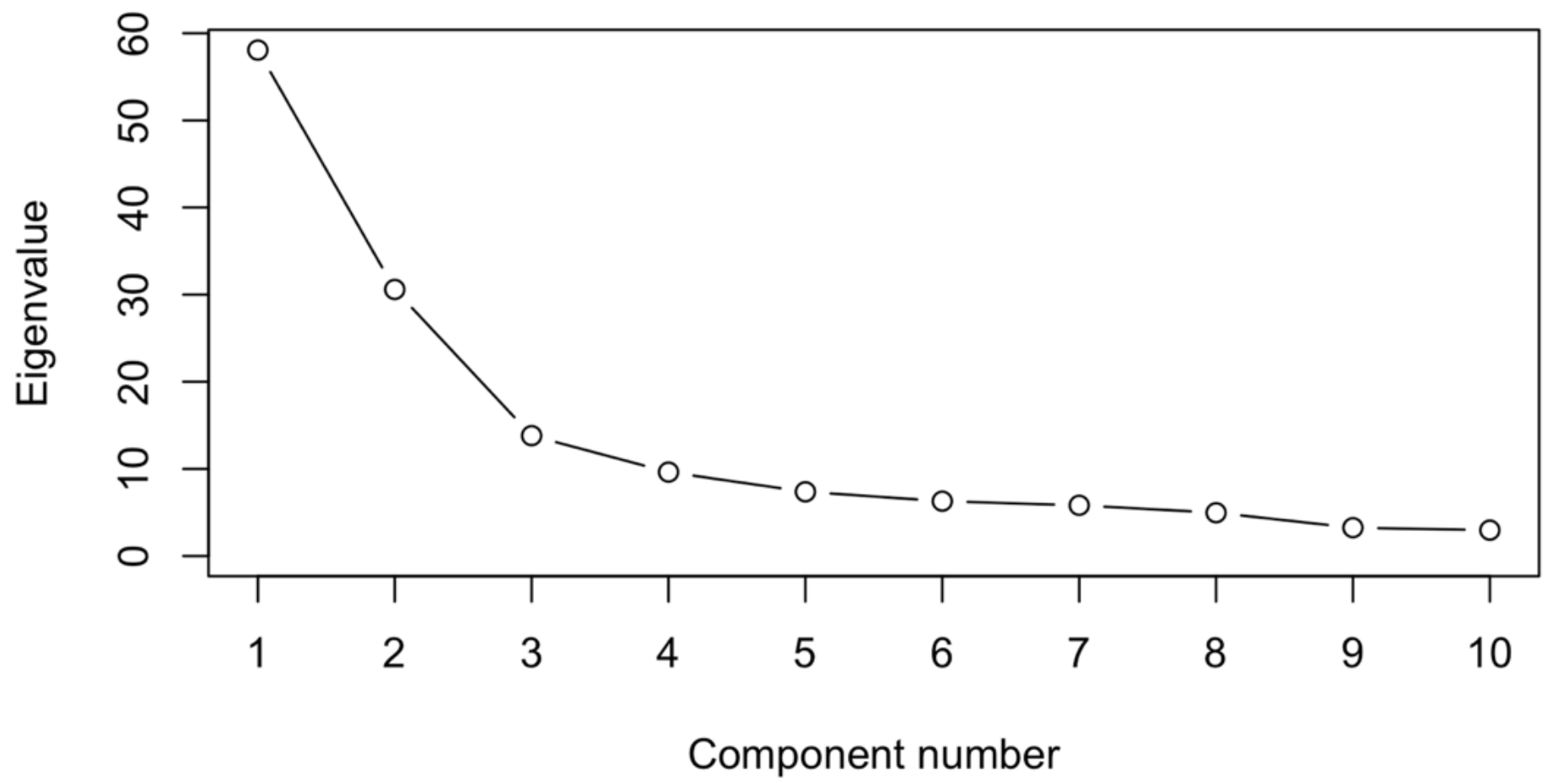

S6 Figure: Scree plot of the principal component analysis of the World Bank country-level variables. We present eigenvalues for principal components derived from 173 World Bank Health, Nutrition, and Population variables (with <30% of values missing).
