# Supplementary material for: Burden of bacterial antimicrobial resistance associated with modifiable human antibiotic consumption: An observational study
Source: PLoS Med. 2026 Oct 26;23(10):e1005140. doi: 10.1371/journal.pmed.1005140 (PMC13719055; doi:10.1371/journal.pmed.1005140)
Supplement: Supplementary file 9 [file pmed.1005140.s027.pdf]

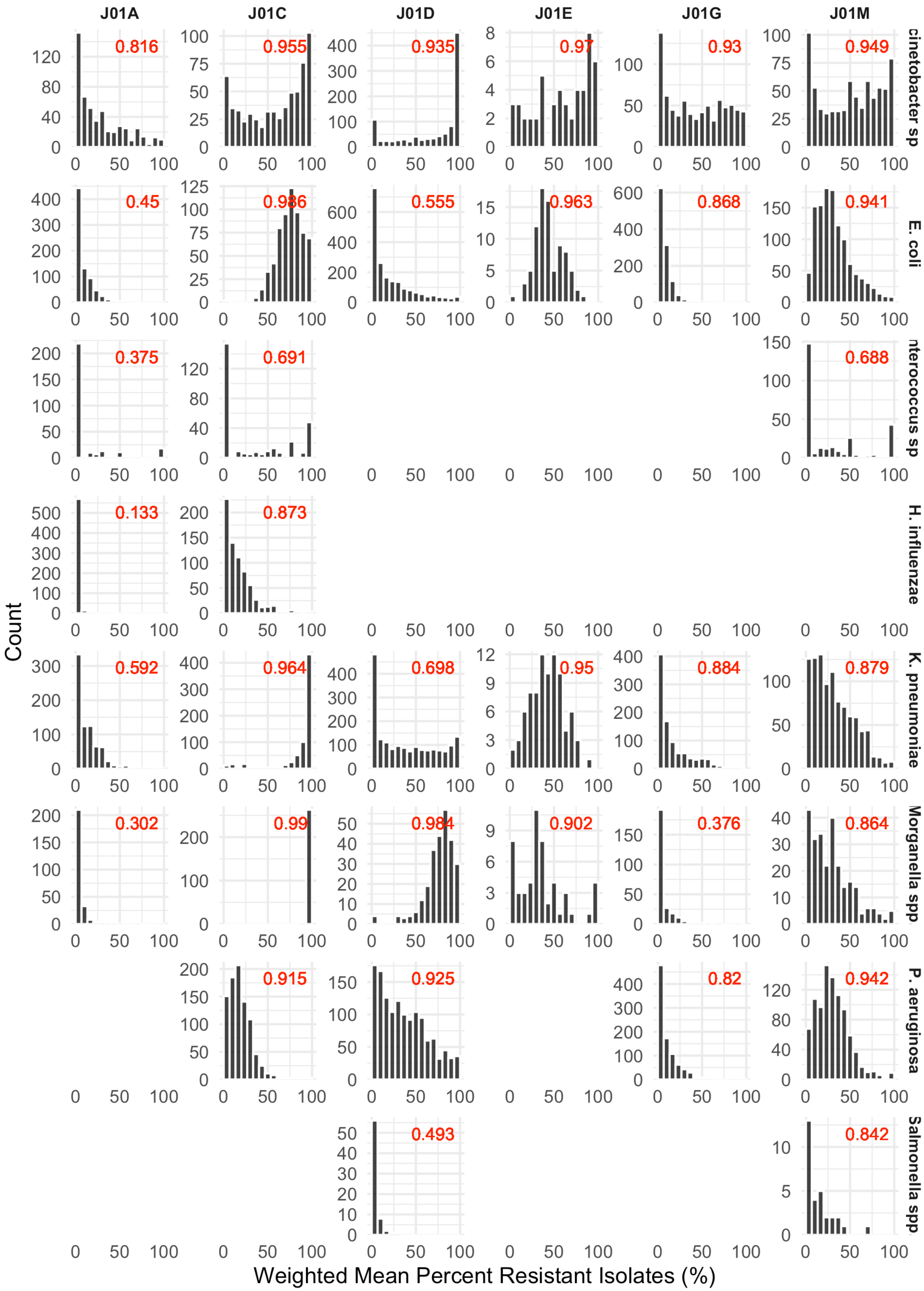

S7A Figure: Distribution of resistance levels observed for each antibiotic class-pathogen combination. Annotations give the continuity factor $\mathrm{mean}(\frac{y}{y+1})$ where y is the percent prevalence of AMR in each data point.

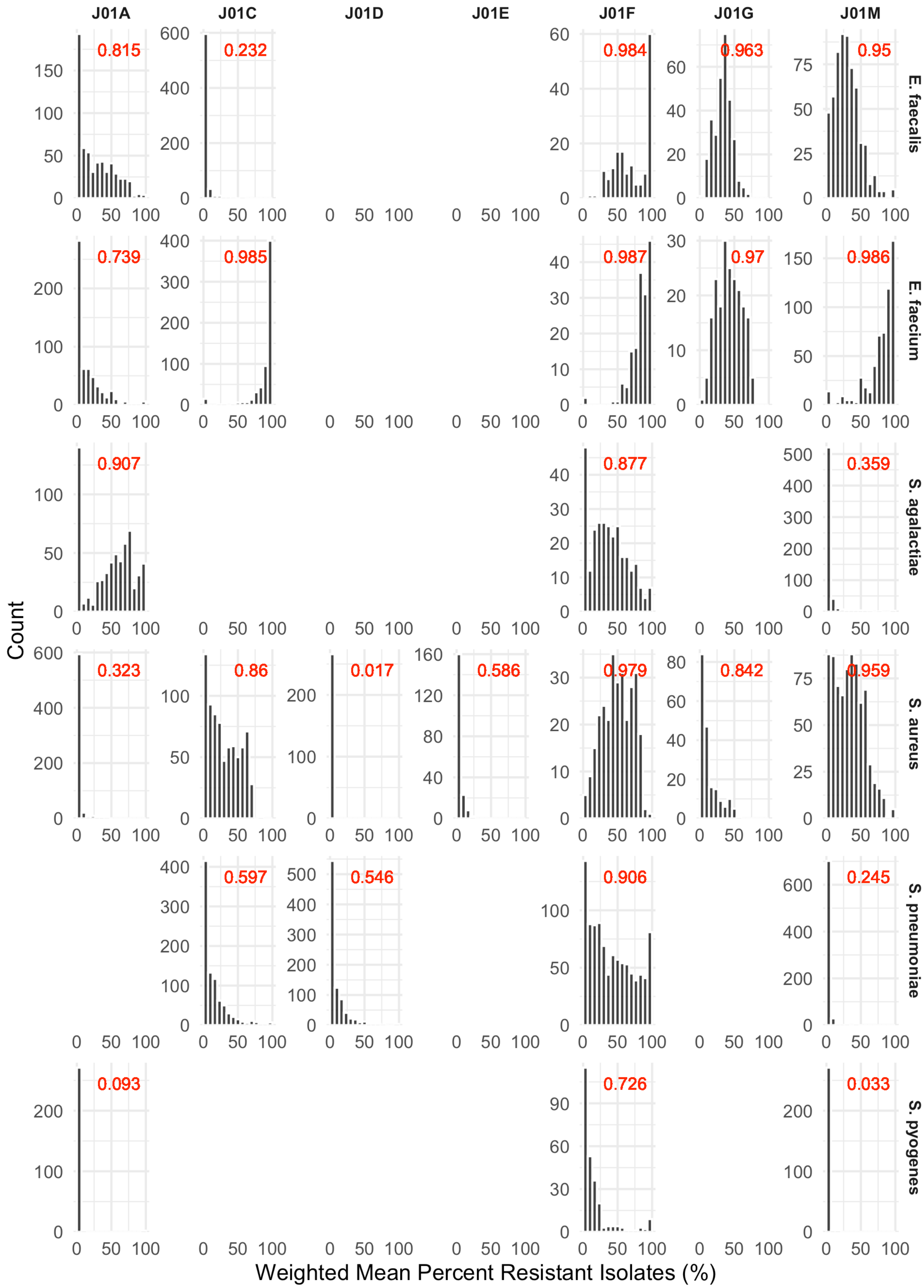

S7B Figure: Distribution of resistance levels observed for each antibiotic class-pathogen combination. Annotations give the continuity factor $\mathrm{mean}(\frac{y}{y+1})$ where y is the percent prevalence of AMR in each data point.
