# Supplementary material for: Burden of bacterial antimicrobial resistance associated with modifiable human antibiotic consumption: An observational study
Source: PLoS Med. 2026 Oct 26;23(10):e1005140. doi: 10.1371/journal.pmed.1005140 (PMC13719055; doi:10.1371/journal.pmed.1005140)
Supplement: Supplementary file 10 [file pmed.1005140.s028.pdf]

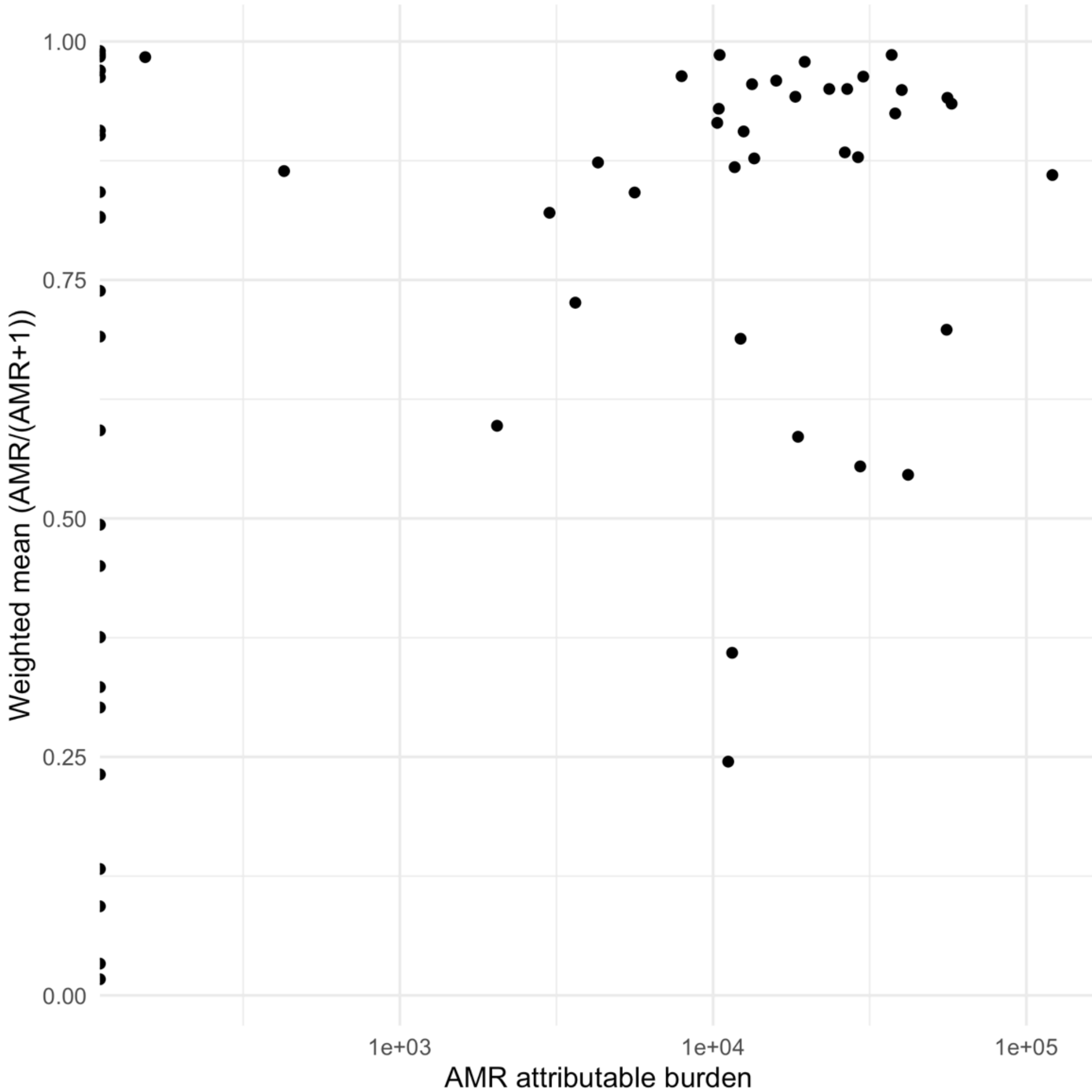

S8 Figure: Scatterplot of AMR attributable burden and the continuity factor. We illustrate the AMR-attributable burden (number of deaths due to resistance) against the mean of the continuity factor $\text{mean}\left(\frac{y}{y+1}\right)$, where y is the percent prevalence of AMR in each data point. Observations indicate that continuity factor values are generally closer to 1 for drug-pathogen combinations accounting for the greatest burden of resistant mortality.
