# Supplementary material for: Burden of bacterial antimicrobial resistance associated with modifiable human antibiotic consumption: An observational study
Source: PLoS Med. 2026 Oct 26;23(10):e1005140. doi: 10.1371/journal.pmed.1005140 (PMC13719055; doi:10.1371/journal.pmed.1005140)
Supplement: Supplementary file 11 [file pmed.1005140.s029.pdf]

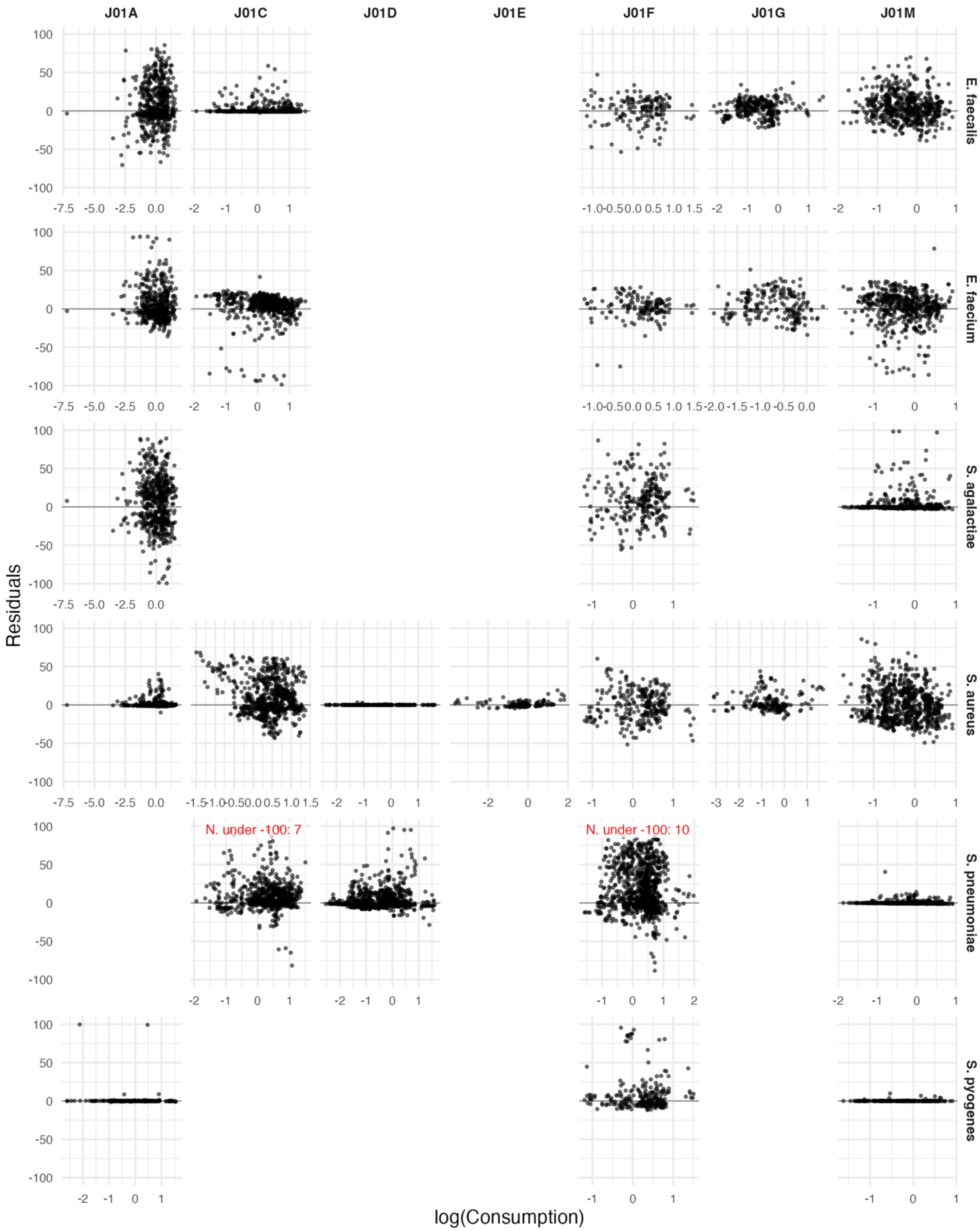

S9A Figure: Residuals plots by antibiotic consumption for the main regression analysis by antibiotic class-pathogen combination. Y axis limits are set at [100,-100]. Residuals not falling within these limits are numbered in the annotations (red text).

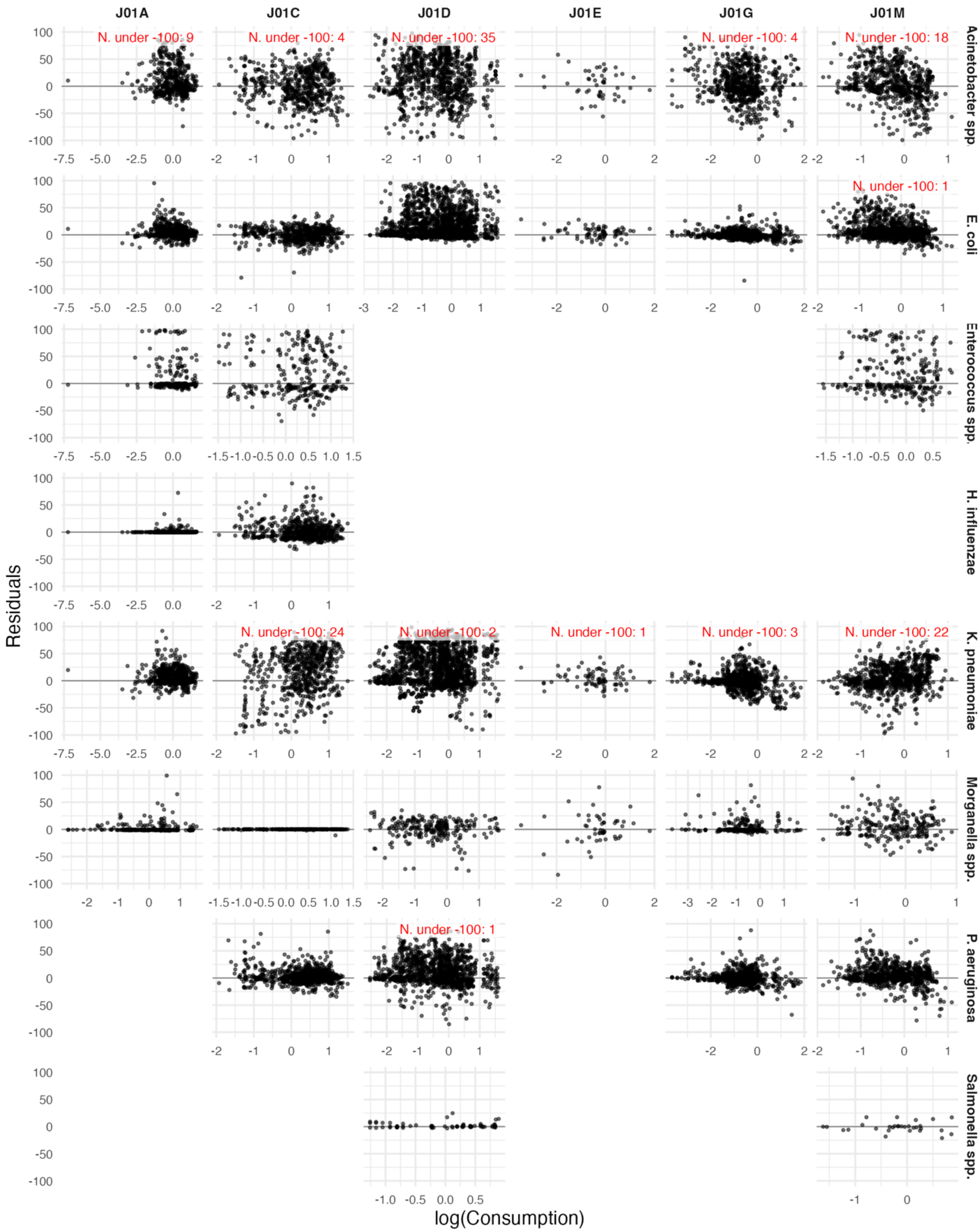

S9B Figure: Residuals plots by antibiotic consumption for the main regression analysis by antibiotic class-pathogen combination. Y axis limits are set at [100,-100]. Residuals not falling within these limits are numbered in the annotations (red text).
