# Supplementary material for: Burden of bacterial antimicrobial resistance associated with modifiable human antibiotic consumption: An observational study
Source: PLoS Med. 2026 Oct 26;23(10):e1005140. doi: 10.1371/journal.pmed.1005140 (PMC13719055; doi:10.1371/journal.pmed.1005140)
Supplement: Supplementary file 12 [file pmed.1005140.s030.pdf]

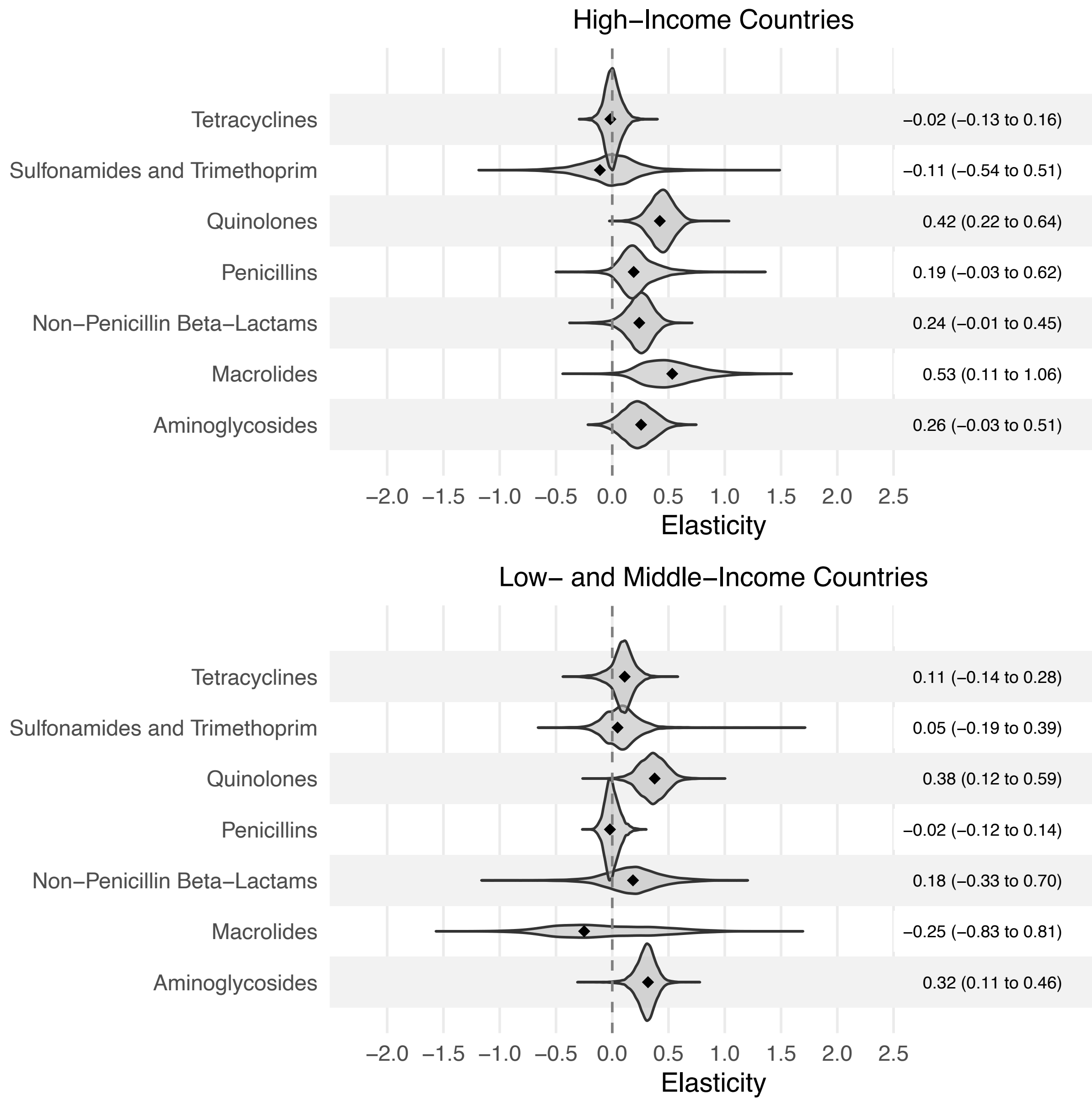

S10 Figure: Elasticity by antibiotic class, stratified by country income stratum. Violin plots illustrate bootstrapped distributions conveying estimated elasticity (with 95% confidence intervals) of AMR with respect to consumption of antibiotics, estimated for each antibiotic class in the data set, treating pathogen type as a source of random effects, with data separated for high-income countries and low- and middle-income countries. Reported values (right column) indicate estimates with 95% confidence intervals.
