# Supplementary material for: Burden of bacterial antimicrobial resistance associated with modifiable human antibiotic consumption: An observational study
Source: PLoS Med. 2026 Oct 26;23(10):e1005140. doi: 10.1371/journal.pmed.1005140 (PMC13719055; doi:10.1371/journal.pmed.1005140)
Supplement: Supplementary file 13 [file pmed.1005140.s031.pdf]

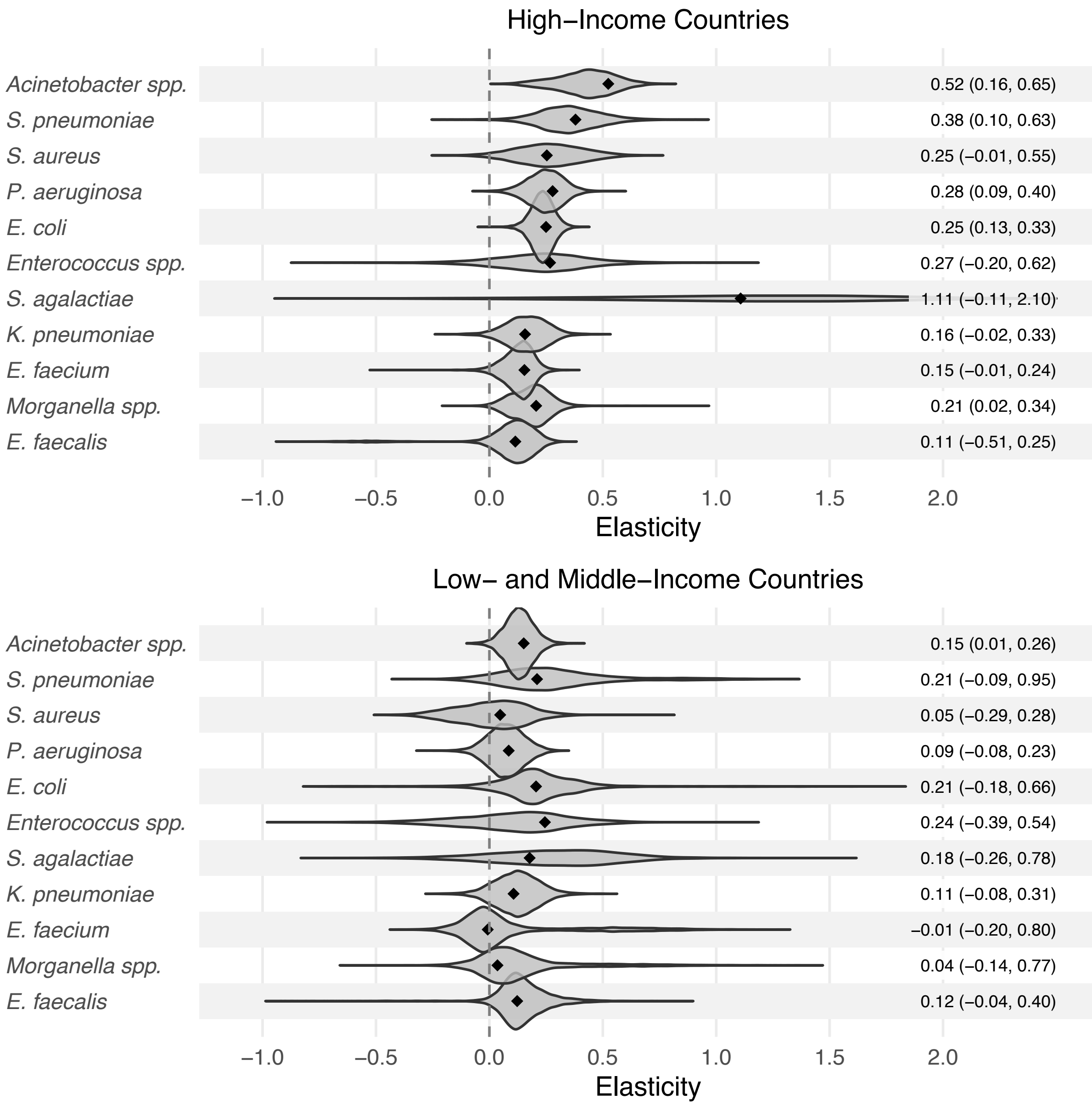

S11 Figure: Elasticity by pathogen, stratified by country income stratum. Forest plots illustrate bootstrapped distributions conveying estimated elasticity (with 95% confidence intervals) of AMR with respect to consumption of antibiotics, estimated for each pathogen in the data set, treating antibiotic class as a source of random effects, with data separated for high-income countries and low- and middle-income countries. Reported estimates (right column) report estimates with 95% confidence intervals.
