# Supplementary material for: Burden of bacterial antimicrobial resistance associated with modifiable human antibiotic consumption: An observational study
Source: PLoS Med. 2026 Oct 26;23(10):e1005140. doi: 10.1371/journal.pmed.1005140 (PMC13719055; doi:10.1371/journal.pmed.1005140)
Supplement: Supplementary file 18 [file pmed.1005140.s036.pdf]

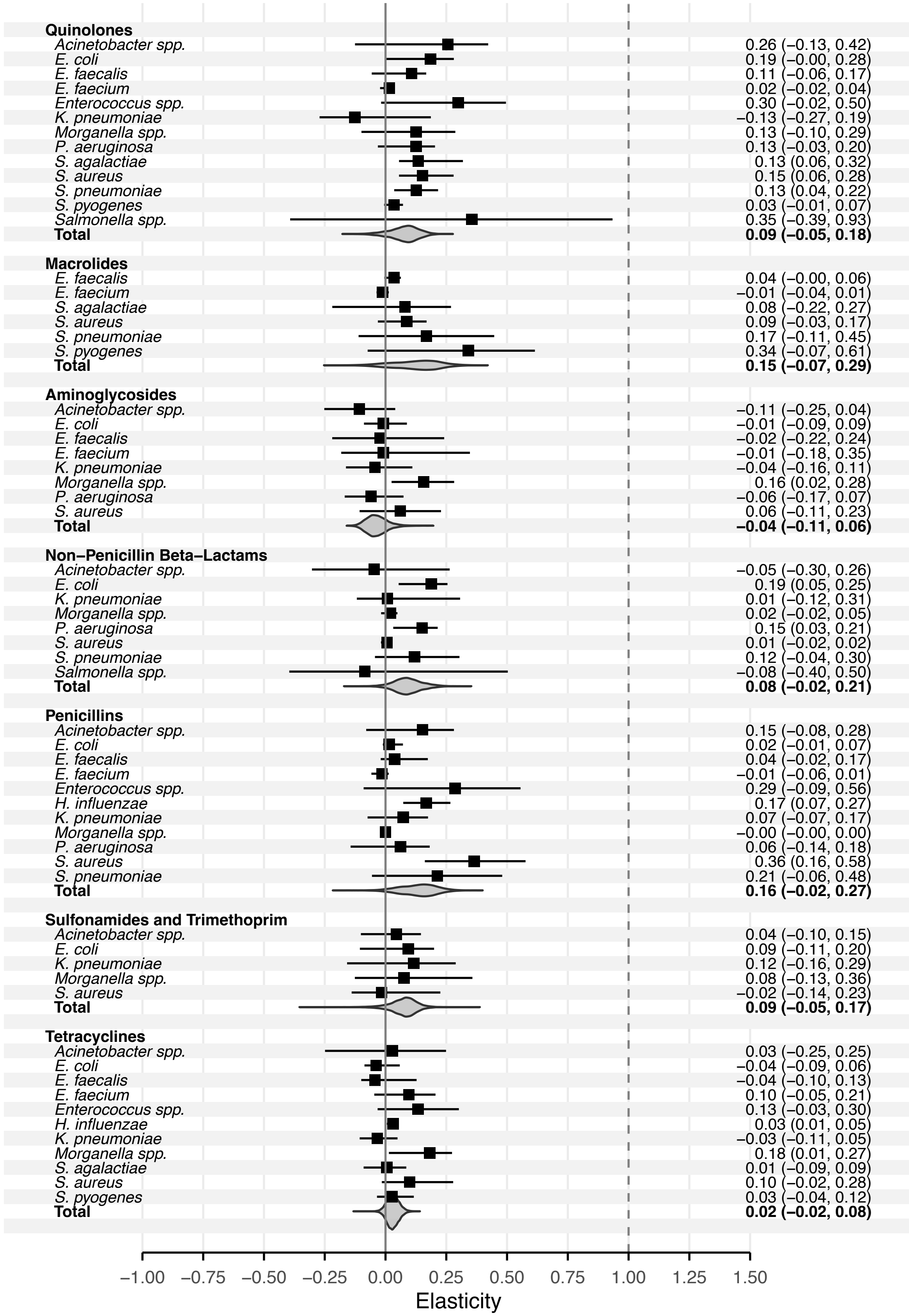

S16 Figure: Pathogen- and class-specific elasticity, calculated using raw aggregated measures of antibiotic use from the IQVIA MIDAS dataset. Forest plots illustrate distributions conveying estimated elasticity (with 95% confidence intervals) of AMR with respect to population-wide consumption of antibiotics. We plot estimates for specific combinations of pathogen and antibiotic class, as well as overall ("Total") estimates for each antibiotic class, treating pathogens as a source of random effects.
