# Supplementary material for: Burden of bacterial antimicrobial resistance associated with modifiable human antibiotic consumption: An observational study
Source: PLoS Med. 2026 Oct 26;23(10):e1005140. doi: 10.1371/journal.pmed.1005140 (PMC13719055; doi:10.1371/journal.pmed.1005140)
Supplement: Supplementary file 19 [file pmed.1005140.s037.pdf]

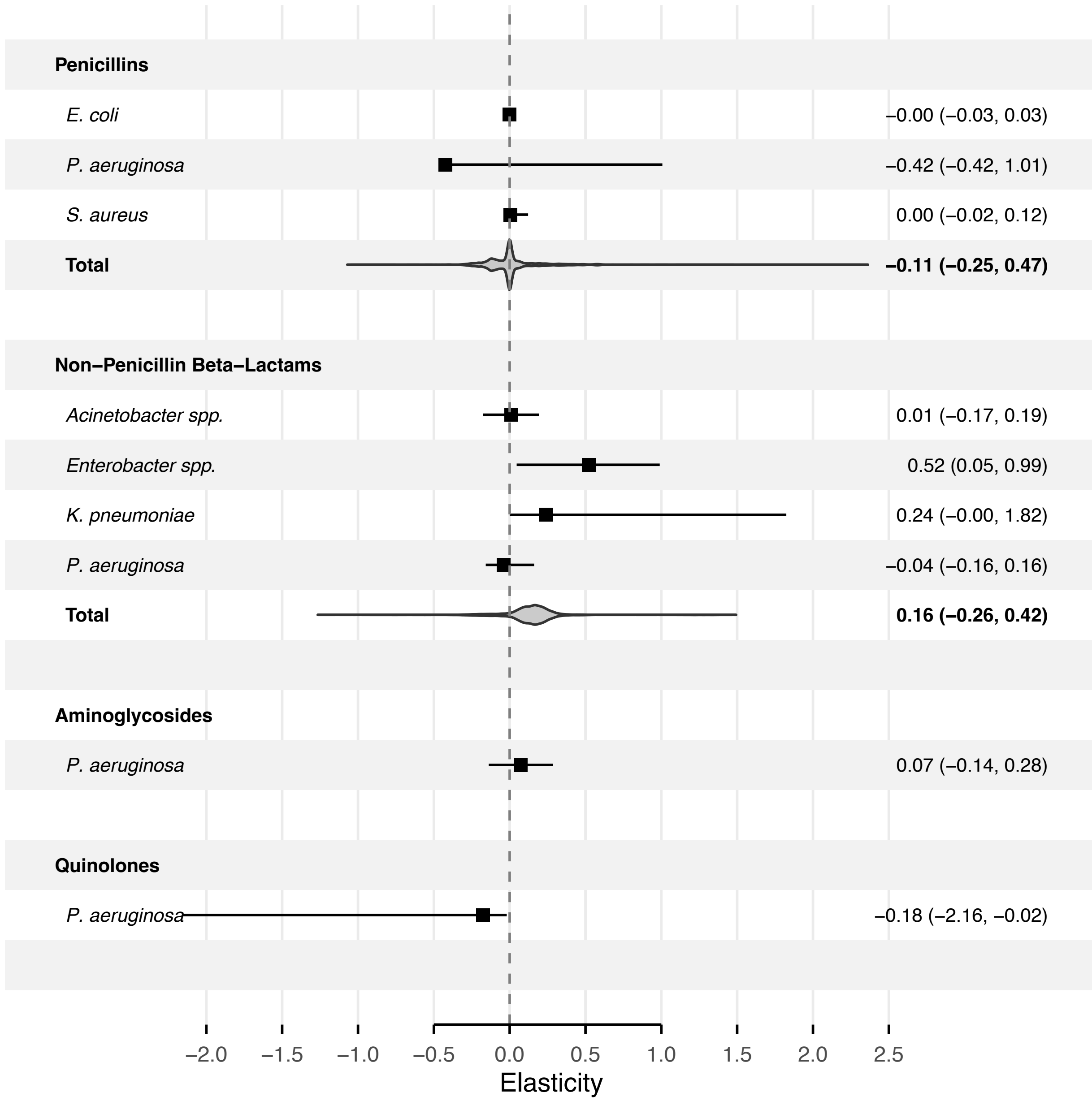

S17 Figure: Pathogen- and class-specific elasticity, calculated using within-hospital measures of antibiotic use and prevalence of AMR among infections observed in the same facilities. Elasticities (and 95% confidence intervals, reported in the right-hand column) are calculated using aggregated data from published studies reporting AMR prevalence in bacterial infections alongside measures of in-hospital antibiotic consumption; we list included studies in **S3 Table**.
