# Supplementary material for: Burden of bacterial antimicrobial resistance associated with modifiable human antibiotic consumption: An observational study
Source: PLoS Med. 2026 Oct 26;23(10):e1005140. doi: 10.1371/journal.pmed.1005140 (PMC13719055; doi:10.1371/journal.pmed.1005140)
Supplement: Supplementary file 20 [file pmed.1005140.s038.pdf]

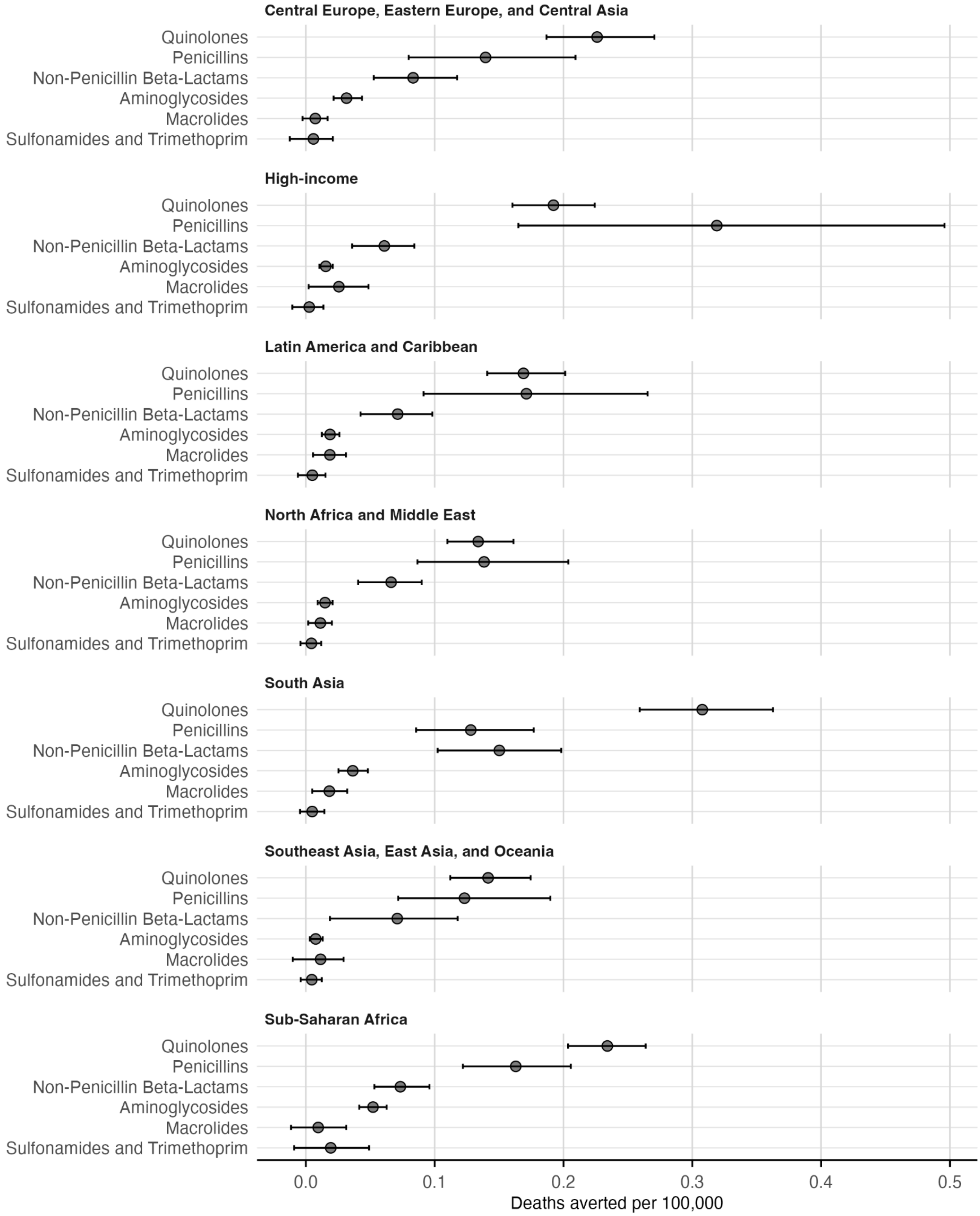

S18 Figure: Counterfactual burden of AMR-attributable mortality, by region. We illustrate estimates (with 95% confidence intervals) for differences between current and expected AMR-attributable deaths with 10% lower antibiotic use, stratified by antibiotic class and region. Estimates project differences based on estimated elasticity in the pathogen and antibiotic class-specific relationship between AMR and consumption (**Figure 1**).
