# Supplementary material for: Burden of bacterial antimicrobial resistance associated with modifiable human antibiotic consumption: An observational study
Source: PLoS Med. 2026 Oct 26;23(10):e1005140. doi: 10.1371/journal.pmed.1005140 (PMC13719055; doi:10.1371/journal.pmed.1005140)
Supplement: Supplementary file 22 [file pmed.1005140.s040.pdf]

S1 Text: List of inclusions and aliases in antibiotic classes.

Tetracyclines: Minocycline, Tigecycline, Glycylcyclines, Doxycycline, Demeclocycline, Methacycline, Omadacycline, Eravacycline, Sarecycline

Penicillins: Mecillinam, Broad spectrum penicillins, Narrow spectrum penicillins, Aminopenicillin, Methicillin, Amoxy/clav, Ampicillin, Oxacillin, Ampicillin sulbactam, Pip/taz

Non-Penicillin Beta-Lactams: Cephalosporins, Cephalosporin, Third-generation cephalosporins, Fourth-generation cephalosporins, Carbapenems, Imipenem, Meropenem, Ceftriaxone, Cefotaxime, Ceftazidime, Cefixime, Doripenem, Ceftazidime avibactam, Cefepime, Aztreonam, Ceftolozane tazobactam, Meropenem vaborbactam, Cefpodoxime, Ceftaroline, Ceftibuten, Ertapenem, Monobactams

Sulfonamides and Trimethoprim: Sulfamethoxaole, Trimethoprim-Sulfamethoxazole, Trimethoprim sulfa

Macrolides: Azithromycin, Clarithromycin, Erythromycin, Telithromycin, Clindamycin, Quinupristin dalfopristin

Aminoglycosides: Gentamicin, Amikacin, Tobramycin, Neomycin, Streptomycin

Quinolones: Fluoroquinolones, Nalidixic Acid, Ciprofloxacin, Levofloxacin, Ofloxacin, Moxifloxacin
