# Supplementary material for: Burden of bacterial antimicrobial resistance associated with modifiable human antibiotic consumption: An observational study
Source: PLoS Med. 2026 Oct 26;23(10):e1005140. doi: 10.1371/journal.pmed.1005140 (PMC13719055; doi:10.1371/journal.pmed.1005140)
Supplement: Supplementary file 23 [file pmed.1005140.s041.pdf]

S2 Text: List of countries (identified by ISO3 codes) included in the study, by pathogen.

Acinetobacter spp.: ARE, ARG, AUT, BEL, BGR, BIH, BLR, CHE, CYP, CZE, DEU, DNK, ESP, FRA, GBR, GRC, HRV, HUN, IND, IRL, ITA, JPN, KOR, LTU, LVA, MYS, NLD, OMN, PAK, PHL, POL, PRT, ROU, RUS, SAU, SRB, SVK, SWE, THA, TUN, TUR, ZAF, AUS, BRA, CAN, CHL, CHN, COL, CRI, DOM, GTM, ISR, JOR, KWT, MEX, MAR, NGA, PAN, SGP, TWN, UKR, USA, VEN, EGY, SLV, FIN, HND, IDN, JAM, KEN, MUS, NAM, NIC, NOR, PRI, SVN, VNM

E. coli: ARE, ARG, AUT, BEL, BGD, BGR, BIH, BLR, CHE, CYP, CZE, DEU, DNK, ESP, EST, FIN, FRA, GBR, GEO, GRC, HRV, HUN, IND, IRL, ISL, ITA, JOR, JPN, KOR, LAO, LBN, LTU, LUX, LVA, MKD, MLT, MYS, NLD, NOR, NPL, OMN, PAK, PHL, POL, PRT, ROU, RUS, SAU, SDN, SRB, SVK, SVN, SWE, THA, TUN, TUR, AUS, BRA, CAN, CHL, CHN, COL, CRI, DOM, EGY, SLV, GTM, HND, IDN, ISR, JAM, KEN, KWT, MUS, MEX, MAR, NAM, NIC, NGA, PAN, PRI, SGP, ZAF, TWN, UKR, USA, VEN, VNM

E. faecalis: AUT, BEL, BGR, BLR, CHE, CYP, CZE, DEU, DNK, ESP, EST, FIN, FRA, GBR, GRC, HRV, HUN, IRL, ITA, LTU, LUX, LVA, NLD, NOR, POL, PRT, ROU, SRB, SVK, SVN, SWE, TUR, ARG, AUS, BRA, CAN, CHL, CHN, COL, CRI, DOM, EGY, SLV, GTM, HND, IND, ISR, JAM, JPN, JOR, KEN, KOR, KWT, MYS, MUS, MEX, MAR, NAM, NIC, NGA, OMN, PAK, PAN, PHL, PRI, RUS, SAU, SGP, ZAF, TWN, THA, TUN, UKR, USA, VEN, VNM

E. faecium: AUT, BEL, CZE, DEU, DNK, ESP, FIN, FRA, GBR, GRC, HRV, HUN, IRL, ITA, NLD, NOR, POL, PRT, SVK, SVN, SWE, ARG, AUS, BRA, BGR, CAN, CHL, CHN, COL, CRI, DOM, EGY, SLV, GTM, HND, IND, ISR, JPN, JOR, KEN, KOR, KWT, LVA, LTU, MYS, MUS, MEX, MAR, NGA, OMN, PAK, PAN, PHL, PRI, ROU, RUS, SAU, SRB, SGP, ZAF, CHE, TWN, THA, TUN, TUR, UKR, USA, VEN, VNM

Enterococcus spp.: ARG, BGR, CAN, HRV, GRC, GTM, IND, ISR, ITA, LBN, PHL, ZAF, USA, AUS, AUT, BEL, BRA, CHL, CHN, COL, CZE, DNK, EGY, SLV, FIN, FRA, DEU, HND, HUN, IRL, JAM, JPN, KOR, LVA, LTU, MYS, MEX, MAR, NLD, PAK, PAN, POL, PRT, ROU, SGP, SVK, SVN, ESP, SWE, CHE, TWN, THA, TUN, TUR, GBR, VEN, SAU

H. influenzae: ARG, AUS, AUT, BEL, BRA, BGR, CAN, CHL, CHN, COL, CRI, HRV, CZE, DNK, DOM, FIN, FRA, DEU, GRC, GTM, HND, HUN, IND, IRL, ISR, ITA, JAM, JPN, JOR, KEN, KOR, KWT, LVA, LBN, LTU, MYS, MUS, MEX, MAR, NLD, NOR, OMN, PAK, PAN, PHL, POL, PRT, ROU, RUS, SAU, SRB, SGP, SVK, SVN, ZAF, ESP, SWE, CHE, TWN, THA, TUN, TUR, UKR, GBR, USA, VEN, VNM

K. pneumoniae: ARE, ARG, AUT, BEL, BGR, BHR, BIH, BLR, CHE, CYP, CZE, DEU, DNK, EGY, ESP, EST, FIN, FRA, GBR, GEO, GRC, HRV, HUN, IND, IRL, ITA, JPN, KOR, LBN, LTU, LUX, LVA, MKD, MLT, MYS, NLD, NOR, NPL, OMN, PAK, PHL, POL, PRT, ROU, RUS, SAU, SRB, SVK, SVN, SWE, THA, TUN, TUR, AUS, BRA, CHL, CHN, COL, ISR, KEN, KWT, MEX, NGA, ZAF, TWN, USA, VEN, CAN, CRI, DOM, SLV, GTM, HND, IDN, JAM, JOR, MUS, MAR, NAM, NIC, PAN, PRI, SGP, UKR, VNM

Morganella spp.: ARG, AUS, AUT, BEL, BRA, CAN, CHL, CHN, COL, CRI, HRV, CZE, DNK, DOM, FIN, FRA, DEU, GRC, GTM, HUN, IND, IRL, ISR, ITA, JPN, JOR, KEN, KOR, KWT, LVA, LTU, MYS, MEX, MAR, NLD, NGA, PAN, PHL, POL, PRT, ROU, RUS, SAU, SGP, ZAF, ESP, SWE, CHE, TWN, THA, TUR, UKR, GBR, USA, VEN

N. gonorrhoeae: CHE, FIN, JPN, MYS, NOR, PHL, THA, UGA, ZAF, ARG, AUS, BEL, CHL, DNK, FRA, DEU, HUN, ISR, ITA, KEN, KWT, PRT, TWN, USA, VEN, ARE, AUT, BLR, BRA, BRN, BTN, CAN, CHN, CIV, COL, CUB, CYP, CZE, ECU, ESP, EST, GBR, GRC, HRV, IDN, IND, IRL, ISL, KGZ, KHM, KOR, LKA, LUX, LVA, MDG, MLT, MMR, MNG, MWI, NLD, NZL, PAK, PER, POL, ROU, RUS, SAU, SGP, SVK, SVN, SWE, UKR, URY, VNM, DOM, MUS, PAN, PRY, SLV, TUN, ZWE, BHR, BOL, FJI, LAO, MAR, MOZ, OMN, PNG, TON, GHA, TZA

P. aeruginosa: AUT, BEL, BGR, CHE, CYP, CZE, DEU, DNK, ESP, EST, FIN, FRA, GBR, GRC, HRV, HUN, IRL, ITA, LTU, LUX, NLD, NOR, POL, PRT, ROU, SRB, SVK, SVN, SWE, TUR, ARG, AUS, BRA, CAN, CHL, CHN, COL, CRI, DOM, EGY, SLV, GTM, HND, IND, IDN, ISR, JAM, JPN, JOR, KEN, KOR, KWT, LVA, LBN, MYS, MUS, MEX, MAR, NAM, NIC, NGA, OMN, PAK, PAN, PHL, PRI, RUS, SAU, SGP, ZAF, TWN, THA, TUN, UKR, USA, VEN, VNM

S. agalactiae: ARG, BRA, CHL, COL, GTM, MEX, PAN, VEN, AUS, AUT, BEL, CAN, CHN, CRI, HRV, CZE, DNK, DOM, FIN, FRA, DEU, GRC, HUN, IND, IRL, ISR, ITA, JPN, JOR, KEN, KOR, KWT, LVA, LTU, MYS, MAR, NLD, PHL, POL, PRT, ROU, RUS, SAU, SGP, ZAF, ESP, SWE, CHE, TWN, THA, TUR, UKR, GBR, USA, BGR, EGY, HND, JAM, LBN, MUS, NAM, NOR, OMN, PAK, PRI, SRB, SVK, SVN, TUN, VNM

S. aureus: AUT, BEL, BGR, BIH, BLR, CHE, CYP, CZE, DEU, DNK, ESP, EST, FIN, FRA, GBR, GRC, HRV, HUN, IRL, ISL, ITA, LTU, LUX, LVA, MLT, NLD, NOR, POL, PRT, ROU, RUS, SRB, SVK, SVN, SWE, TUR, ARG, AUS, BRA, CAN, CHL, CHN, COL, CRI, DOM, GTM, IND, ISR, JPN, JOR, KEN, KOR, KWT, MYS, MEX, MAR, NGA, PAN, PHL, SAU, SGP, ZAF, TWN, THA, UKR, USA, VEN, EGY, SLV, HND, JAM, LBN, MUS, NAM, NIC, OMN, PAK, PRI, TUN, VNM

S. pneumoniae: ARE, ARG, AUT, BEL, CHE, CZE, DEU, DNK, ESP, EST, FIN, FRA, GBR, HRV, HUN, IRL, ITA, JPN, KOR, LTU, LVA, MLT, MYS, NLD, NOR, PHL, POL, PRT, ROU, SVN, SWE, TUR, ZAF, AUS, BRA, CHL, CHN, COL, GRC, ISR, KEN, KWT, MEX, RUS, TWN, THA, USA, VEN, CAN, IND, JAM, PAK, SGP, BGR, CRI, DOM, GTM, HND, JOR, MUS, MAR, OMN, PAN, SAU, SRB, SVK, TUN, UKR, VNM

S. pyogenes: ARG, AUS, AUT, BEL, BRA, CAN, CHL, CHN, COL, CRI, HRV, CZE, DNK, DOM, FIN, FRA, DEU, GRC, GTM, HUN, IND, IRL, ISR, ITA, JPN, KEN, KOR, KWT, LVA, LTU, MYS, MEX, MAR, NLD, PAN, PHL, POL, PRT, ROU, RUS, SGP, ZAF, ESP, SWE, CHE, TWN, THA, TUR, UKR, GBR, USA, VEN, JOR, PAK

Salmonella spp.: ARE, ARG, BHR, CHE, FIN, IND, JPN, KOR, MLT, MYS, NPL, PAK, PHL, POL, SAU, THA, ZAF, JOR
