# Supplementary material for: Burden of bacterial antimicrobial resistance associated with modifiable human antibiotic consumption: An observational study
Source: PLoS Med. 2026 Oct 26;23(10):e1005140. doi: 10.1371/journal.pmed.1005140 (PMC13719055; doi:10.1371/journal.pmed.1005140)
Supplement: Supplementary file 24 [file pmed.1005140.s042.pdf]

S3 Text: List of countries (identified by ISO3 codes) included in the study using raw IQVIA consumption data, by pathogen.

Acinetobacter spp.: ARE, ARG, AUT, BEL, BGR, BLR, CHE, CZE, DEU, DNK, ESP, FRA, GBR, GRC, HRV, HUN, IND, IRL, ITA, JPN, KOR, LVA, MYS, NLD, PAK, PHL, POL, PRT, ROU, RUS, SAU, SRB, SVK, SWE, THA, TUN, TUR, ZAF, AUS, BRA, CAN, CHL, CHN, COL, JOR, KWT, MEX, MAR, TWN, USA, EGY, FIN, IDN, LTU, NOR, PRI, SGP, SVN, VEN, VNM

E. coli: ARE, ARG, AUT, BEL, BGR, BLR, CHE, CZE, DEU, DNK, ESP, EST, FIN, FRA, GBR, GRC, HRV, HUN, IND, IRL, ITA, JOR, JPN, KOR, LBN, LTU, LUX, LVA, MYS, NLD, NOR, PAK, PHL, POL, PRT, ROU, RUS, SAU, SRB, SVK, SVN, SWE, THA, TUN, TUR, AUS, BRA, CAN, CHL, CHN, COL, EGY, IDN, KWT, MEX, MAR, PRI, SGP, ZAF, TWN, USA, VEN, VNM

E. faecalis: AUT, BEL, BGR, BLR, CHE, CZE, DEU, DNK, ESP, FIN, FRA, GBR, GRC, HRV, HUN, IRL, ITA, LUX, LVA, NLD, NOR, POL, PRT, ROU, SRB, SVK, SVN, SWE, TUR, AUS, JOR, KWT, SAU, ARG, BRA, CAN, CHL, CHN, COL, EGY, IND, JPN, KOR, LTU, MYS, MEX, MAR, PAK, PHL, PRI, RUS, SGP, ZAF, TWN, THA, TUN, USA, VEN, VNM

E. faecium: AUT, BEL, CZE, DEU, DNK, ESP, FIN, FRA, GBR, GRC, HRV, HUN, IRL, ITA, NLD, NOR, POL, PRT, SVK, SVN, SWE, AUS, JOR, KWT, SAU, SRB, ARG, BRA, BGR, CAN, CHL, CHN, COL, EGY, IND, JPN, KOR, LVA, LTU, MYS, MEX, MAR, PAK, PHL, PRI, ROU, RUS, SGP, ZAF, CHE, TWN, THA, TUN, TUR, USA, VEN, VNM

Enterococcus spp.: ARG, BGR, CAN, HRV, GRC, IND, ITA, LBN, PHL, ZAF, USA, AUT, DNK, FIN, DEU, HUN, IRL, NLD, ESP, SAU, AUS, BEL, BRA, CHL, CHN, COL, CZE, EGY, FRA, JPN, KOR, LVA, LTU, MYS, MEX, MAR, PAK, POL, PRT, ROU, SGP, SVK, SVN, SWE, CHE, TWN, THA, TUN, TUR, GBR, VEN

H. influenzae: ARG, AUS, AUT, BEL, BRA, BGR, CAN, CHL, CHN, COL, HRV, CZE, DNK, FIN, FRA, DEU, GRC, HUN, IND, IRL, ITA, JPN, JOR, KOR, KWT, LVA, LBN, LTU, MYS, MEX, MAR, NLD, NOR, PAK, PHL, POL, PRT, ROU, RUS, SAU, SRB, SGP, SVK, SVN, ZAF, ESP, SWE, CHE, TWN, THA, TUN, TUR, GBR, USA, VEN, VNM

K. pneumoniae: ARE, ARG, AUT, BEL, BGR, BLR, CHE, CZE, DEU, DNK, EGY, ESP, EST, FIN, FRA, GBR, GRC, HRV, HUN, IND, IRL, ITA, JPN, KOR, LBN, LTU, LUX, LVA, MYS, NLD, NOR, PAK, PHL, POL, PRT, ROU, RUS, SAU, SRB, SVK, SVN, SWE, THA, TUN, TUR, AUS, BRA, CHL, CHN, COL, KWT, MEX, ZAF, TWN, USA, VEN, CAN, IDN, JOR, MAR, PRI, SGP, VNM

Morganella spp.: ARG, AUS, AUT, BEL, BRA, CAN, CHL, CHN, COL, HRV, CZE, DNK, FIN, FRA, DEU, GRC, HUN, IND, IRL, ITA, JPN, JOR, KOR, KWT, LVA, MYS, MEX, MAR, NLD, PHL, POL, PRT, ROU, RUS, SAU, ZAF, ESP, SWE, CHE, TWN, THA, TUR, GBR, USA, VEN

N. gonorrhoeae: CHE, FIN, JPN, MYS, NOR, PHL, THA, ZAF, ARG, AUS, BEL, CHL, DNK, FRA, DEU, HUN, ITA, KWT, PRT, TWN, USA, VEN, ARE, ESP, HRV, IRL, NLD, NZL, SAU, SVN, SWE, AUT, BLR, BRA, CAN, COL, CZE, ECU, EST, GBR, GRC, IDN, IND, LUX, LVA, PAK, PER, POL, ROU, RUS, SVK, TUN, UKR, URY, CHN, DOM, KOR, LKA, MAR, SGP, VNM

P. aeruginosa: AUT, BEL, BGR, CHE, CZE, DEU, DNK, ESP, FIN, FRA, GBR, GRC, HRV, HUN, IRL, ITA, LUX, NLD, NOR, POL, PRT, ROU, SRB, SVK, SVN, SWE, TUR, ARG, AUS, BRA, CAN, CHL, CHN, COL, EGY, IND, IDN, JPN, JOR, KOR, KWT, LVA, LBN, LTU, MYS, MEX, MAR, PAK, PHL, PRI, RUS, SAU, SGP, ZAF, TWN, THA, TUN, USA, VEN, VNM

S. agalactiae: AUS, HRV, DNK, FIN, DEU, HUN, IRL, JOR, KWT, NLD, SAU, ESP, SWE, AUT, SRB

S. aureus: AUT, BEL, BGR, BLR, CHE, CZE, DEU, DNK, ESP, EST, FIN, FRA, GBR, GRC, HRV, HUN, IRL, ITA, LTU, LUX, LVA, NLD, NOR, POL, PRT, ROU, RUS, SRB, SVK, SVN, SWE, TUR, AUS, JOR, KWT, SAU, ARG, BRA, CAN, CHL, CHN, COL, IND, JPN, KOR, MYS, MEX, MAR, PHL, ZAF, TWN, THA, USA, VEN

S. pneumoniae: ARE, ARG, AUT, BEL, CHE, CZE, DEU, DNK, ESP, FIN, FRA, GBR, HRV, HUN, IRL, ITA, JPN, KOR, LVA, MYS, NLD, NOR, PHL, POL, PRT, ROU, SVN, SWE, TUR, ZAF, AUS, BRA, CHL, CHN, COL, GRC, KWT, MEX, RUS, TWN, THA, USA, VEN, CAN, IND, PAK, SGP, BGR, JOR, LTU, MAR, SAU, SRB, SVK, TUN, VNM

S. pyogenes: AUS, HRV, DNK, FIN, DEU, HUN, IRL, KWT, NLD, ESP, SWE, JOR

Salmonella spp.: ARE, ARG, CHE, FIN, IND, JPN, KOR, MYS, PAK, PHL, POL, SAU, THA, ZAF, JOR
