# Supplementary material for: Burden of bacterial antimicrobial resistance associated with modifiable human antibiotic consumption: An observational study
Source: PLoS Med. 2026 Oct 26;23(10):e1005140. doi: 10.1371/journal.pmed.1005140 (PMC13719055; doi:10.1371/journal.pmed.1005140)
Supplement: Supplementary file 25 [file pmed.1005140.s043.pdf]

S5 Text: Models accessed via GitHub copilot to support statistical programming.

Claude Haiku 4.5
Claude Haiku 5.5
Claude Opus 4.8
Claude Opus 4.8 (fast mode) (preview)
Claude Opus 5
Claude Opus 5.5
Claude Fable 5
Claude Fable 5.1
Claude Sonnet 4.6
Claude Sonnet 5
Claude Sonnet 5.5
Gemini 3.7 Flash
Gemini 3.8 Flash
Gemini 4 Argon
MAI-Code-1.1-Flash
GPT-5 mini
GPT-5.3-Codex
GPT-5.4
GPT-5.4 mini
GPT-5.4 nano1
GPT-5.5
GPT-5.6 Luna
GPT-5.6 Sol
GPT-5.6 Terra
GPT-6 Astra
GPT-6 Luna
GPT-6 Sol
Grok 4.5
Grok 4.6
Grok 4.7
Kimi K3
