# Supplementary material for: Burden of bacterial antimicrobial resistance associated with modifiable human antibiotic consumption: An observational study
Source: PLoS Med. 2026 Oct 26;23(10):e1005140. doi: 10.1371/journal.pmed.1005140 (PMC13719055; doi:10.1371/journal.pmed.1005140)
Supplement: Supplementary file 26 [file pmed.1005140.s003.docx]

**S1 Table: Included data for estimation of elasticity by pathogen and drug class.**

| **Pathogen** | **Antibiotic class** | | | | | | | |
| --- | --- | --- | --- | --- | --- | --- | --- | --- |
|  | Tetracyclines | Penicillins | Non-Penicillin Beta-Lactams | Sulfonamides and Trimethroprim | Macrolides | Aminoglycosides | Quinolones | **Total** |
| *Acinetobacter* spp. | 28,645 (525) | 29,570 (683) | 201,090 (2,996) | 1,887 (51) | — | 107,873 (835) | 79,780 (793) | **448,845** |
| *E. coli* | 138,869 (1,141) | 265,514 (1,964) | 12,263,316 (4,697) | 649,184 (85) | 5 (5) | 1,580,625 (1,180) | 5,437,736 (1,218) | **20,335,244** |
| *E. faecalis* | 49,811 (1,149) | 46,448 (1,094) | — | — | 7,617 (237) | 166,140 (301) | 25,971 (601) | **295,987** |
| *E. faecium* | 24,718 (1,088) | 22,040 (1,031) | — | — | 5,376 (230) | 66,076 (201) | 12,875 (570) | **131,085** |
| *Enterococcus* spp. | 2,233 (583) | 2,180 (558) | — | — | 85 (57) | — | 1,117 (292) | **5,530** |
| *H. influenzae* | 53,567 (1,124) | 87,124 (2,003) | 105,892 (2,845)* | — | 1,544 (71)* | — | 27,483 (587)* | **140,691** |
| *K. pneumoniae* | 109,502 (1,133) | 249,058 (2,003) | 2,853,357 (4,447) | 121,919 (85) | — | 404,687 (1,007) | 957,408 (1,043) | **4,695,931** |
| *Morganella* spp. | 2,899 (264) | 9,379 (845) | 24,348 (2,229) | 682 (53) | — | 3,581 (317) | 3,581 (317) | **44,470** |
| *P. aeruginosa* | — | 201,608 (897) | 670,828 (3,556) | — | — | 252,632 (920) | 258,444 (973) | **1,383,512** |
| *Proteus* spp. | 4 (3) | 16 (12) | 28 (21) | 4 (3) | — | 8 (6) | 8 (6) | **—** |
| *S. algalactiae* | 42,464 (1,157) | 40,115 (1,076)* | 46,971 (1,716)* | — | 6,577 (544) | — | 21,629 (604) | **70,670** |
| *S. aureus* | 233,066 (1,225) | 883,624 (762) | 68,020 (266) | 47,564 (193) | 137,027 (558) | 47,564 (193) | 181,241 (852) | **1,598,106** |
| *S. pneumoniae* | 38,131 (608)* | 295,035 (1,444) | 125,740 (1,835) | 3,110 (8) | 321,157 (2,400) | — | 51,887 (817) | **793,819** |
| *S. pyogenes* | 19,422 (500) | 11,966 (330)* | 38,096 (1,085)* | — | 16,500 (518) | — | 10,108 (275) | **46,030** |
| *Salmonella* spp. | — | 1 (1) | 42,415 (73) | 1 (1) | — | — | 21,704 (30) | **64,119** |
| *Shigella* spp. | — | — | 6,353 (10) | — | 1,900 (2) | — | 6,965 (6) | **—** |
| **Total** | **705,196** | **2,091,580** | **16,601,800** | **821,236** | **691,004** | **2,629,178** | **7,289,251** | **30,829,245** |

Values in the table indicate total numbers of tested isolates, and (in brackets) independent observations (ie, country-year-database combinations) for each combination of bacteria type and antibiotic class. Grey cells indicate exclusions—ie, bacteria-antibiotic combinations for which there are 10 or fewer independent observations, or fewer than 100 total tested isolates, or for which there are more than 10 independent observations but no data recording resistance prevalence (marked by asterisks).
