# Supplementary material for: Burden of bacterial antimicrobial resistance associated with modifiable human antibiotic consumption: An observational study
Source: PLoS Med. 2026 Oct 26;23(10):e1005140. doi: 10.1371/journal.pmed.1005140 (PMC13719055; doi:10.1371/journal.pmed.1005140)
Supplement: Supplementary file 27 [file pmed.1005140.s004.docx]

**S2 Table: Results of permutation tests for class specificity in the relationship between antimicrobial resistance prevalence and antibiotic consumption.**

| **Pathogen** | **Antibiotic class** | | | | | | |
| --- | --- | --- | --- | --- | --- | --- | --- |
|  | Tetracyclines | Penicillins | Non-Penicillin Beta-Lactams | Sulfonamides and Trimethroprim | Macrolides | Aminoglycosides | Quinolones |
| *Acinetobacter* spp. | **1** | **6.2×10-218** | **6.8×10-31** | **1** | — | **1** | **0** |
| *E. coli* | *1* | **1** | **0** | **1** | — | **0** | **0** |
| *E. faecalis* | *1* | *1* | — | — | **9.1×10-141** | **1** | **0** |
| *E. faecium* | **0** | **1** | — | — | **1** | **0** | **1** |
| *Enterococcus* spp. | **1** | *1* | — | — | — | — | **0** |
| *H. influenzae* | **1** | **0** | — | — | — | — | — |
| *K. pneumoniae* | *1* | **1** | **7.8×10-70** | **1** | — | **0** | **0** |
| *Morganella* spp. | **1** | **1** | **1** | *1* | — | **0** | **0** |
| *P. aeruginosa* | — | **1** | **1.0×10-147** | — | — | **1** | **0** |
| *S. agalactiae* | **1** | — | — | — | *1* | — | **0** |
| *S. aureus* | **1** | **0** | *1* | *1* | **7.0×10-91** | **1** | **0** |
| *S. pneumoniae* | — | **1** | **1** | — | **0** | — | **1** |
| *S. pyogenes* | *0.79* | — | — | — | 1 | — | **0** |
| *Salmonella* spp. | — | — | *1* | — | — | — | **0** |

Values in the table are Wilcoxon signed-rank test *p*-values for the null hypothesis that the bootstrapped elasticities for each drug-pathogen combination are from the same distribution as the bootstrapped elasticities for resistance to one drug with respect to consumption of others. These values indicate drug class specificity in the relationship between AMR and antibiotic consumption, with low values supporting rejection of the null hypothesis that class-specific associations between AMR and consumption are drawn from the same distribution as associations across classes. Bolded entries have positive elasticities in the main analysis; those in grey-shaded boxes have positive elasticities in the main analysis with 95% confidence intervals excluding 0. Table entries of “0” indicate a very small *p*-value below machine tolerance.
