# Supplementary material for: Burden of bacterial antimicrobial resistance associated with modifiable human antibiotic consumption: An observational study
Source: PLoS Med. 2026 Oct 26;23(10):e1005140. doi: 10.1371/journal.pmed.1005140 (PMC13719055; doi:10.1371/journal.pmed.1005140)
Supplement: Supplementary file 28 [file pmed.1005140.s005.docx]

**S3 Table: Studies assessing the relationship between within-hospital antimicrobial consumption and resistance prevalence.**

| **Authors** | **Study dates** | **Country** | **Pathogens** | **Antibiotic classes** |
| --- | --- | --- | --- | --- |
| Xu J, Duan X, Wu H, and Zhou Q [1] | 2003–2011 | China | *P. aeruginosa* | J01D, J01G, J01M |
| Kim B, Kim Y, Hwang H, et al. [2] | 2004–2012 | South Korea | *K. pneumoniae, P. aeruginosa* | J01D |
| Huh K, Chung DR, Park HJ, et al. [3] | 2005–2010 | South Korea | *E. coli, S. aureus, P. aeruginosa* | J01C, J01D |
| Federico MP and Furtado GH [4] | 2007–2016 | Brazil | *Acinetobacter* spp.*, K. pneumoniae, P. aeruginosa* | J01D |
| Paiboonvong T, Tedtaisong P, Montakantikul P, et al. [5] | 2007–2013 | Thailand | *Acinetobacter* spp.*, P. aeruginosa* | J01D |
| Kato I, Fujimoto F, Higurashi Y, et al. [6] | 2009–2012 | Japan | *P. aeruginosa* | J01C, J01D, J01G, J01M |
| Zhang D, Cui K, Wang T, et al. [7] | 2010–2016 | China | *S. aureus* | J01C |
| El-Basst R, Saliba S, Saleh L, et al. [8] | 2010–2018 | Lebanon | *P. aeruginosa* | J01C, J01D, J01G, J01M |
| Liu L, Liu B, Li Y, and Zhang W [9] | 2012–2017 | China | *P. aeruginosa* | J01C, J01D, J01G, J01M |
| Baditoiu L, Axente C, Lungeanu D, et al. [10] | 2012–2013 | Romania | *Acinetobacter* spp.*, S. aureus, P. aeruginosa* | J01C, J01D |
| Yang P, Chen Y, Jiang S, et al. [11] | 2014 | China | *E. coli* | J01M |
| Pérez-Lazo G, Abarca-Salazar S, Lovón R, et al. [12] | 2015–2018 | Peru | *K. pneumoniae, Acinetobacter* spp*., Enterobacter* spp*., S. aureus, P. aeruginosa* | J01C, J01D, J01G, J01M |
| Sid Ahmed MA, Abdel Hadi H, Abu Jarir S, et al. [13] | 2015–2017 | Qatar | *P. aeruginosa* | J01C, J01D, J01G, J01M |
| Gong W, Tang W, Luo L, et al. [14] | 2016–2021 | China | *Acinetobacter* spp. | J01D |

Antibiotic classes are abbreviated by their ATC code. J01C: Penicillins, J01D: Non-Penicillin Beta-Lactams, J01G: Aminoglycosides, J01M: Quinolones.

**References**

1. Xu J, Duan X, Wu H, Zhou Q. Surveillance and correlation of antimicrobial usage and resistance of *Pseudomonas aeruginosa*: a hospital population-based study. PLOS ONE. 2013;8: e78604. doi:10.1371/journal.pone.0078604
2. Kim B, Kim Y, Hwang H, Kim J, Kim S-W, Bae I-G, et al. Trends and correlation between antibiotic usage and resistance pattern among hospitalized patients at university hospitals in Korea, 2004 to 2012: A nationwide multicenter study. Medicine. 2018;97: e13719. doi:10.1097/MD.0000000000013719
3. Huh K, Chung DR, Park HJ, Kim M-J, Lee NY, Ha YE, et al. Impact of monitoring surgical prophylactic antibiotics and a computerized decision support system on antimicrobial use and antimicrobial resistance. Am J Infect Control. 2016;44: e145–e152. doi:10.1016/j.ajic.2016.01.025
4. Federico MP, Furtado GH. Immediate and later impacts of antimicrobial consumption on carbapenem-resistant *Acinetobacter* spp., *Pseudomonas aeruginosa*, and *Klebsiella* spp. in a teaching hospital in Brazil: a 10-year trend study. Eur J Clin Microbiol Infect Dis. 2018;37: 2153–2158. doi:10.1007/s10096-018-3352-1
5. Paiboonvong T, Tedtaisong P, Montakantikul P, Gorsanan S, Tantisiriwat W. Correlation between carbapenem consumption and carbapenems susceptibility profiles of *Acinetobacter baumannii* and *Pseudomonas aeruginosa* in an academic medical center in Thailand. Antibiotics. 2022;11: 143. doi:10.3390/antibiotics11020143
6. Kato I, Fujimoto F, Higurashi Y, Yamaguchi R, Takayama K, Suzuki M, et al. Antibiotic susceptibilities of *Pseudomonas aeruginosa* isolated from blood samples and antibiotic utilization in a university hospital in Japan. Infect Dis Ther. 2015;4: 213–218. doi:10.1007/s40121-015-0066-x
7. Zhang D, Cui K, Wang T, Dong H, Feng W, Ma C, et al. Trends in and correlations between antibiotic consumption and resistance of *Staphylococcus aureus* at a tertiary hospital in China before and after introduction of an antimicrobial stewardship programme. Epidemiology & Infection. 2019;147: e48. doi:10.1017/S0950268818003059
8. El-Basst R, Saliba S, Saleh L, Saoud N, Azar E, Zalloua P, et al. The effect of decreased antipseudomonal drug consumption on *Pseudomonas aeruginosa* incidence and antimicrobial susceptibility profiles over 9 years in a Lebanese tertiary care center. Antibiotics. 2023;12: 192. doi:10.3390/antibiotics12020192
9. Liu L, Liu B, Li Y, Zhang W. Successful control of resistance in *Pseudomonas aeruginosa* using antibiotic stewardship and infection control programs at a Chinese university hospital: a 6-year prospective study. Infect Dis Rep. 2018;11: 637–646. doi:10.2147/IDR.S163853
10. Baditoiu L, Axente C, Lungeanu D, Muntean D, Horhat F, Moldovan R, et al. Intensive care antibiotic consumption and resistance patterns: a cross-correlation analysis. Ann Clin Microbiol Antimicrob. 2017;16: 71. doi:10.1186/s12941-017-0251-8
11. Yang P, Chen Y, Jiang S, Shen P, Lu X, Xiao Y. Association between the rate of fluoroquinolones-resistant gram- negative bacteria and antibiotic consumption from China based on 145 tertiary hospitals data in 2014. BMC Infect Dis. 2020;20: 269. doi:10.1186/s12879-020-04981-0
12. Pérez-Lazo G, Abarca-Salazar S, Lovón R, Rojas R, Ballena-López J, Morales-Moreno A, et al. Antibiotic consumption and its relationship with bacterial resistance profiles in ESKAPE pathogens in a Peruvian hospital. Antibiotics. 2021;10: 1221. doi:10.3390/antibiotics10101221
13. Sid Ahmed MA, Abdel Hadi H, Abu Jarir S, Al Khal AL, Al-Maslamani MA, Jass J, et al. Impact of an antimicrobial stewardship programme on antimicrobial utilization and the prevalence of MDR *Pseudomonas aeruginosa* in an acute care hospital in Qatar. JAC Antimicrob Resist. 2020;2: dlaa050. doi:10.1093/jacamr/dlaa050
14. Gong W, Tang W, Luo L, Zhang F, Cai C, Zhang J, et al. Trends and correlation between antimicrobial resistance and antibiotics consumption in a specialist children’s hospital from 2016 to 2021. Infect Dis Rep. 2022;15: 5679– 5689. doi:10.2147/IDR.S381604
