# Supplementary material for: Burden of bacterial antimicrobial resistance associated with modifiable human antibiotic consumption: An observational study
Source: PLoS Med. 2026 Oct 26;23(10):e1005140. doi: 10.1371/journal.pmed.1005140 (PMC13719055; doi:10.1371/journal.pmed.1005140)
Supplement: Supplementary file 29 [file pmed.1005140.s006.docx]

**S4 Table: A measure of distortion of AMR due to continuity correction in each pathogen and antibiotic class, ordered by total deaths attributable to AMR.**

| **Pathogen** | **Antibiotic class** | **Continuity factor** | **Attributable burden (deaths)** |
| --- | --- | --- | --- |
| *S. aureus* | Penicillins | 0.860 | 121077 |
| *Acinetobacter* spp. | Non-Penicillin Beta-Lactams | 0.935 | 57729 |
| *E. coli* | Quinolones | 0.941 | 55975 |
| *K. pneumoniae* | Non-Penicillin Beta-Lactams | 0.698 | 55652 |
| *S. pneumoniae* | Non-Penicillin Beta-Lactams | 0.546 | 41922 |
| *Acinetobacter* spp. | Quinolones | 0.949 | 40022 |
| *S. pneumoniae* | Sulfonamides and Trimethoprim | 0.957 | 38728 |
| *P. aeruginosa* | Non-Penicillin Beta-Lactams | 0.925 | 38140 |
| *E. faecium* | Quinolones | 0.986 | 37157 |
| *E. coli* | Sulfonamides and Trimethoprim | 0.963 | 30156 |
| *E. coli* | Non-Penicillin Beta-Lactams | 0.555 | 29493 |
| *K. pneumoniae* | Quinolones | 0.879 | 29023 |
| *E. faecalis* | Quinolones | 0.950 | 26797 |
| *K. pneumoniae* | Aminoglycosides | 0.884 | 26341 |
| *K. pneumoniae* | Sulfonamides and Trimethoprim | 0.950 | 23486 |
| *S. aureus* | Macrolides | 0.979 | 19608 |
| *S. aureus* | Sulfonamides and Trimethoprim | 0.586 | 18670 |
| *P. aeruginosa* | Quinolones | 0.942 | 18311 |
| *S. aureus* | Quinolones | 0.959 | 15905 |
| *S. agalactiae* | Macrolides | 0.877 | 13530 |
| *Acinetobacter* spp. | Penicillins | 0.955 | 13314 |
| *S. pneumoniae* | Macrolides | 0.906 | 12519 |
| *Enterococcus* spp. | Quinolones | 0.688 | 12237 |
| *E. coli* | Aminoglycosides | 0.868 | 11716 |
| *S. agalactiae* | Quinolones | 0.359 | 11504 |
| *S. pneumoniae* | Quinolones | 0.245 | 11175 |
| *E. coli* | Penicillins | 0.986 | 10491 |
| *Acinetobacter* spp. | Aminoglycosides | 0.930 | 10424 |
| *P. aeruginosa* | Penicillins | 0.915 | 10311 |
| *K. pneumoniae* | Penicillins | 0.964 | 7934 |
| *Shigella* spp. | Quinolones | 0.163 | 5989 |
| *Salmonella* spp. | Quinolones | 0.842 | 5618 |
| *H. influenzae* | Penicillins | 0.873 | 4291 |
| *S. pyogenes* | Macrolides | 0.726 | 3633 |
| *P. aeruginosa* | Aminoglycosides | 0.820 | 3005 |
| *Proteus* spp. | Quinolones | 0.741 | 2972 |
| *S. pneumoniae* | Penicillins | 0.597 | 2044 |
| *Proteus* spp. | Sulfonamides and Trimethoprim | 0.490 | 1618 |
| *Proteus* spp. | Penicillins | 0.738 | 1330 |
| *Morganella* spp. | Quinolones | 0.864 | 427 |
| *Morganella* spp. | Non-Penicillin Beta-Lactams | 0.984 | 154 |

The continuity factor is equal to $\mathrm{mean}(\frac{y}{y+0.01})$ where y is the percentage of isolates observed with AMR for each observation in the dataset.
