# Supplementary material for: Burden of bacterial antimicrobial resistance associated with modifiable human antibiotic consumption: An observational study
Source: PLoS Med. 2026 Oct 26;23(10):e1005140. doi: 10.1371/journal.pmed.1005140 (PMC13719055; doi:10.1371/journal.pmed.1005140)
Supplement: Supplementary file 30 [file pmed.1005140.s007.docx]

**S5 Table: Loadings of World Bank Health, Nutrition, and Population Statistics variables for the first principal component.**

| **Variable** | **Loading** |
| --- | --- |
| Immunization, DPT (% of children ages 12-23 months) | -0.10699 |
| Immunization, measles (% of children ages 12-23 months) | -0.10699 |
| Immunization, Pol3 (% of one-year-old children) | -0.10699 |
| Stillbirth rate (per 1,000 total births) | -0.10651 |
| Mortality rate, under-5, male (per 1,000) | -0.10613 |
| Mortality rate, under-5 (per 1,000) | -0.10611 |
| Mortality rate, under-5, female (per 1,000) | -0.10609 |
| Mortality rate, infant, male (per 1,000 live births) | -0.10602 |
| Mortality rate, infant (per 1,000 live births) | -0.106 |
| Mortality rate, infant, female (per 1,000 live births) | -0.10598 |
| Mortality rate, neonatal (per 1,000 live births) | -0.1059 |
| Probability of dying among adolescents ages 10-19 years (per 1,000) | -0.10584 |
| Probability of dying among adolescents ages 10-19 years, female (per 1,000) | -0.10584 |
| Probability of dying among adolescents ages 10-19 years, male (per 1,000) | -0.10584 |
| Probability of dying among youth ages 20-24 years, female (per 1,000) | -0.10584 |
| Probability of dying among children ages 5-9 years (per 1,000) | -0.10583 |
| Probability of dying among children ages 5-9 years, female (per 1,000) | -0.10583 |
| Probability of dying among children ages 5-9 years, male (per 1,000) | -0.10583 |
| Probability of dying among youth ages 20-24 years (per 1,000) | -0.10583 |
| Probability of dying among youth ages 20-24 years, male (per 1,000) | -0.10583 |
| Probability of dying among adolescents ages 15-19 years (per 1,000) | -0.10582 |
| Probability of dying among adolescents ages 15-19 years, female (per 1,000) | -0.10582 |
| Probability of dying among adolescents ages 15-19 years, male (per 1,000) | -0.10582 |
| Probability of dying among adolescents ages 10-14 years (per 1,000) | -0.10581 |
| Probability of dying among adolescents ages 10-14 years, female (per 1,000) | -0.10581 |
| Probability of dying among adolescents ages 10-14 years, male (per 1,000) | -0.10581 |
| Out-of-pocket expenditure (% of current health expenditure) | -0.10035 |
| Domestic private health expenditure (% of current health expenditure) | -0.10033 |
| Domestic general government health expenditure (% of GDP) | -0.10025 |
| Domestic general government health expenditure (% of general government expenditure) | -0.10024 |
| Domestic general government health expenditure (% of current health expenditure) | -0.10014 |
| Total alcohol consumption per capita, female (liters of pure alcohol, projected estimates, female 15+ years of age) | -0.099907 |
| Total alcohol consumption per capita (liters of pure alcohol, projected estimates, 15+ years of age) | -0.099898 |
| Total alcohol consumption per capita, male (liters of pure alcohol, projected estimates, male 15+ years of age) | -0.099889 |
| Current health expenditure (% of GDP) | -0.099495 |
| Out-of-pocket expenditure per capita (current US$) | -0.098561 |
| Out-of-pocket expenditure per capita, PPP (current international $) | -0.097545 |
| Domestic private health expenditure per capita (current US$) | -0.097374 |
| Domestic private health expenditure per capita, PPP (current international $) | -0.097203 |
| Mortality caused by road traffic injury, male (per 100,000 male population) | -0.096786 |
| Mortality from CVD, cancer, diabetes or CRD between exact ages 30 and 70, female (%) | -0.096766 |
| Mortality from CVD, cancer, diabetes or CRD between exact ages 30 and 70 (%) | -0.096761 |
| Mortality caused by road traffic injury, female (per 100,000 female population) | -0.096759 |
| Mortality from CVD, cancer, diabetes or CRD between exact ages 30 and 70, male (%) | -0.096751 |
| Mortality rate attributed to unintentional poisoning, male (per 100,000 male population) | -0.096739 |
| Mortality rate attributed to unintentional poisoning (per 100,000 population) | -0.096738 |
| Mortality rate attributed to unintentional poisoning, female (per 100,000 female population) | -0.096738 |
| Suicide mortality rate, female (per 100,000 female population) | -0.096728 |
| Suicide mortality rate (per 100,000 population) | -0.096718 |
| Suicide mortality rate, male (per 100,000 male population) | -0.09671 |
| Mortality caused by road traffic injury (per 100,000 people) | -0.096585 |
| Maternal mortality ratio (modeled estimate, per 100,000 live births) | -0.096541 |
| Lifetime risk of maternal death (%) | -0.095432 |
| Prevalence of anemia among children (% of children ages 6-59 months) | -0.09464 |
| Prevalence of anemia among non-pregnant women (% of women ages 15-49) | -0.0946 |
| Prevalence of anemia among pregnant women (%) | -0.0946 |
| Prevalence of anemia among women of reproductive age (% of women ages 15-49) | -0.094597 |
| Prevalence of hypertension, female (% of female adults ages 30-79) | -0.094214 |
| Prevalence of hypertension (% of adults ages 30-79) | -0.0942 |
| Prevalence of hypertension, male (% of male adults ages 30-79) | -0.094186 |
| Treatment for hypertension, male (% of male adults ages 30-79 with hypertension) | -0.094134 |
| Treatment for hypertension (% of adults ages 30-79 with hypertension) | -0.094132 |
| Treatment for hypertension, female (% of female adults ages 30-79 with hypertension) | -0.094125 |
| Domestic general government health expenditure per capita (current US$) | -0.091931 |
| Domestic general government health expenditure per capita, PPP (current international $) | -0.091605 |
| Current health expenditure per capita (current US$) | -0.0869 |
| Current health expenditure per capita, PPP (current international $) | -0.086097 |
| External health expenditure per capita, PPP (current international $) | -0.083694 |
| External health expenditure (% of current health expenditure) | -0.083689 |
| External health expenditure per capita (current US$) | -0.083619 |
| Population ages 50-54, male (% of male population) | 0.079813 |
| Population ages 50-54, female (% of female population) | 0.079615 |
| Population ages 45-49, female (% of female population) | 0.079502 |
| Population ages 55-59, male (% of male population) | 0.078909 |
| Population ages 45-49, male (% of male population) | 0.076332 |
| Population ages 55-59, female (% of female population) | 0.0763 |
| People using at least basic sanitation services, rural (% of rural population) | -0.075698 |
| People using at least basic drinking water services, rural (% of rural population) | -0.075425 |
| People practicing open defecation, rural (% of rural population) | -0.075225 |
| Population ages 60-64, male (% of male population) | 0.075194 |
| Women's share of population ages 15+ living with HIV (%) | -0.074741 |
| Prevalence of stunting, height for age (modeled estimate, % of children under 5) | -0.073957 |
| Immunization, BCG (% of one-year-old children) | -0.073666 |
| Prevalence of overweight (modeled estimate, % of children under 5) | -0.072946 |
| Population ages 40-44, female (% of female population) | 0.072397 |
| Population ages 20-24, female (% of female population) | -0.071719 |
| Population ages 15-19, female (% of female population) | -0.071262 |
| Population ages 20-24, male (% of male population) | -0.071262 |
| Population ages 60-64, female (% of female population) | 0.070395 |
| Population ages 0-14, female (% of female population) | -0.070378 |
| Population ages 65-69, male (% of male population) | 0.070043 |
| Population ages 00-04, female (% of female population) | -0.069843 |
| Population ages 0-14 (% of total population) | -0.069635 |
| Population ages 15-19, male (% of male population) | -0.069311 |
| Population ages 05-09, female (% of female population) | -0.068869 |
| Population ages 10-14, female (% of female population) | -0.068819 |
| Population ages 0-14, male (% of male population) | -0.068541 |
| Population ages 00-04, male (% of male population) | -0.068433 |
| GDP | 0.066993 |
| Age dependency ratio, young | -0.066981 |
| Population ages 05-09, male (% of male population) | -0.066899 |
| Population ages 10-14, male (% of male population) | -0.066274 |
| Population ages 70-74, male (% of male population) | 0.064807 |
| Population ages 65 and above, male (% of male population) | 0.064806 |
| Unemployment, female (% of female labor force) | -0.064589 |
| Unemployment, total (% of total labor force) | -0.064584 |
| Unemployment, male (% of male labor force) | -0.064581 |
| Population ages 40-44, male (% of male population) | 0.064574 |
| Labor force, female (% of total labor force) | -0.064571 |
| Population ages 65-69, female (% of female population) | 0.06345 |
| Prevalence of HIV, total (% of population ages 15-49) | -0.062779 |
| Population ages 65 and above (% of total population) | 0.060731 |
| Low-birthweight babies (% of births) | -0.060573 |
| Survival to age 65, female (% of cohort) | 0.059039 |
| Population ages 75-79, male (% of male population) | 0.05856 |
| Prevalence of undernourishment (% of population) | -0.058287 |
| Age dependency ratio (% of working-age population) | -0.058074 |
| Rural population growth (annual %) | -0.057825 |
| Age dependency ratio, old | 0.057552 |
| Population ages 65 and above, female (% of female population) | 0.057407 |
| Population ages 70-74, female (% of female population) | 0.057212 |
| Adolescent fertility rate (births per 1,000 women ages 15-19) | -0.056652 |
| Population ages 80 and above, male (% of male population) | 0.056578 |
| Lifetime risk of maternal death (1 in: rate varies by country) | -0.05607 |
| Immunization, HepB3 (% of one-year-old children) | -0.056061 |
| Survival to age 65, male (% of cohort) | 0.055737 |
| People practicing open defecation, urban (% of urban population) | -0.05457 |
| People using at least basic sanitation services, urban (% of urban population) | -0.054495 |
| People using at least basic drinking water services, urban (% of urban population) | -0.053048 |
| Tuberculosis case detection rate (%, all forms) | -0.052765 |
| Population ages 80 and older, female (% of female population) | 0.052433 |
| GNI per capita, Atlas method (current US$) | 0.051884 |
| Incidence of tuberculosis (per 100,000 people) | -0.051809 |
| Population ages 35-39, female (% of female population) | 0.051627 |
| Tuberculosis treatment success rate (% of new cases) | -0.051414 |
| Population ages 75-79, female (% of female population) | 0.050918 |
| Tuberculosis death rate (per 100,000 people) | -0.049755 |
| Population ages 25-29, female (% of female population) | -0.044905 |
| Mortality rate, adult, male (per 1,000 male adults) | -0.043837 |
| Mortality rate, adult, female (per 1,000 female adults) | -0.043828 |
| Population ages 35-39, male (% of male population) | 0.04114 |
| People using at least basic sanitation services (% of population) | -0.037281 |
| Population ages 25-29, male (% of male population) | -0.034298 |
| Life expectancy at birth, male (years) | -0.033305 |
| Life expectancy at birth, total (years) | -0.033294 |
| Life expectancy at birth, female (years) | -0.033285 |
| People practicing open defecation (% of population) | -0.032017 |
| Fertility rate, total (births per woman) | -0.030688 |
| Immunization, Hib3 (% of children ages 12-23 months) | -0.027165 |
| People using at least basic drinking water services (% of population) | -0.025982 |
| School enrollment, primary, male (% gross) | -0.025869 |
| School enrollment, primary, female (% gross) | -0.025841 |
| School enrollment, primary (% gross) | -0.025429 |
| Sex ratio at birth (male births per female births) | 0.021915 |
| Rural population (% of total population) | -0.017894 |
| Urban population growth (annual %) | -0.017169 |
| Urban population (% of total population) | -0.015623 |
| Public spending on education, total (% of GDP) | -0.015351 |
| Population, female (% of total population) | 0.014705 |
| Population, male (% of total population) | -0.014705 |
| Population ages 30-34, female (% of female population) | 0.0091963 |
| Rural population | -0.0088484 |
| Birth rate, crude (per 1,000 people) | -0.008806 |
| Death rate, crude (per 1,000 people) | -0.0087604 |
| Population, female | -0.0073613 |
| Population, total | -0.0073236 |
| Population, male | -0.0072865 |
| Net migration | 0.0072046 |
| School enrollment, secondary (% gross) | -0.0068365 |
| Population ages 30-34, male (% of male population) | 0.0062014 |
| Urban population | -0.0040071 |
| Population growth (annual %) | -0.0036466 |
| Labor force, total | 0.0010379 |
