# Supplementary material for: Burden of bacterial antimicrobial resistance associated with modifiable human antibiotic consumption: An observational study
Source: PLoS Med. 2026 Oct 26;23(10):e1005140. doi: 10.1371/journal.pmed.1005140 (PMC13719055; doi:10.1371/journal.pmed.1005140)
Supplement: Supplementary file 31 [file pmed.1005140.s008.docx]

**S6 Table: Loadings of World Bank Health, Nutrition, and Population Statistics for the second principal component.**

| **Variable** | **Loading** |
| --- | --- |
| Population ages 75-79, female (% of female population) | -0.14655 |
| Population ages 10-14, female (% of female population) | 0.14652 |
| Population ages 0-14, female (% of female population) | 0.1457 |
| Population ages 65 and above, female (% of female population) | -0.14557 |
| Population ages 05-09, female (% of female population) | 0.14551 |
| Population ages 0-14 (% of total population) | 0.14518 |
| Population ages 65 and above (% of total population) | -0.14349 |
| Population ages 80 and older, female (% of female population) | -0.14345 |
| Population ages 05-09, male (% of male population) | 0.14344 |
| Population ages 0-14, male (% of male population) | 0.14341 |
| Population ages 10-14, male (% of male population) | 0.14196 |
| Population ages 70-74, female (% of female population) | -0.14131 |
| Population ages 75-79, male (% of male population) | -0.14025 |
| Population ages 00-04, female (% of female population) | 0.13966 |
| Age dependency ratio, young | 0.13906 |
| Age dependency ratio, old | -0.13905 |
| Population ages 65 and above, male (% of male population) | -0.13878 |
| Population ages 00-04, male (% of male population) | 0.13836 |
| Population ages 65-69, female (% of female population) | -0.13761 |
| Population ages 15-19, female (% of female population) | 0.13638 |
| Population ages 70-74, male (% of male population) | -0.13596 |
| Population ages 60-64, female (% of female population) | -0.13382 |
| Population ages 65-69, male (% of male population) | -0.13376 |
| Population ages 80 and above, male (% of male population) | -0.13345 |
| Population ages 55-59, male (% of male population) | -0.13276 |
| Population ages 50-54, male (% of male population) | -0.13261 |
| Population ages 60-64, male (% of male population) | -0.13243 |
| Population ages 55-59, female (% of female population) | -0.13098 |
| Population ages 15-19, male (% of male population) | 0.12811 |
| Population ages 50-54, female (% of female population) | -0.12799 |
| Population ages 45-49, male (% of male population) | -0.12646 |
| Survival to age 65, female (% of cohort) | -0.12081 |
| Population ages 45-49, female (% of female population) | -0.12028 |
| Adolescent fertility rate (births per 1,000 women ages 15-19) | 0.11729 |
| Age dependency ratio (% of working-age population) | 0.11153 |
| Population ages 40-44, male (% of male population) | -0.11113 |
| Survival to age 65, male (% of cohort) | -0.11103 |
| Population ages 20-24, female (% of female population) | 0.11055 |
| Lifetime risk of maternal death (1 in: rate varies by country) | -0.10647 |
| Population ages 40-44, female (% of female population) | -0.10513 |
| Current health expenditure per capita, PPP (current international $) | -0.094883 |
| Current health expenditure per capita (current US$) | -0.094114 |
| Population ages 20-24, male (% of male population) | 0.094036 |
| GNI per capita, Atlas method (current US$) | -0.091861 |
| GDP | -0.089643 |
| Domestic general government health expenditure per capita, PPP (current international $) | -0.086699 |
| Domestic general government health expenditure per capita (current US$) | -0.086106 |
| Population ages 35-39, male (% of male population) | -0.081874 |
| Domestic private health expenditure per capita, PPP (current international $) | -0.076503 |
| Domestic private health expenditure per capita (current US$) | -0.076325 |
| Out-of-pocket expenditure per capita, PPP (current international $) | -0.075812 |
| Low-birthweight babies (% of births) | -0.075331 |
| Out-of-pocket expenditure per capita (current US$) | -0.073595 |
| Population ages 35-39, female (% of female population) | -0.073333 |
| Current health expenditure (% of GDP) | -0.071223 |
| Domestic general government health expenditure (% of current health expenditure) | -0.069684 |
| Domestic general government health expenditure (% of general government expenditure) | -0.069429 |
| Domestic general government health expenditure (% of GDP) | -0.069401 |
| Domestic private health expenditure (% of current health expenditure) | -0.06925 |
| Out-of-pocket expenditure (% of current health expenditure) | -0.069031 |
| Immunization, BCG (% of one-year-old children) | 0.061601 |
| School enrollment, secondary (% gross) | -0.059611 |
| Stillbirth rate (per 1,000 total births) | -0.056822 |
| Prevalence of stunting, height for age (modeled estimate, % of children under 5) | 0.055084 |
| Sex ratio at birth (male births per female births) | -0.054579 |
| Public spending on education, total (% of GDP) | -0.054147 |
| Population ages 25-29, female (% of female population) | 0.05284 |
| Mortality caused by road traffic injury (per 100,000 people) | -0.050672 |
| Suicide mortality rate, male (per 100,000 male population) | -0.050459 |
| Suicide mortality rate (per 100,000 population) | -0.050432 |
| Suicide mortality rate, female (per 100,000 female population) | -0.050405 |
| Mortality rate attributed to unintentional poisoning, female (per 100,000 female population) | -0.05038 |
| Mortality rate attributed to unintentional poisoning (per 100,000 population) | -0.050379 |
| Mortality rate attributed to unintentional poisoning, male (per 100,000 male population) | -0.050378 |
| Mortality from CVD, cancer, diabetes or CRD between exact ages 30 and 70, male (%) | -0.050331 |
| Mortality caused by road traffic injury, female (per 100,000 female population) | -0.05032 |
| Mortality from CVD, cancer, diabetes or CRD between exact ages 30 and 70 (%) | -0.050312 |
| Mortality from CVD, cancer, diabetes or CRD between exact ages 30 and 70, female (%) | -0.050295 |
| Mortality caused by road traffic injury, male (per 100,000 male population) | -0.050246 |
| Total alcohol consumption per capita, male (liters of pure alcohol, projected estimates, male 15+ years of age) | -0.050008 |
| Total alcohol consumption per capita (liters of pure alcohol, projected estimates, 15+ years of age) | -0.049977 |
| Total alcohol consumption per capita, female (liters of pure alcohol, projected estimates, female 15+ years of age) | -0.049948 |
| Prevalence of anemia among non-pregnant women (% of women ages 15-49) | -0.049866 |
| Prevalence of anemia among pregnant women (%) | -0.049862 |
| Prevalence of anemia among women of reproductive age (% of women ages 15-49) | -0.049861 |
| Prevalence of anemia among children (% of children ages 6-59 months) | -0.049748 |
| Immunization, Hib3 (% of children ages 12-23 months) | -0.049249 |
| Lifetime risk of maternal death (%) | -0.047763 |
| School enrollment, primary, female (% gross) | -0.047354 |
| School enrollment, primary, male (% gross) | -0.047308 |
| Immunization, measles (% of children ages 12-23 months) | -0.046669 |
| Immunization, Pol3 (% of one-year-old children) | -0.046667 |
| Immunization, DPT (% of children ages 12-23 months) | -0.046664 |
| Treatment for hypertension, female (% of female adults ages 30-79 with hypertension) | -0.046589 |
| Treatment for hypertension (% of adults ages 30-79 with hypertension) | -0.046569 |
| School enrollment, primary (% gross) | -0.046564 |
| Treatment for hypertension, male (% of male adults ages 30-79 with hypertension) | -0.046558 |
| Prevalence of hypertension, male (% of male adults ages 30-79) | -0.04641 |
| Prevalence of hypertension (% of adults ages 30-79) | -0.046368 |
| Prevalence of hypertension, female (% of female adults ages 30-79) | -0.046326 |
| Prevalence of overweight (modeled estimate, % of children under 5) | 0.046141 |
| Maternal mortality ratio (modeled estimate, per 100,000 live births) | -0.044521 |
| Probability of dying among adolescents ages 10-14 years, female (per 1,000) | -0.043848 |
| Probability of dying among adolescents ages 10-14 years (per 1,000) | -0.043846 |
| Probability of dying among adolescents ages 10-14 years, male (per 1,000) | -0.043844 |
| Probability of dying among adolescents ages 15-19 years, female (per 1,000) | -0.043812 |
| Probability of dying among adolescents ages 15-19 years (per 1,000) | -0.04381 |
| Probability of dying among adolescents ages 15-19 years, male (per 1,000) | -0.043808 |
| Probability of dying among children ages 5-9 years, female (per 1,000) | -0.0438 |
| Probability of dying among children ages 5-9 years (per 1,000) | -0.043795 |
| Probability of dying among children ages 5-9 years, male (per 1,000) | -0.043792 |
| Probability of dying among youth ages 20-24 years, male (per 1,000) | -0.043778 |
| Probability of dying among youth ages 20-24 years (per 1,000) | -0.043774 |
| Probability of dying among youth ages 20-24 years, female (per 1,000) | -0.043768 |
| Probability of dying among adolescents ages 10-19 years, female (per 1,000) | -0.043743 |
| Probability of dying among adolescents ages 10-19 years (per 1,000) | -0.04374 |
| Probability of dying among adolescents ages 10-19 years, male (per 1,000) | -0.043738 |
| Mortality rate, neonatal (per 1,000 live births) | -0.043585 |
| Mortality rate, infant, female (per 1,000 live births) | -0.043337 |
| Mortality rate, infant (per 1,000 live births) | -0.043278 |
| Mortality rate, infant, male (per 1,000 live births) | -0.043218 |
| Mortality rate, under-5, female (per 1,000) | -0.043018 |
| Mortality rate, under-5 (per 1,000) | -0.042949 |
| Mortality rate, under-5, male (per 1,000) | -0.042887 |
| Women's share of population ages 15+ living with HIV (%) | -0.039383 |
| Population ages 30-34, male (% of male population) | -0.039139 |
| Prevalence of undernourishment (% of population) | -0.034826 |
| Net migration | -0.031015 |
| Labor force, female (% of total labor force) | -0.0265 |
| Unemployment, male (% of male labor force) | -0.026466 |
| Unemployment, total (% of total labor force) | -0.026461 |
| Unemployment, female (% of female labor force) | -0.026448 |
| Urban population | -0.024196 |
| Tuberculosis death rate (per 100,000 people) | -0.023826 |
| Rural population growth (annual %) | -0.02302 |
| Tuberculosis case detection rate (%, all forms) | -0.020815 |
| People using at least basic sanitation services (% of population) | -0.020725 |
| Mortality rate, adult, female (per 1,000 female adults) | 0.020504 |
| Mortality rate, adult, male (per 1,000 male adults) | 0.020459 |
| Incidence of tuberculosis (per 100,000 people) | -0.019332 |
| Population ages 30-34, female (% of female population) | -0.01895 |
| People practicing open defecation (% of population) | -0.018907 |
| Urban population (% of total population) | -0.01729 |
| Population, female (% of total population) | -0.01677 |
| Population, male (% of total population) | 0.01677 |
| Urban population growth (annual %) | -0.015679 |
| Population ages 25-29, male (% of male population) | 0.014654 |
| Immunization, HepB3 (% of one-year-old children) | -0.014522 |
| Rural population (% of total population) | -0.014465 |
| Labor force, total | -0.014437 |
| Population, female | -0.012783 |
| Birth rate, crude (per 1,000 people) | 0.012771 |
| Death rate, crude (per 1,000 people) | 0.012572 |
| Population, total | -0.012337 |
| Population, male | -0.01191 |
| Fertility rate, total (births per woman) | 0.011195 |
| Life expectancy at birth, male (years) | 0.011169 |
| Life expectancy at birth, total (years) | 0.011147 |
| Life expectancy at birth, female (years) | 0.011123 |
| People using at least basic drinking water services (% of population) | -0.0089238 |
| Tuberculosis treatment success rate (% of new cases) | 0.008122 |
| People using at least basic sanitation services, rural (% of rural population) | -0.006572 |
| People practicing open defecation, rural (% of rural population) | -0.0065335 |
| Prevalence of HIV, total (% of population ages 15-49) | 0.0034692 |
| People using at least basic drinking water services, urban (% of urban population) | 0.0032645 |
| People using at least basic drinking water services, rural (% of rural population) | -0.0016832 |
| People practicing open defecation, urban (% of urban population) | 0.001675 |
| Population growth (annual %) | -0.0013629 |
| Rural population | -0.0013384 |
| People using at least basic sanitation services, urban (% of urban population) | 0.0013176 |
| External health expenditure per capita (current US$) | -0.00010778 |
| External health expenditure (% of current health expenditure) | -7.7899e-05 |
| External health expenditure per capita, PPP (current international $) | 7.4302e-05 |
