# Supplementary material for: Burden of bacterial antimicrobial resistance associated with modifiable human antibiotic consumption: An observational study
Source: PLoS Med. 2026 Oct 26;23(10):e1005140. doi: 10.1371/journal.pmed.1005140 (PMC13719055; doi:10.1371/journal.pmed.1005140)
Supplement: Supplementary file 32 [file pmed.1005140.s009.docx]

**S7 Table: Loadings of World Bank Health, Nutrition, and Population Statistics for the third principal component.**

| **Variable** | **Loading** |
| --- | --- |
| Suicide mortality rate (per 100,000 population) | 0.14889 |
| Suicide mortality rate, female (per 100,000 female population) | 0.14889 |
| Suicide mortality rate, male (per 100,000 male population) | 0.14889 |
| Mortality from CVD, cancer, diabetes or CRD between exact ages 30 and 70, male (%) | 0.14888 |
| Mortality rate attributed to unintentional poisoning (per 100,000 population) | 0.14888 |
| Mortality rate attributed to unintentional poisoning, female (per 100,000 female population) | 0.14888 |
| Mortality rate attributed to unintentional poisoning, male (per 100,000 male population) | 0.14888 |
| Mortality caused by road traffic injury, female (per 100,000 female population) | 0.14887 |
| Mortality from CVD, cancer, diabetes or CRD between exact ages 30 and 70 (%) | 0.14887 |
| Mortality from CVD, cancer, diabetes or CRD between exact ages 30 and 70, female (%) | 0.14887 |
| Mortality caused by road traffic injury, male (per 100,000 male population) | 0.14885 |
| Mortality caused by road traffic injury (per 100,000 people) | 0.14752 |
| Treatment for hypertension (% of adults ages 30-79 with hypertension) | 0.13855 |
| Treatment for hypertension, female (% of female adults ages 30-79 with hypertension) | 0.13855 |
| Treatment for hypertension, male (% of male adults ages 30-79 with hypertension) | 0.13855 |
| Prevalence of hypertension, male (% of male adults ages 30-79) | 0.13853 |
| Prevalence of hypertension (% of adults ages 30-79) | 0.13852 |
| Prevalence of hypertension, female (% of female adults ages 30-79) | 0.13852 |
| Probability of dying among adolescents ages 10-14 years (per 1,000) | -0.13141 |
| Probability of dying among adolescents ages 10-14 years, female (per 1,000) | -0.13141 |
| Probability of dying among adolescents ages 10-14 years, male (per 1,000) | -0.13141 |
| Probability of dying among adolescents ages 15-19 years (per 1,000) | -0.1314 |
| Probability of dying among adolescents ages 15-19 years, female (per 1,000) | -0.1314 |
| Probability of dying among adolescents ages 15-19 years, male (per 1,000) | -0.1314 |
| Probability of dying among youth ages 20-24 years, male (per 1,000) | -0.1314 |
| Probability of dying among children ages 5-9 years (per 1,000) | -0.13139 |
| Probability of dying among children ages 5-9 years, female (per 1,000) | -0.13139 |
| Probability of dying among children ages 5-9 years, male (per 1,000) | -0.13139 |
| Probability of dying among youth ages 20-24 years (per 1,000) | -0.13139 |
| Probability of dying among adolescents ages 10-19 years (per 1,000) | -0.13138 |
| Probability of dying among adolescents ages 10-19 years, male (per 1,000) | -0.13138 |
| Probability of dying among youth ages 20-24 years, female (per 1,000) | -0.13138 |
| Probability of dying among adolescents ages 10-19 years, female (per 1,000) | -0.13137 |
| Mortality rate, neonatal (per 1,000 live births) | -0.13133 |
| Mortality rate, infant, female (per 1,000 live births) | -0.13122 |
| Mortality rate, infant (per 1,000 live births) | -0.1312 |
| Mortality rate, infant, male (per 1,000 live births) | -0.13119 |
| Mortality rate, under-5, female (per 1,000) | -0.13108 |
| Mortality rate, under-5 (per 1,000) | -0.13106 |
| Mortality rate, under-5, male (per 1,000) | -0.13104 |
| Prevalence of anemia among women of reproductive age (% of women ages 15-49) | 0.12436 |
| Prevalence of anemia among non-pregnant women (% of women ages 15-49) | 0.12435 |
| Prevalence of anemia among pregnant women (%) | 0.12435 |
| Prevalence of anemia among children (% of children ages 6-59 months) | 0.12434 |
| Lifetime risk of maternal death (%) | 0.11569 |
| Maternal mortality ratio (modeled estimate, per 100,000 live births) | 0.11543 |
| Immunization, DPT (% of children ages 12-23 months) | -0.11261 |
| Immunization, measles (% of children ages 12-23 months) | -0.11259 |
| Immunization, Pol3 (% of one-year-old children) | -0.11259 |
| Immunization, Hib3 (% of children ages 12-23 months) | -0.098153 |
| Immunization, HepB3 (% of one-year-old children) | -0.096609 |
| Lifetime risk of maternal death (1 in: rate varies by country) | 0.094453 |
| Total alcohol consumption per capita, male (liters of pure alcohol, projected estimates, male 15+ years of age) | 0.092765 |
| Total alcohol consumption per capita (liters of pure alcohol, projected estimates, 15+ years of age) | 0.092761 |
| Total alcohol consumption per capita, female (liters of pure alcohol, projected estimates, female 15+ years of age) | 0.092757 |
| Mortality rate, adult, female (per 1,000 female adults) | 0.065142 |
| Mortality rate, adult, male (per 1,000 male adults) | 0.065025 |
| Low-birthweight babies (% of births) | 0.055757 |
| People using at least basic drinking water services, rural (% of rural population) | -0.051317 |
| People using at least basic sanitation services, rural (% of rural population) | -0.047985 |
| Life expectancy at birth, total (years) | 0.045073 |
| Life expectancy at birth, male (years) | 0.045072 |
| Life expectancy at birth, female (years) | 0.045065 |
| Fertility rate, total (births per woman) | 0.044443 |
| People practicing open defecation, rural (% of rural population) | -0.044319 |
| Rural population growth (annual %) | -0.043534 |
| Tuberculosis death rate (per 100,000 people) | -0.042555 |
| Incidence of tuberculosis (per 100,000 people) | -0.041574 |
| Immunization, BCG (% of one-year-old children) | -0.03854 |
| Prevalence of overweight (modeled estimate, % of children under 5) | -0.037064 |
| Prevalence of stunting, height for age (modeled estimate, % of children under 5) | -0.035864 |
| Population ages 30-34, male (% of male population) | -0.035363 |
| Death rate, crude (per 1,000 people) | 0.033348 |
| Tuberculosis case detection rate (%, all forms) | -0.03135 |
| People using at least basic drinking water services, urban (% of urban population) | -0.031298 |
| Birth rate, crude (per 1,000 people) | 0.030865 |
| Population ages 35-39, male (% of male population) | -0.030518 |
| People using at least basic sanitation services, urban (% of urban population) | -0.028722 |
| People practicing open defecation, urban (% of urban population) | -0.028687 |
| GDP | 0.027502 |
| Urban population growth (annual %) | 0.027167 |
| Public spending on education, total (% of GDP) | -0.026174 |
| Urban population (% of total population) | 0.025509 |
| Rural population (% of total population) | 0.025438 |
| Population ages 30-34, female (% of female population) | -0.025269 |
| Tuberculosis treatment success rate (% of new cases) | -0.023955 |
| Women's share of population ages 15+ living with HIV (%) | -0.023271 |
| Population ages 15-19, male (% of male population) | 0.022602 |
| Population, female (% of total population) | 0.022256 |
| Population, male (% of total population) | -0.022256 |
| People practicing open defecation (% of population) | 0.022137 |
| Adolescent fertility rate (births per 1,000 women ages 15-19) | 0.021638 |
| People using at least basic drinking water services (% of population) | 0.02118 |
| People using at least basic sanitation services (% of population) | 0.021165 |
| Population ages 20-24, male (% of male population) | 0.01971 |
| School enrollment, primary (% gross) | -0.018897 |
| Population ages 20-24, female (% of female population) | 0.018672 |
| School enrollment, primary, female (% gross) | -0.018014 |
| School enrollment, primary, male (% gross) | -0.017995 |
| Population ages 35-39, female (% of female population) | -0.017253 |
| Population ages 15-19, female (% of female population) | 0.017005 |
| School enrollment, secondary (% gross) | -0.01612 |
| Population ages 25-29, male (% of male population) | -0.015529 |
| Urban population | -0.015155 |
| Population ages 80 and older, female (% of female population) | -0.013899 |
| Population ages 80 and above, male (% of male population) | -0.013879 |
| Population ages 45-49, female (% of female population) | 0.013205 |
| Prevalence of undernourishment (% of population) | -0.013085 |
| Prevalence of HIV, total (% of population ages 15-49) | -0.01248 |
| Population ages 60-64, female (% of female population) | -0.012457 |
| GNI per capita, Atlas method (current US$) | 0.012249 |
| Labor force, total | -0.011717 |
| Population ages 65-69, female (% of female population) | -0.011603 |
| Population, female | -0.011503 |
| Population, total | -0.0114 |
| Population, male | -0.011301 |
| Population ages 00-04, male (% of male population) | 0.010637 |
| Population growth (annual %) | 0.010001 |
| Population ages 65 and above, female (% of female population) | -0.0097965 |
| Sex ratio at birth (male births per female births) | -0.009257 |
| External health expenditure per capita, PPP (current international $) | 0.009236 |
| External health expenditure (% of current health expenditure) | 0.0091338 |
| Population ages 70-74, female (% of female population) | -0.0090812 |
| External health expenditure per capita (current US$) | 0.0089615 |
| Survival to age 65, female (% of cohort) | -0.0088687 |
| Population ages 50-54, female (% of female population) | 0.0088056 |
| Age dependency ratio, old | -0.008803 |
| Current health expenditure per capita, PPP (current international $) | -0.0084388 |
| Rural population | -0.0084294 |
| Population ages 40-44, male (% of male population) | -0.0084266 |
| Population ages 65 and above (% of total population) | -0.0077831 |
| Population ages 00-04, female (% of female population) | 0.0075502 |
| Population ages 10-14, male (% of male population) | 0.0075319 |
| Population ages 0-14, male (% of male population) | 0.0074857 |
| Age dependency ratio, young | 0.007438 |
| Current health expenditure per capita (current US$) | -0.007131 |
| Labor force, female (% of total labor force) | 0.0070479 |
| Unemployment, male (% of male labor force) | 0.0070329 |
| Unemployment, total (% of total labor force) | 0.0070306 |
| Unemployment, female (% of female labor force) | 0.0070259 |
| Population ages 60-64, male (% of male population) | -0.0058869 |
| Survival to age 65, male (% of cohort) | -0.0058616 |
| Population ages 50-54, male (% of male population) | 0.0056375 |
| Population ages 0-14 (% of total population) | 0.0055636 |
| Population ages 45-49, male (% of male population) | 0.0055153 |
| Current health expenditure (% of GDP) | -0.0053533 |
| Age dependency ratio (% of working-age population) | 0.0053345 |
| Out-of-pocket expenditure per capita, PPP (current international $) | -0.0052802 |
| Population ages 65 and above, male (% of male population) | -0.0052773 |
| Population ages 40-44, female (% of female population) | 0.0047438 |
| Population ages 65-69, male (% of male population) | -0.004311 |
| Population ages 55-59, female (% of female population) | -0.0042282 |
| Population ages 25-29, female (% of female population) | -0.0038037 |
| Population ages 70-74, male (% of male population) | -0.0035061 |
| Population ages 0-14, female (% of female population) | 0.0034611 |
| Population ages 05-09, male (% of male population) | 0.0030625 |
| Population ages 10-14, female (% of female population) | 0.0024319 |
| Domestic general government health expenditure per capita, PPP (current international $) | -0.0017596 |
| Population ages 75-79, male (% of male population) | 0.0016163 |
| Population ages 55-59, male (% of male population) | -0.0013832 |
| Net migration | -0.0012978 |
| Population ages 05-09, female (% of female population) | -0.0010245 |
| Out-of-pocket expenditure per capita (current US$) | 0.00099052 |
| Stillbirth rate (per 1,000 total births) | 0.00084757 |
| Out-of-pocket expenditure (% of current health expenditure) | 0.00084215 |
| Population ages 75-79, female (% of female population) | -0.000788 |
| Domestic general government health expenditure per capita (current US$) | -0.00053774 |
| Domestic private health expenditure per capita (current US$) | 0.00046192 |
| Domestic private health expenditure per capita, PPP (current international $) | 0.00038586 |
| Domestic private health expenditure (% of current health expenditure) | 0.00037718 |
| Domestic general government health expenditure (% of GDP) | 0.00031665 |
| Domestic general government health expenditure (% of general government expenditure) | 0.0003121 |
| Domestic general government health expenditure (% of current health expenditure) | 0.00028831 |
