# Supplementary material for: Burden of bacterial antimicrobial resistance associated with modifiable human antibiotic consumption: An observational study
Source: PLoS Med. 2026 Oct 26;23(10):e1005140. doi: 10.1371/journal.pmed.1005140 (PMC13719055; doi:10.1371/journal.pmed.1005140)
Supplement: Supplementary file 33 [file pmed.1005140.s010.docx]

**S8 Table: Correlation between consumption and other model variables for each antibiotic class.**

| **Variable** | **Tetracyclines** | **Penicillins** | **Non-Penicillin Beta-Lactams** | **Sulfonamides and Trimethoprim** | **Macrolides** | **Aminoglycosides** | **Quinolones** |
| --- | --- | --- | --- | --- | --- | --- | --- |
| Year | -0.068 | 0.149 | 0.055 | -0.22 | -0.113 | 0.019 | 0.071 |
| GDP | 0.420 | 0.298 | 0.009 | 0.019 | 0.201 | -0.140 | -0.050 |
| PC1 | 0.202 | 0.417 | 0.254 | -0.108 | 0.410 | 0.089 | 0.252 |
| PC2 | -0.258 | -0.414 | -0.222 | 0.084 | -0.416 | -0.05 | -0.228 |
| PC3 | 0.077 | -0.096 | -0.228 | 0.103 | -0.067 | -0.011 | -0.195 |
