# Supplementary material for: Burden of bacterial antimicrobial resistance associated with modifiable human antibiotic consumption: An observational study
Source: PLoS Med. 2026 Oct 26;23(10):e1005140. doi: 10.1371/journal.pmed.1005140 (PMC13719055; doi:10.1371/journal.pmed.1005140)
Supplement: Supplementary file 34 [file pmed.1005140.s011.docx]

**Table S9: Measures of model fit for each regression at the antibiotic class-pathogen level, along with *p*-values of the White test for homoskedasticity.**

| **Antibiotic class** | **Pathogen** | **Elasticity** | ***R2*** | **RMSE** | **AIC** | **BIC** | **White *p*-value** |
| --- | --- | --- | --- | --- | --- | --- | --- |
| Tetracyclines | *Acinetobacter* spp. | 0.10 (-0.24, 0.64) | 0.2783 | 1.546 | 2017 | 2051 | 4.123e-08 |
|  | *E. coli* | -0.11 (-0.23, 0.09) | 0.4202 | 1.154 | 2479 | 2516 | 2.107e-05 |
|  | *E. faecalis* | -0.05 (-0.22, 0.14) | 0.2492 | 1.489 | 2147 | 2182 | 3.13e-08 |
|  | *E. faecium* | 0.16 (-0.08, 0.38) | 0.1337 | 1.65 | 2125 | 2159 | 7.852e-18 |
|  | *Enterococcus* spp. | 0.06 (-0.33, 0.36) | 0.1317 | 1.662 | 1158 | 1187 | 2.923e-11 |
|  | *H. influenzae* | 0.02 (-0.05, 0.07) | 0.03953 | 0.5293 | 994.8 | 1029 | 0.8864 |
|  | *K. pneumoniae* | -0.09 (-0.22, 0.09) | 0.3745 | 1.193 | 2574 | 2611 | 0.02755 |
|  | *Morganella* spp. | 0.06 (-0.23, 0.41) | 0.04858 | 1.11 | 804.5 | 832.7 | 0.0003412 |
|  | *S. agalactiae* | 0.05 (-0.10, 0.25) | 0.2515 | 1.609 | 2059 | 2094 | 3.073e-17 |
|  | *S. aureus* | 0.09 (-0.21, 0.35) | 0.06657 | 0.8152 | 1890 | 1925 | 4.83e-21 |
|  | *S. pyogenes* | -0.02 (-0.14, 0.15) | 0.2016 | 0.4767 | 377.8 | 406.3 | 0.005401 |
| Penicillins | *Acinetobacter* spp. | 0.32 (0.02, 0.61) | 0.3085 | 1.234 | 1715 | 1750 | 2.186e-07 |
|  | *E. coli* | 0.02 (-0.04, 0.09) | 0.4072 | 0.2484 | -360.5 | -325.2 | 8.376e-06 |
|  | *E. faecalis* | -0.03 (-0.28, 0.22) | 0.05111 | 0.8771 | 1843 | 1879 | 0.001491 |
|  | *E. faecium* | 0.03 (-0.02, 0.11) | 0.02932 | 0.6763 | 648.1 | 683.3 | 0.5191 |
|  | *Enterococcus* spp. | -0.19 (-0.80, 0.65) | 0.107 | 2.19 | 1263 | 1292 | 0.0129 |
|  | *H. influenzae* | 0.21 (-0.14, 0.48) | 0.09777 | 1.425 | 2201 | 2237 | 0.002555 |
|  | *K. pneumoniae* | 0.01 (-0.15, 0.28) | 0.3102 | 0.7178 | 2185 | 2221 | 1.897e-41 |
|  | *Morganella* spp. | 0.00 (-0.00, 0.00) | 0.01209 | 0.007754 | -1924 | -1896 | 0.8013 |
|  | *P. aeruginosa* | 0.04 (-0.23, 0.30) | 0.3041 | 0.8116 | 2090 | 2129 | 3.268e-14 |
|  | *S. aureus* | 0.75 (0.16, 1.58) | 0.4112 | 1.083 | 2382 | 2419 | 6.697e-61 |
|  | *S. pneumoniae* | 0.32 (-0.31, 0.93) | 0.2987 | 1.252 | 3404 | 3442 | 3.474e-14 |
| Non-penicillin beta-lactams | *Acinetobacter* spp. | 0.30 (-0.31, 0.73) | 0.3263 | 1.272 | 4137 | 4176 | 2.223e-18 |
|  | *E. coli* | 0.28 (0.08, 0.38) | 0.07678 | 1.597 | 11930 | 11970 | 7.49e-81 |
|  | *K. pneumoniae* | 0.10 (-0.18, 0.55) | 0.3084 | 1.618 | 9194 | 9238 | 1.178e-246 |
|  | *Morganella* spp. | 0.02 (-0.05, 0.07) | 0.01127 | 0.5944 | 195.4 | 223.5 | 0.1284 |
|  | *P. aeruginosa* | 0.35 (0.13, 0.55) | 0.4965 | 0.9012 | 3438 | 3480 | 1.534e-14 |
|  | *S. aureus* | -0.01 (-0.07, 0.02) | 0.06964 | 0.1454 | -151.9 | -123.7 | 0.1095 |
|  | *S. pneumoniae* | 0.19 (-0.13, 0.61) | 0.1757 | 1.361 | 3387 | 3425 | 4.851e-30 |
|  | *Salmonella* spp. | -0.21 (-0.67, 0.42) | 0.1368 | 0.8373 | 197.9 | 215.6 | 0.06804 |
| Sulfonamides and trimethoprim | *Acinetobacter* spp. | 0.11 (-0.08, 0.31) | 0.4091 | 0.904 | 109.3 | 122.6 | 0.07222 |
|  | *E. coli* | 0.00 (-0.36, 0.24) | 0.6039 | 0.4223 | 242.6 | 261.7 | 0.06481 |
|  | *K. pneumoniae* | 0.09 (-0.30, 0.38) | 0.6818 | 0.5335 | 230.2 | 249.5 | 3.246e-07 |
|  | *Morganella* spp. | -0.13 (-0.43, 0.35) | 0.2381 | 1.281 | 167.9 | 181.4 | 0.6795 |
|  | *S. aureus* | -0.09 (-0.32, 0.48) | 0.1872 | 0.7227 | 414.1 | 439.8 | 7.337e-05 |
| Macrolides | *E. faecalis* | 0.09 (0.01, 0.26) | 0.5578 | 0.306 | 47.79 | 72.19 | 0.001108 |
|  | *E. faecium* | 0.04 (-0.06, 0.11) | 0.0933 | 0.517 | -21.06 | 3.126 | 0.2853 |
|  | *S. agalactiae* | -0.20 (-0.82, 0.61) | 0.1101 | 1.501 | 918.8 | 947.5 | 0.2825 |
|  | *S. aureus* | 0.20 (-0.17, 0.57) | 0.2504 | 0.5921 | 367.1 | 396.1 | 0.03278 |
|  | *S. pneumoniae* | 0.68 (-0.03, 1.27) | 0.3875 | 1.205 | 3311 | 3350 | 5.591e-14 |
|  | *S. pyogenes* | -0.26 (-1.03, 0.64) | 0.06745 | 1.374 | 885.2 | 913.2 | 0.01903 |
| Aminoglycosides | *Acinetobacter* spp. | 0.15 (-0.26, 0.52) | 0.3885 | 1.319 | 2936 | 2973 | 1.152e-09 |
|  | *E. coli* | 0.21 (-0.01, 0.32) | 0.4515 | 0.9077 | 2754 | 2794 | 8.235e-15 |
|  | *E. faecalis* | 0.08 (-0.33, 0.44) | 0.2384 | 0.371 | 411.8 | 441.4 | 6.079e-06 |
|  | *E. faecium* | 0.31 (-0.17, 0.69) | 0.2239 | 0.4489 | 284 | 310.4 | 0.000393 |
|  | *K. pneumoniae* | 0.43 (0.12, 0.72) | 0.5195 | 1.204 | 3044 | 3083 | 1.95e-09 |
|  | *Morganella* spp. | 0.28 (-0.04, 0.55) | 0.2086 | 1.273 | 868.1 | 896.3 | 0.000191 |
|  | *P. aeruginosa* | 0.22 (-0.09, 0.56) | 0.4356 | 1.152 | 2837 | 2876 | 1.836e-13 |
|  | *S. aureus* | 0.09 (-0.34, 0.46) | 0.2912 | 0.8596 | 490.5 | 516.2 | 0.0002177 |
| Quinolones | *Acinetobacter* spp. | 1.16 (0.58, 1.46) | 0.6891 | 1.202 | 2096 | 2133 | 9.178e-13 |
|  | *E. coli* | 0.70 (0.40, 0.83) | 0.8249 | 0.6164 | 2969 | 3009 | 2.956e-28 |
|  | *E. faecalis* | 0.45 (0.19, 0.66) | 0.1587 | 0.9046 | 1342 | 1377 | 0.2115 |
|  | *E. faecium* | 0.01 (-0.08, 0.08) | 0.0635 | 0.6776 | 382.1 | 416.6 | 0.6403 |
|  | *Enterococcus* spp. | 0.83 (-0.09, 1.33) | 0.1869 | 2.008 | 1174 | 1203 | 0.000866 |
|  | *K. pneumoniae* | 0.12 (-0.20, 0.70) | 0.6027 | 1.079 | 3591 | 3630 | 1.687e-192 |
|  | *Morganella* spp. | 0.56 (0.17, 1.00) | 0.149 | 1.305 | 815.9 | 844 | 0.2013 |
|  | *P. aeruginosa* | 0.63 (0.31, 0.81) | 0.6453 | 0.679 | 1610 | 1649 | 1.609e-06 |
|  | *S. agalactiae* | 0.32 (-0.10, 0.63) | 0.1479 | 1.035 | 1736 | 1771 | 2.395e-14 |
|  | *S. aureus* | 0.36 (-0.01, 0.71) | 0.1425 | 1.044 | 1911 | 1948 | 6.901e-09 |
|  | *S. pneumoniae* | 0.28 (-0.06, 0.50) | 0.07464 | 0.5727 | 1635 | 1671 | 7.419e-11 |
|  | *S. pyogenes* | 0.05 (-0.05, 0.15) | 0.07142 | 0.2991 | 249.5 | 278 | 0.04087 |
|  | *Salmonella* spp. | 0.79 (-0.53, 1.42) | 0.8004 | 0.849 | 81.8 | 93.01 | 0.738 |
