# Supplementary material for: Burden of bacterial antimicrobial resistance associated with modifiable human antibiotic consumption: An observational study
Source: PLoS Med. 2026 Oct 26;23(10):e1005140. doi: 10.1371/journal.pmed.1005140 (PMC13719055; doi:10.1371/journal.pmed.1005140)
Supplement: Supplementary file 35 [file pmed.1005140.s012.docx]

**Table S10: Measures of model fit for each regression at the pathogen level, along with *p*-values of the White test for homoskedasticity.**

| **Antibiotic class** | **Elasticity** | ***R*^2^** | **RMSE** | **AIC** | **BIC** | **White *p*-value** |
| --- | --- | --- | --- | --- | --- | --- |
| *Acinetobacter* spp. | 0.43 (0.19, 0.56) | 1.000 | 1.305 | 14034 | 14096 | 1.411e-37 |
| *E. coli* | 0.27 (0.17, 0.33) | 0.9976 | 1.211 | 31530 | 31596 | 8.677e-192 |
| *E. faecalis* | 0.11 (-0.36, 0.24) | 1.000 | 1.064 | 6845 | 6902 | 3.663e-44 |
| *E. faecium* | 0.17 (-0.03, 0.25) | 1.000 | 1.032 | 6016 | 6072 | 1.224e-67 |
| *Enterococcus* spp. | 0.26 (-0.04, 0.49) | 1.000 | 1.973 | 3625 | 372 | 2.726e-27 |
| *K. pneumoniae* | 0.18 (0.03, 0.34) | 0.9994 | 1.386 | 24335 | 24400 | 0 |
| *Morganella* spp. | 0.14 (0.01, 0.30) | 1.000 | 1.048 | 3859 | 3911 | 1.913e-26 |
| *P. aeruginosa* | 0.28 (0.13, 0.38) | 1.000 | 0.9221 | 10896 | 10959 | 9.961e-6 |
| *S. agalactiae* | 0.19 (-0.01, 1.38) | 1.000 | 1.495 | 5071 | 5124 | 1.318e-10 |
| *S. aureus* | 0.29 (0.02, 0.55) | 1.000 | 0.9744 | 10056 | 10116 | 7.084e-3 |
| *S. pneumoniae* | 0.40 (0.11, 0.66) | 1.000 | 1.196 | 12934 | 12995 | 1.283e-35 |
