# Supplementary material for: Burden of bacterial antimicrobial resistance associated with modifiable human antibiotic consumption: An observational study
Source: PLoS Med. 2026 Oct 26;23(10):e1005140. doi: 10.1371/journal.pmed.1005140 (PMC13719055; doi:10.1371/journal.pmed.1005140)
Supplement: Supplementary file 36 [file pmed.1005140.s013.docx]

**Table S11: Measures of model fit for each regression at the antibiotic class level, along with *p*-values of the White test for homoskedasticity.**

| **Antibiotic class** | **Elasticity** | ***R*^2^** | **RMSE** | **AIC** | **BIC** | **White *p*-value** |
| --- | --- | --- | --- | --- | --- | --- |
| Tetracyclines | 0.02 (-0.07, 0.15) | 1.000 | 1.323 | 21873 | 21939 | 1.833e-125 |
| Penicillins | 0.29 (0.06, 0.69) | 1.000 | 1.161 | 27852 | 27920 | 3.502e-50 |
| Non-penicillin beta-lactams | 0.23 (0.02, 0.42) | 0.9984 | 1.393 | 45733 | 45802 | 1.550e-48 |
| Sulfonamides and trimethoprim | 0.09 (-0.19, 0.31) | 0.4192 | 1.491 | 1897 | 1930 | 7.753e-2 |
| Macrolides | 0.43 (-0.02, 0.96) | 1.000 | 1.176 | 7022 | 7078 | 3.932e-19 |
| Aminoglycosides | 0.22 (-0.01, 0.40) | 1.000 | 1.097 | 15006 | 15130 | 1.583e-8 |
| Quinolones | 0.53 (0.33, 0.68) | 1.000 | 0.9791 | 30634 | 30703 | 4.261e-35 |
