# Supplementary material for: Burden of bacterial antimicrobial resistance associated with modifiable human antibiotic consumption: An observational study
Source: PLoS Med. 2026 Oct 26;23(10):e1005140. doi: 10.1371/journal.pmed.1005140 (PMC13719055; doi:10.1371/journal.pmed.1005140)
Supplement: Supplementary file 37 [file pmed.1005140.s014.docx]

**Table S12: Influential observations with Cook’s distance greater than 1.**

| **Pathogen** | **Antibiotic** | **ISO3** | **Year** | **Cook’s distance** | **Consumption (DDD/1000 person-days)** | **Percent resistant isolates** | **Total isolates** |
| --- | --- | --- | --- | --- | --- | --- | --- |
| *K. pneumoniae* | Quinolones | JPN | 2017 | 11.084 | 2.498 | 3.134 | 154198 |
| *E. coli* | Non-Penicillin Beta-Lactams | JPN | 2017 | 9.248 | 4.082 | 0.052 | 893393 |
| *K. pneumoniae* | Quinolones | JPN | 2018 | 8.769 | 2.366 | 3.006 | 144534 |
| *E. coli* | Non-Penicillin Beta-Lactams | JPN | 2018 | 7.836 | 3.729 | 0.033 | 797606 |
| *K. pneumoniae* | Non-Penicillin Beta-Lactams | JPN | 2017 | 6.854 | 4.082 | 0.169 | 203309 |
| *K. pneumoniae* | Non-Penicillin Beta-Lactams | JPN | 2018 | 5.398 | 3.729 | 0.162 | 183788 |
| *E. coli* | Non-Penicillin Beta-Lactams | GBR | 2018 | 4.985 | 0.557 | 0.083 | 741888 |
| *E. coli* | Quinolones | JPN | 2017 | 4.880 | 2.498 | 36.304 | 685441 |
| *Acinetobacter* spp. | Non-Penicillin Beta-Lactams | JPN | 2018 | 3.807 | 3.729 | 0.897 | 2786 |
| *E. coli* | Non-Penicillin Beta-Lactams | JPN | 2017 | 3.653 | 4.082 | 22.413 | 690885 |
| *E. coli* | Non-Penicillin Beta-Lactams | GBR | 2017 | 3.446 | 0.570 | 0.097 | 689419 |
| *Acinetobacter* spp. | Tetracyclines | JPN | 2018 | 3.170 | 0.957 | 0.240 | 1665 |
| *E. coli* | Non-Penicillin Beta-Lactams | JPN | 2018 | 2.685 | 3.729 | 21.312 | 613375 |
| *Acinetobacter* spp | Aminoglycosides | JPN | 2018 | 2.288 | 0.041 | 0.688 | 1745 |
| *Acinetobacter* spp | Sulfonamides and Trimethoprim | USA | 2018 | 2.080 | 1.692 | 39.570 | 139 |
| *Acinetobacter* spp | Non-Penicillin Beta-Lactams | JPN | 2017 | 1.919 | 4.082 | 1.130 | 2123 |
| *E. coli* | Sulfonamides and Trimethoprim | SWE | 2018 | 1.836 | 0.414 | 20.945 | 103223 |
| *Acinetobacter* spp | Tetracyclines | JPN | 2017 | 1.818 | 0.881 | 0.078 | 1283 |
| *E. coli* | Penicillins | PRI | 2006 | 1.813 | 5.159 | 65.926 | 25 |
| *K. pneumoniae* | Quinolones | GBR | 2018 | 1.621 | 0.672 | 14.744 | 62200 |
| *Salmonella* spp. | Non-Penicillin Beta-Lactams | PAK | 2018 | 1.536 | 3.459 | 0.122 | 3273 |
| *K. pneumoniae* | Non-Penicillin Beta-Lactams | JPN | 2017 | 1.506 | 4.082 | 8.554 | 157533 |
| *S. pneumoniae* | Non-Penicillin Beta-Lactams | JPN | 2017 | 1.450 | 4.082 | 0.442 | 7914 |
| *Salmonella* spp. | Quinolones | PAK | 2018 | 1.285 | 4.945 | 68.139 | 3917 |
| *K. pneumoniae* | Non-Penicillin Beta-Lactams | GBR | 2018 | 1.268 | 0.557 | 1.006 | 79525 |
| *E. coli* | Quinolones | GBR | 2018 | 1.244 | 0.672 | 11.351 | 922877 |
| *K. pneumoniae* | Non-Penicillin Beta-Lactams | JPN | 2018 | 1.207 | 3.729 | 7.918 | 140356 |
| *K. pneumoniae* | Non-Penicillin Beta-Lactams | GBR | 2017 | 1.174 | 0.570 | 0.816 | 73676 |
| *E. coli* | Sulfonamides and Trimethoprim | FIN | 2017 | 1.171 | 1.027 | 15.125 | 45323 |
| *E. faecium* | Penicillins | CHN | 2014 | 1.169 | 1.992 | 0.000 | 30 |
| *Acinetobacter* spp | Aminoglycosides | JPN | 2017 | 1.091 | 0.045 | 0.949 | 1370 |
