# Supplementary material for: Burden of bacterial antimicrobial resistance associated with modifiable human antibiotic consumption: An observational study
Source: PLoS Med. 2026 Oct 26;23(10):e1005140. doi: 10.1371/journal.pmed.1005140 (PMC13719055; doi:10.1371/journal.pmed.1005140)
Supplement: Supplementary file 38 [file pmed.1005140.s015.docx]

**Table S13: Type II ANOVA measure of variance explained by each variable for each regression at the antibiotic class-pathogen level.**

| **Antibiotic class** | **Pathogen** | **Consumption** | **PC1** | **PC2** | **PC3** | **GDP** | **Year** |
| --- | --- | --- | --- | --- | --- | --- | --- |
| Tetracyclines | *Acinetobacter* spp. | 0.01088 | 0.1357 | 0.1869 | 0.5714 | 0.01651 | 0.07864 |
|  | *E. coli* | 0.00892 | 5.704e-05 | 0.0001054 | 0.0001983 | 0.001047 | 0.9897 |
|  | *E. faecalis* | 0.003176 | 0.001378 | 0.004882 | 0.001182 | 0.06024 | 0.9291 |
|  | *E. faecium* | 0.05411 | 0.00188 | 0.00912 | 0.008693 | 0.1376 | 0.7886 |
|  | *Enterococcus* spp. | 0.007582 | 0.01626 | 0.02193 | 0.01439 | 0.1023 | 0.8376 |
|  | *H. influenzae* | 0.03169 | 0.0001498 | 0.01035 | 0.09886 | 0.01223 | 0.8467 |
|  | *K. pneumoniae* | 0.006556 | 0.0003498 | 0.002303 | 0.0001618 | 0.00383 | 0.9868 |
|  | *Morganella* spp. | 0.02266 | 0.1235 | 0.09807 | 0.1497 | 0.06558 | 0.5405 |
|  | *S. agalactiae* | 0.004098 | 0.01298 | 0.03763 | 0.02481 | 0.005524 | 0.915 |
|  | *S. aureus* | 0.1059 | 0.09461 | 0.001257 | 0.001138 | 0.7789 | 0.01824 |
|  | *S. pyogenes* | 0.005468 | 0.2573 | 0.07082 | 0.01154 | 0.03829 | 0.6166 |
| Penicillins | *Acinetobacter* spp. | 0.188 | 0.0226 | 0.04858 | 0.009002 | 0.4957 | 0.2361 |
|  | *E. coli* | 0.02103 | 0.08561 | 0.1254 | 0.02693 | 0.5714 | 0.1696 |
|  | *E. faecalis* | 0.01131 | 0.02076 | 0.01719 | 0.008252 | 0.1659 | 0.7766 |
|  | *E. faecium* | 0.1489 | 0.0198 | 0.01989 | 0.008942 | 0.2254 | 0.5771 |
|  | *Enterococcus* spp. | 0.02054 | 0.01723 | 0.04574 | 0.05019 | 0.005828 | 0.8605 |
|  | *H. influenzae* | 0.1773 | 0.1206 | 0.02093 | 0.06507 | 0.4867 | 0.1294 |
|  | *K. pneumoniae* | 0.0002241 | 0.003382 | 0.00444 | 0.01683 | 0.3024 | 0.6728 |
|  | *Morganella* spp. | 0.04843 | 0.3664 | 0.3556 | 0.1681 | 0.001182 | 0.06019 |
|  | *P. aeruginosa* | 0.005284 | 0.05849 | 0.02087 | 0.2071 | 0.681 | 0.02726 |
|  | *S. aureus* | 0.3035 | 0.00191 | 0.00738 | 0.04255 | 0.6283 | 0.01643 |
|  | *S. pneumoniae* | 0.1076 | 0.168 | 0.2846 | 0.2191 | 0.02752 | 0.1932 |
| Non-penicillin beta-lactams | *Acinetobacter* spp. | 0.1395 | 0.01276 | 0.01923 | 0.05538 | 0.4528 | 0.3204 |
|  | *E. coli* | 0.4504 | 0.02617 | 0.009322 | 0.008544 | 0.3292 | 0.1763 |
|  | *K. pneumoniae* | 0.03731 | 0.001284 | 0.004767 | 0.5332 | 0.4085 | 0.01496 |
|  | *Morganella* spp. | 0.2377 | 0.01434 | 0.00361 | 0.07873 | 0.3373 | 0.3283 |
|  | *P. aeruginosa* | 0.4753 | 0.0004064 | 0.0189 | 0.002365 | 0.4761 | 0.02696 |
|  | *S. aureus* | 0.03962 | 0.07542 | 0.1688 | 0.001066 | 0.6845 | 0.03054 |
|  | *S. pneumoniae* | 0.1182 | 0.0411 | 0.1486 | 0.6742 | 7.546e-06 | 0.01782 |
|  | *Salmonella* spp. | 0.2499 | 0.04626 | 0.139 | 0.08128 | 0.06273 | 0.4209 |
| Sulfonamides and trimethoprim | *Acinetobacter* spp. | 0.06039 | 0.1971 | 0.1188 | 0.0405 | 0.5832 | — |
|  | *E. coli* | 7.758e-05 | 0.06726 | 0.01408 | 0.05823 | 0.7626 | 0.09779 |
|  | *K. pneumoniae* | 0.03254 | 0.1632 | 0.1594 | 0.1828 | 0.4552 | 0.006782 |
|  | *Morganella* spp. | 0.08949 | 0.05753 | 0.2865 | 0.4647 | 0.1017 | — |
|  | *S. aureus* | 0.1389 | 0.5486 | 0.0793 | 0.02 | 0.03613 | 0.177 |
| Macrolides | *E. faecalis* | 0.0203 | 0.0003499 | 0.0001514 | 0.001368 | 0.01311 | 0.9647 |
|  | *E. faecium* | 0.09229 | 5.817e-05 | 0.0171 | 0.1355 | 0.01496 | 0.7401 |
|  | *S. agalactiae* | 0.05584 | 0.1816 | 0.4886 | 0.03706 | 0.0136 | 0.2233 |
|  | *S. aureus* | 0.09684 | 0.2576 | 0.3549 | 0.03746 | 0.07722 | 0.1759 |
|  | *S. pneumoniae* | 0.5318 | 0.1184 | 0.1504 | 0.1313 | 0.03873 | 0.02938 |
|  | *S. pyogenes* | 0.0786 | 0.2047 | 0.5256 | 0.1367 | 0.05186 | 0.002462 |
| Aminoglycosides | *Acinetobacter* spp. | 0.03946 | 0.05849 | 0.02217 | 0.09629 | 0.783 | 0.0005904 |
|  | *E. coli* | 0.2108 | 0.07479 | 0.004287 | 0.02307 | 0.2363 | 0.4507 |
|  | *E. faecalis* | 0.05631 | 0.07712 | 0.01481 | 0.05994 | 0.4932 | 0.2986 |
|  | *E. faecium* | 0.4483 | 0.006205 | 0.0712 | 0.1832 | 0.1007 | 0.1904 |
|  | *K. pneumoniae* | 0.2139 | 0.01962 | 0.01338 | 0.07164 | 0.6648 | 0.01668 |
|  | *Morganella* spp. | 0.1335 | 0.1183 | 0.01923 | 0.0686 | 0.06478 | 0.5956 |
|  | *P. aeruginosa* | 0.0606 | 0.05768 | 0.001723 | 0.05242 | 0.7966 | 0.031 |
|  | *S. aureus* | 0.02101 | 0.3232 | 0.1987 | 0.02492 | 0.3528 | 0.07939 |
| Quinolones | *Acinetobacter* spp. | 0.6249 | 0.004741 | 0.007769 | 0.002577 | 0.36 | 2.206e-05 |
|  | *E. coli* | 0.8437 | 0.0001687 | 0.0009964 | 0.004901 | 0.07106 | 0.07922 |
|  | *E. faecalis* | 0.7676 | 0.001291 | 0.001733 | 0.00483 | 0.05429 | 0.1702 |
|  | *E. faecium* | 0.01672 | 0.05088 | 0.2468 | 0.3182 | 0.3671 | 0.0003536 |
|  | *Enterococcus* spp. | 0.2628 | 0.08284 | 0.0683 | 0.001358 | 0.0561 | 0.5286 |
|  | *K. pneumoniae* | 0.01687 | 0.007765 | 0.0001328 | 0.8688 | 0.05968 | 0.04675 |
|  | *Morganella* spp. | 0.5477 | 0.1799 | 0.01243 | 0.03186 | 0.01764 | 0.2105 |
|  | *P. aeruginosa* | 0.7376 | 0.004891 | 0.0004801 | 0.01219 | 0.2263 | 0.01849 |
|  | *S. agalactiae* | 0.1902 | 0.0001677 | 0.001762 | 0.002725 | 0.002468 | 0.8027 |
|  | *S. aureus* | 0.8608 | 0.003949 | 0.06848 | 0.03004 | 0.03664 | 6.424e-05 |
|  | *S. pneumoniae* | 0.5659 | 0.1135 | 0.09044 | 0.09025 | 0.07167 | 0.06824 |
|  | *S. pyogenes* | 0.1117 | 0.07463 | 0.004673 | 0.3915 | 0.2194 | 0.1981 |
|  | *Salmonella* spp. | 0.6201 | 0.2192 | 0.124 | 0.008843 | 0.02752 | 0.0003419 |
