# Supplementary material for: Burden of bacterial antimicrobial resistance associated with modifiable human antibiotic consumption: An observational study
Source: PLoS Med. 2026 Oct 26;23(10):e1005140. doi: 10.1371/journal.pmed.1005140 (PMC13719055; doi:10.1371/journal.pmed.1005140)
Supplement: Supplementary file 39 [file pmed.1005140.s016.docx]

**Table S14: Variance inflation factor measure of collinearity by each variable for each regression at the antibiotic class-pathogen level.**

| **Antibiotic class** | **Pathogen** | **Consumption** | **PC1** | **PC2** | **PC3** | **GDP** | **Year** |
| --- | --- | --- | --- | --- | --- | --- | --- |
| Tetracyclines | *Acinetobacter* spp. | 2.036 | 18.1 | 17.31 | 1.62 | 2.805 | 1.299 |
|  | *E. coli* | 1.983 | 9.111 | 10.51 | 1.39 | 2.967 | 1.159 |
|  | *E. faecalis* | 2.057 | 10.02 | 11.77 | 1.495 | 3.1 | 1.185 |
|  | *E. faecium* | 1.97 | 9.377 | 10.54 | 1.271 | 2.697 | 1.192 |
|  | *Enterococcus* spp. | 1.822 | 23.76 | 28.17 | 1.565 | 3.45 | 1.228 |
|  | *H. influenzae* | 1.916 | 14.29 | 15.97 | 1.32 | 2.453 | 1.244 |
|  | *K. pneumoniae* | 1.981 | 8.751 | 9.987 | 1.431 | 2.937 | 1.167 |
|  | *Morganella* spp. | 1.767 | 10.1 | 11.35 | 1.134 | 2.502 | 1.029 |
|  | *S. agalactiae* | 1.933 | 8.997 | 10.14 | 1.548 | 2.66 | 1.184 |
|  | *S. aureus* | 1.978 | 8.102 | 9.278 | 1.282 | 2.804 | 1.135 |
|  | *S. pyogenes* | 1.624 | 9.849 | 11.95 | 1.124 | 2.408 | 1.019 |
| Penicillins | *Acinetobacter* spp. | 1.626 | 10.2 | 11.06 | 1.279 | 1.948 | 1.178 |
|  | *E. coli* | 1.482 | 8.523 | 9.715 | 1.365 | 2.027 | 1.132 |
|  | *E. faecalis* | 1.558 | 10.66 | 12.28 | 1.496 | 1.932 | 1.159 |
|  | *E. faecium* | 1.338 | 9.882 | 10.91 | 1.257 | 1.769 | 1.155 |
|  | *Enterococcus* spp. | 2.343 | 24.34 | 27.75 | 1.523 | 2.659 | 1.225 |
|  | *H. influenzae* | 1.277 | 13.76 | 15.4 | 1.298 | 1.722 | 1.212 |
|  | *K. pneumoniae* | 1.407 | 5.791 | 6.118 | 1.314 | 1.713 | 1.253 |
|  | *Morganella* spp. | 1.321 | 10.17 | 11.18 | 1.119 | 1.924 | 1.027 |
|  | *P. aeruginosa* | 1.264 | 7.009 | 7.376 | 1.292 | 1.453 | 1.104 |
|  | *S. aureus* | 1.142 | 4.732 | 5.182 | 1.26 | 1.367 | 1.252 |
|  | *S. pneumoniae* | 1.084 | 7.264 | 7.773 | 1.332 | 1.464 | 1.169 |
| Non-penicillin beta-lactams | *Acinetobacter* spp. | 1.48 | 14.19 | 15.33 | 1.624 | 1.884 | 1.186 |
|  | *E. coli* | 1.57 | 37.74 | 34.33 | 2.667 | 1.709 | 1.075 |
|  | *K. pneumoniae* | 1.358 | 33.61 | 32.85 | 2.409 | 1.458 | 1.034 |
|  | *Morganella* spp. | 1.272 | 10.16 | 11.12 | 1.281 | 1.777 | 1.029 |
|  | *P. aeruginosa* | 1.335 | 5.569 | 5.974 | 1.392 | 1.366 | 1.228 |
|  | *S. aureus* | 1.252 | 9.6 | 10.57 | 1.264 | 1.75 | 1.027 |
|  | *S. pneumoniae* | 1.215 | 19.17 | 19.58 | 1.466 | 1.547 | 1.422 |
|  | *Salmonella* spp. | 1.325 | 66.76 | 69.94 | 1.581 | 2.046 | 1.02 |
| Sulfonamides and trimethoprim | *Acinetobacter* spp. | 1.161 | 10.07 | 10.72 | 1.096 | 1.746 | — |
|  | *E. coli* | 2.031 | 18.4 | 25.33 | 2.464 | 3.951 | 1.14 |
|  | *K. pneumoniae* | 2.075 | 21.74 | 25.2 | 2.029 | 2.786 | 1.079 |
|  | *Morganella* spp. | 1.35 | 11.07 | 12.13 | 1.231 | 2.057 | — |
|  | *S. aureus* | 1.287 | 9.783 | 10.97 | 1.103 | 1.849 | 1.015 |
| Macrolides | *E. faecalis* | 1.788 | 9.684 | 11.71 | 1.162 | 1.818 | 1.128 |
|  | *E. faecium* | 1.541 | 7.66 | 8.909 | 1.213 | 1.692 | 1.088 |
|  | *S. agalactiae* | 1.912 | 9.601 | 11.98 | 1.155 | 1.837 | 1.055 |
|  | *S. aureus* | 1.973 | 9.356 | 10.98 | 1.181 | 1.776 | 1.056 |
|  | *S. pneumoniae* | 1.112 | 5.316 | 5.819 | 1.315 | 1.46 | 1.231 |
|  | *S. pyogenes* | 1.782 | 8.681 | 10.92 | 1.169 | 1.799 | 1.053 |
| Aminoglycosides | *Acinetobacter* spp. | 1.367 | 13.77 | 15.49 | 1.429 | 1.822 | 1.208 |
|  | *E. coli* | 1.129 | 4.071 | 4.704 | 1.204 | 1.35 | 1.302 |
|  | *E. faecalis* | 1.126 | 3.102 | 3.381 | 1.658 | 1.358 | 1.494 |
|  | *E. faecium* | 1.216 | 3.705 | 4.21 | 1.298 | 1.321 | 1.484 |
|  | *K. pneumoniae* | 1.065 | 4.655 | 5.138 | 1.325 | 1.416 | 1.143 |
|  | *Morganella* spp. | 1.067 | 9.735 | 10.96 | 1.13 | 1.768 | 1.029 |
|  | *P. aeruginosa* | 1.034 | 5.33 | 5.885 | 1.238 | 1.375 | 1.123 |
|  | *S. aureus* | 1.109 | 8.778 | 10.01 | 1.118 | 1.74 | 1.019 |
| Quinolones | *Acinetobacter* spp. | 1.324 | 6.522 | 7.155 | 1.631 | 1.455 | 1.412 |
|  | *E. coli* | 1.93 | 26.52 | 23.56 | 2.01 | 1.577 | 1.119 |
|  | *E. faecalis* | 1.351 | 9.932 | 11.76 | 1.545 | 1.989 | 1.216 |
|  | *E. faecium* | 1.181 | 8.997 | 10.43 | 1.289 | 1.735 | 1.218 |
|  | *Enterococcus* spp. | 1.144 | 22.87 | 28.21 | 1.565 | 2.495 | 1.246 |
|  | *K. pneumoniae* | 1.645 | 21.2 | 19.8 | 1.941 | 1.429 | 1.081 |
|  | *Morganella* spp. | 1.237 | 9.869 | 10.96 | 1.187 | 1.786 | 1.031 |
|  | *P. aeruginosa* | 1.448 | 5.579 | 5.834 | 1.351 | 1.49 | 1.209 |
|  | *S. agalactiae* | 1.235 | 8.853 | 10.13 | 1.595 | 1.763 | 1.201 |
|  | *S. aureus* | 1.292 | 8.754 | 10.08 | 1.351 | 1.804 | 1.152 |
|  | *S. pneumoniae* | 1.307 | 10.62 | 12.57 | 1.272 | 1.716 | 1.246 |
|  | *S. pyogenes* | 1.386 | 9.47 | 11.6 | 1.218 | 1.915 | 1.031 |
|  | *Salmonella* spp. | 1.117 | 58.84 | 55.72 | 2.003 | 2.805 | 1.084 |
