# Supplementary material for: Burden of bacterial antimicrobial resistance associated with modifiable human antibiotic consumption: An observational study
Source: PLoS Med. 2026 Oct 26;23(10):e1005140. doi: 10.1371/journal.pmed.1005140 (PMC13719055; doi:10.1371/journal.pmed.1005140)
Supplement: Supplementary file 40 [file pmed.1005140.s017.docx]

**Table S15: Elasticity, variance inflation factor measure of collinearity, and type II ANOVA measure of variance explained by each variable for each regression at the antibiotic class-pathogen level, with PC2 and PC3 excluded.**

| **Antibiotic class** | **Pathogen** | **Elasticity (95% CI)** | **Variance inflation factor** | | | | **Variance explained** | | | |
| --- | --- | --- | --- | --- | --- | --- | --- | --- | --- | --- |
|  |  |  | Consumption | PC1 | GDP | Year | Consumption | PC1 | GDP | Year |
| Tetracyclines | *Acinetobacter* spp. | 0.14 (-0.26, 0.80) | 1.932 | 1.749 | 2.624 | 1.235 | 0.086 | 0.468 | 0.012 | 0.434 |
|  | *E. coli* | -0.11 (-0.22, 0.08) | 1.926 | 1.499 | 2.442 | 1.068 | 0.009 | 0.000 | 0.001 | 0.991 |
|  | *E. faecalis* | -0.05 (-0.20, 0.13) | 1.991 | 1.525 | 2.499 | 1.076 | 0.003 | 0.060 | 0.052 | 0.885 |
|  | *E. faecium* | 0.18 (-0.07, 0.37) | 1.891 | 1.413 | 2.207 | 1.071 | 0.070 | 0.011 | 0.162 | 0.758 |
|  | *Enterococcus* spp. | 0.07 (-0.32, 0.31) | 1.701 | 1.919 | 2.668 | 1.062 | 0.009 | 0.003 | 0.076 | 0.912 |
|  | *H. influenzae* | 0.03 (-0.05, 0.07) | 1.786 | 1.441 | 1.994 | 1.066 | 0.087 | 0.068 | 0.031 | 0.813 |
|  | *K. pneumoniae* | -0.09 (-0.21, 0.08) | 1.922 | 1.507 | 2.458 | 1.079 | 0.006 | 0.003 | 0.002 | 0.989 |
|  | *Morganella* spp. | 0.10 (-0.22, 0.40) | 1.675 | 1.451 | 2.212 | 1.003 | 0.121 | 0.041 | 0.085 | 0.753 |
|  | *S. agalactiae* | 0.05 (-0.08, 0.23) | 1.888 | 1.417 | 2.153 | 1.080 | 0.004 | 0.029 | 0.033 | 0.935 |
|  | *S. aureus* | 0.09 (-0.19, 0.31) | 1.910 | 1.413 | 2.395 | 1.068 | 0.073 | 0.287 | 0.628 | 0.013 |
|  | *S. pyogenes* | -0.03 (-0.14, 0.13) | 1.510 | 1.337 | 1.918 | 1.003 | 0.018 | 0.418 | 0.016 | 0.548 |
| Penicillins | *Acinetobacter* spp. | 0.34 (0.03, 0.60) | 1.602 | 1.782 | 1.507 | 1.106 | 0.160 | 0.012 | 0.656 | 0.172 |
|  | *E. coli* | 0.02 (-0.04, 0.09) | 1.467 | 1.624 | 1.469 | 1.060 | 0.020 | 0.003 | 0.881 | 0.097 |
|  | *E. faecalis* | -0.03 (-0.27, 0.21) | 1.538 | 1.665 | 1.307 | 1.058 | 0.008 | 0.299 | 0.107 | 0.586 |
|  | *E. faecium* | 0.03 (-0.02, 0.11) | 1.320 | 1.440 | 1.205 | 1.050 | 0.128 | 0.002 | 0.374 | 0.495 |
|  | *Enterococcus* spp. | -0.24 (-0.81, 0.68) | 2.302 | 2.434 | 1.638 | 1.069 | 0.042 | 0.047 | 0.001 | 0.909 |
|  | *H. influenzae* | 0.19 (-0.17, 0.45) | 1.257 | 1.401 | 1.167 | 1.062 | 0.098 | 0.240 | 0.572 | 0.090 |
|  | *K. pneumoniae* | 0.05 (-0.11, 0.27) | 1.308 | 1.659 | 1.273 | 1.159 | 0.002 | 0.001 | 0.408 | 0.589 |
|  | *Morganella* spp. | 0.00 (-0.00, 0.00) | 1.290 | 1.531 | 1.526 | 1.003 | 0.601 | 0.161 | 0.077 | 0.161 |
|  | *P. aeruginosa* | 0.06 (-0.22, 0.36) | 1.259 | 1.383 | 1.087 | 1.041 | 0.007 | 0.022 | 0.940 | 0.030 |
|  | *S. aureus* | 0.71 (0.14, 1.55) | 1.075 | 1.126 | 1.046 | 1.062 | 0.239 | 0.001 | 0.757 | 0.003 |
|  | *S. pneumoniae* | 0.46 (-0.29, 1.03) | 1.010 | 1.047 | 1.007 | 1.049 | 0.275 | 0.109 | 0.445 | 0.170 |
| Non-penicillin beta-lactams | *Acinetobacter* spp. | 0.40 (0.02, 0.74) | 1.140 | 1.788 | 1.808 | 1.155 | 0.271 | 0.017 | 0.463 | 0.249 |
|  | *E. coli* | 0.26 (0.09, 0.35) | 1.405 | 1.334 | 1.145 | 1.031 | 0.384 | 0.063 | 0.373 | 0.179 |
|  | *K. pneumoniae* | 0.19 (-0.06, 0.55) | 1.232 | 1.287 | 1.233 | 1.023 | 0.064 | 0.746 | 0.174 | 0.017 |
|  | *Morganella* spp. | 0.02 (-0.05, 0.06) | 1.097 | 1.557 | 1.440 | 1.003 | 0.195 | 0.022 | 0.346 | 0.437 |
|  | *P. aeruginosa* | 0.36 (0.14, 0.54) | 1.158 | 1.202 | 1.121 | 1.115 | 0.426 | 0.039 | 0.516 | 0.018 |
|  | *S. aureus* | -0.02 (-0.07, 0.02) | 1.094 | 1.460 | 1.360 | 1.007 | 0.096 | 0.093 | 0.769 | 0.042 |
|  | *S. pneumoniae* | 0.24 (-0.11, 0.61) | 1.202 | 1.554 | 1.114 | 1.327 | 0.150 | 0.779 | 0.031 | 0.040 |
|  | *Salmonella* spp. | -0.18 (-0.46, 0.28) | 1.206 | 1.475 | 1.669 | 1.013 | 0.157 | 0.545 | 0.003 | 0.295 |
| Sulfonamides and trimethoprim | *Acinetobacter* spp. | 0.07 (-0.15, 0.27) | 1.106 | 1.476 | 1.605 | — | 0.030 | 0.108 | 0.863 | — |
|  | *E. coli* | -0.01 (-0.35, 0.11) | 1.639 | 3.166 | 2.359 | 1.082 | 0.003 | 0.419 | 0.527 | 0.050 |
|  | *K. pneumoniae* | 0.00 (-0.53, 0.18) | 1.749 | 3.215 | 2.181 | 1.057 | 0.000 | 0.021 | 0.967 | 0.012 |
|  | *Morganella* spp. | -0.20 (-0.44, 0.17) | 1.298 | 1.443 | 1.774 | — | 0.243 | 0.752 | 0.005 | — |
|  | *S. aureus* | -0.07 (-0.29, 0.48) | 1.174 | 1.426 | 1.607 | 1.007 | 0.047 | 0.805 | 0.053 | 0.095 |
| Macrolides | *E. faecalis* | 0.09 (-0.01, 0.21) | 1.633 | 1.833 | 1.414 | 1.064 | 0.018 | 0.004 | 0.015 | 0.964 |
|  | *E. faecium* | 0.06 (-0.03, 0.12) | 1.440 | 1.581 | 1.296 | 1.048 | 0.195 | 0.045 | 0.043 | 0.717 |
|  | *S. agalactiae* | -0.03 (-0.71, 0.63) | 1.673 | 2.052 | 1.456 | 1.040 | 0.002 | 0.250 | 0.301 | 0.447 |
|  | *S. aureus* | 0.30 (-0.11, 0.61) | 1.783 | 1.958 | 1.416 | 1.029 | 0.623 | 0.000 | 0.042 | 0.335 |
|  | *S. pneumoniae* | 0.72 (0.13, 1.29) | 1.061 | 1.045 | 1.046 | 1.058 | 0.641 | 0.004 | 0.293 | 0.062 |
|  | *S. pyogenes* | -0.05 (-1.02, 0.72) | 1.588 | 1.759 | 1.336 | 1.025 | 0.024 | 0.963 | 0.010 | 0.004 |
| Aminoglycosides | *Acinetobacter* spp. | 0.21 (-0.25, 0.65) | 1.219 | 1.801 | 1.724 | 1.151 | 0.074 | 0.039 | 0.886 | 0.001 |
|  | *E. coli* | 0.21 (0.01, 0.34) | 1.077 | 1.075 | 1.079 | 1.099 | 0.177 | 0.120 | 0.293 | 0.409 |
|  | *E. faecalis* | 0.08 (-0.34, 0.43) | 1.082 | 1.129 | 1.101 | 1.148 | 0.047 | 0.179 | 0.590 | 0.184 |
|  | *E. faecium* | 0.36 (-0.10, 0.63) | 1.062 | 1.200 | 1.132 | 1.169 | 0.789 | 0.121 | 0.023 | 0.067 |
|  | *K. pneumoniae* | 0.44 (0.12, 0.73) | 1.054 | 1.106 | 1.099 | 1.063 | 0.153 | 0.131 | 0.685 | 0.031 |
|  | *Morganella* spp. | 0.31 (-0.07, 0.55) | 1.054 | 1.501 | 1.439 | 1.006 | 0.129 | 0.253 | 0.059 | 0.559 |
|  | *P. aeruginosa* | 0.23 (-0.05, 0.57) | 1.017 | 1.124 | 1.084 | 1.057 | 0.046 | 0.201 | 0.742 | 0.011 |
|  | *S. aureus* | 0.08 (-0.39, 0.49) | 1.093 | 1.467 | 1.375 | 1.009 | 0.018 | 0.174 | 0.715 | 0.093 |
| Quinolones | *Acinetobacter* spp. | 1.15 (0.58, 1.38) | 1.247 | 1.616 | 1.357 | 1.234 | 0.620 | 0.000 | 0.379 | 0.001 |
|  | *E. coli* | 0.69 (0.41, 0.82) | 1.652 | 1.483 | 1.196 | 1.077 | 0.830 | 0.000 | 0.100 | 0.070 |
|  | *E. faecalis* | 0.43 (0.19, 0.62) | 1.264 | 1.441 | 1.378 | 1.094 | 0.697 | 0.025 | 0.079 | 0.199 |
|  | *E. faecium* | 0.02 (-0.07, 0.09) | 1.136 | 1.312 | 1.230 | 1.097 | 0.073 | 0.203 | 0.640 | 0.084 |
|  | *Enterococcus* spp. | 0.96 (-0.04, 1.46) | 1.065 | 1.673 | 1.636 | 1.070 | 0.439 | 0.031 | 0.021 | 0.509 |
|  | *K. pneumoniae* | 0.37 (0.03, 0.90) | 1.472 | 1.507 | 1.230 | 1.080 | 0.110 | 0.839 | 0.022 | 0.029 |
|  | *Morganella* spp. | 0.59 (0.21, 0.94) | 1.162 | 1.665 | 1.477 | 1.004 | 0.419 | 0.409 | 0.013 | 0.159 |
|  | *P. aeruginosa* | 0.66 (0.33, 0.81) | 1.312 | 1.338 | 1.253 | 1.105 | 0.707 | 0.038 | 0.244 | 0.011 |
|  | *S. agalactiae* | 0.30 (-0.04, 0.54) | 1.165 | 1.286 | 1.245 | 1.088 | 0.178 | 0.012 | 0.006 | 0.805 |
|  | *S. aureus* | 0.39 (0.00, 0.71) | 1.197 | 1.459 | 1.346 | 1.085 | 0.876 | 0.104 | 0.011 | 0.009 |
|  | *S. pneumoniae* | 0.22 (-0.05, 0.36) | 1.176 | 1.300 | 1.216 | 1.116 | 0.607 | 0.089 | 0.287 | 0.018 |
|  | *S. pyogenes* | 0.02 (-0.06, 0.10) | 1.223 | 1.587 | 1.343 | 1.008 | 0.021 | 0.501 | 0.316 | 0.162 |
|  | *Salmonella* spp. | 0.81 (-0.60, 1.26) | 1.090 | 2.274 | 2.300 | 1.080 | 0.602 | 0.354 | 0.044 | 0.000 |
