# Supplementary material for: Burden of bacterial antimicrobial resistance associated with modifiable human antibiotic consumption: An observational study
Source: PLoS Med. 2026 Oct 26;23(10):e1005140. doi: 10.1371/journal.pmed.1005140 (PMC13719055; doi:10.1371/journal.pmed.1005140)
Supplement: Supplementary file 41 [file pmed.1005140.s018.docx]

**Table S16: Elasticity estimates from each regression at the antibiotic class-pathogen level, under various sensitivity analyses.**

| Antibiotic | Pathogen | Scaled to 2000 | 1y lag | 3y lag | 5y lag | Extra variables | Removed influential | Lower continuity |
| --- | --- | --- | --- | --- | --- | --- | --- | --- |
| Aminoglycosides | *Acinetobacter* spp. | 0.15 (-0.26, 0.52) | 0.16 (-0.25, 0.51) | 0.18 (-0.23, 0.53) | 0.17 (-0.20, 0.54) | 0.21 (-0.25, 0.48) | 0.03 (-0.34, 0.34) | 0.20 (-0.28, 0.65) |
|  | *E. coli* | 0.21 (-0.01, 0.32) | 0.20 (-0.02, 0.32) | 0.20 (-0.02, 0.32) | 0.19 (-0.02, 0.31) | 0.20 (0.01, 0.30) | 0.21 (-0.01, 0.32) | 0.26 (-0.01, 0.39) |
|  | *E. faecalis* | 0.08 (-0.33, 0.44) | 0.06 (-0.37, 0.43) | 0.03 (-0.44, 0.42) | -0.03 (-0.57, 0.44) | 0.06 (-0.13, 0.37) | 0.08 (-0.33, 0.44) | 0.09 (-0.34, 0.46) |
|  | *E. faecium* | 0.31 (-0.17, 0.69) | 0.31 (-0.21, 0.68) | 0.25 (-0.25, 0.60) | 0.28 (-0.23, 0.72) | 0.25 (-0.13, 0.65) | 0.31 (-0.17, 0.69) | 0.33 (-0.17, 0.71) |
|  | *K. pneumoniae* | 0.43 (0.12, 0.72) | 0.46 (0.15, 0.72) | 0.51 (0.20, 0.76) | 0.52 (0.20, 0.76) | 0.42 (0.09, 0.71) | 0.43 (0.12, 0.72) | 0.56 (0.19, 0.89) |
|  | *Morganella* spp. | 0.28 (-0.04, 0.55) | 0.22 (-0.11, 0.54) | 0.15 (-0.24, 0.54) | 0.11 (-0.43, 0.72) | 0.35 (-0.02, 0.56) | 0.28 (-0.04, 0.55) | 0.84 (-0.03, 1.53) |
|  | *P. aeruginosa* | 0.22 (-0.09, 0.56) | 0.24 (-0.07, 0.57) | 0.29 (-0.03, 0.60) | 0.32 (-0.01, 0.61) | 0.25 (-0.06, 0.55) | 0.22 (-0.09, 0.56) | 0.35 (-0.09, 0.83) |
|  | *S. aureus* | 0.09 (-0.34, 0.46) | 0.09 (-0.42, 0.51) | 0.15 (-0.34, 0.65) |  | 0.09 (-0.28, 0.51) | 0.09 (-0.34, 0.46) | 0.15 (-0.35, 0.61) |
| Macrolides | *E. faecalis* | 0.09 (0.01, 0.26) | 0.03 (-0.11, 0.18) | 0.06 (-0.16, 0.30) |  | 0.15 (0.05, 0.33) | 0.09 (0.01, 0.26) | 0.10 (0.01, 0.26) |
|  | *E. faecium* | 0.04 (-0.06, 0.11) | 0.02 (-0.12, 0.09) | -0.04 (-0.28, 0.10) |  | 0.04 (-0.06, 0.15) | 0.04 (-0.06, 0.11) | 0.06 (-0.04, 0.17) |
|  | *S. agalactiae* | -0.20 (-0.82, 0.61) | -0.27 (-0.87, 0.66) | 0.13 (-0.44, 0.92) | 0.57 (-0.33, 1.67) | 0.00 (-0.69, 0.81) | -0.20 (-0.82, 0.61) | -0.43 (-1.57, 1.14) |
|  | *S. aureus* | 0.20 (-0.17, 0.57) | 0.16 (-0.21, 0.51) | 0.15 (-0.20, 0.50) | 0.13 (-0.36, 0.60) | 0.30 (-0.10, 0.60) | 0.20 (-0.17, 0.57) | 0.20 (-0.17, 0.59) |
|  | *S. pneumoniae* | 0.68 (-0.03, 1.27) | 0.68 (-0.03, 1.26) | 0.69 (-0.01, 1.26) | 0.69 (0.00, 1.26) | 0.55 (-0.31, 1.02) | 0.68 (-0.03, 1.27) | 0.77 (-0.00, 1.42) |
|  | *S. pyogenes* | -0.26 (-1.03, 0.64) | -0.40 (-1.31, 0.52) | -0.61 (-1.72, 0.45) | -0.20 (-1.84, 0.99) | 0.13 (-0.58, 0.98) | -0.26 (-1.03, 0.64) | -0.65 (-2.22, 1.34) |
| Non-Penicillin Beta-Lactams | *Acinetobacter* spp. | 0.30 (-0.31, 0.73) | 0.28 (-0.37, 0.74) | 0.26 (-0.47, 0.73) | 0.28 (-0.56, 0.80) | 0.35 (-0.03, 0.75) | 0.42 (0.01, 0.81) | 0.39 (-0.27, 0.91) |
|  | *E. coli* | 0.28 (0.08, 0.38) | 0.28 (0.08, 0.40) | 0.30 (0.09, 0.54) | 0.27 (0.07, 0.40) | 0.16 (0.00, 0.31) | 0.01 (-0.31, 0.43) | 0.46 (0.12, 0.67) |
|  | *K. pneumoniae* | 0.10 (-0.18, 0.55) | 0.10 (-0.18, 0.54) | 0.11 (-0.15, 0.55) | 0.09 (-0.22, 0.54) | 0.24 (-0.16, 0.58) | 0.01 (-0.31, 0.47) | 0.12 (-0.27, 0.78) |
|  | *Morganella* spp. | 0.02 (-0.05, 0.07) | 0.02 (-0.04, 0.05) | 0.03 (-0.05, 0.08) | 0.03 (-0.07, 0.09) | 0.02 (-0.06, 0.10) | 0.02 (-0.05, 0.07) | 0.02 (-0.10, 0.08) |
|  | *P. aeruginosa* | 0.35 (0.13, 0.55) | 0.35 (0.13, 0.55) | 0.35 (0.11, 0.55) | 0.34 (0.11, 0.55) | 0.30 (0.09, 0.44) | 0.35 (0.13, 0.55) | 0.39 (0.14, 0.61) |
|  | *S. aureus* | -0.01 (-0.07, 0.02) | -0.02 (-0.10, 0.02) | -0.01 (-0.07, 0.04) | -0.05 (-0.20, 0.04) | -0.01 (-0.07, 0.05) | -0.01 (-0.07, 0.02) | -0.04 (-0.39, 0.17) |
|  | *S. pneumoniae* | 0.19 (-0.13, 0.61) | 0.17 (-0.16, 0.60) | 0.18 (-0.18, 0.60) | 0.15 (-0.24, 0.58) | 0.39 (0.08, 0.71) | 0.24 (-0.09, 0.62) | 0.96 (0.18, 1.63) |
|  | *Salmonella* spp. | -0.21 (-0.67, 0.42) | -0.16 (-1.04, 0.91) | -0.48 (-2.49, 1.61) | 0.29 (-5.86, 5.71) | -0.61 (-5.75, 2.47) | 0.05 (-0.32, 0.91) | -0.58 (-1.99, 0.96) |
| Penicillins | *Acinetobacter* spp. | 0.32 (0.02, 0.61) | 0.32 (0.01, 0.61) | 0.35 (0.02, 0.70) | 0.30 (-0.07, 0.65) | 0.34 (-0.02, 0.67) | 0.32 (0.02, 0.61) | 0.50 (0.11, 0.96) |
|  | *E. coli* | 0.02 (-0.04, 0.09) | 0.01 (-0.05, 0.09) | 0.01 (-0.05, 0.10) | -0.01 (-0.07, 0.09) | 0.05 (-0.01, 0.11) | 0.02 (-0.04, 0.09) | 0.02 (-0.04, 0.09) |
|  | *E. faecalis* | -0.03 (-0.28, 0.22) | -0.05 (-0.31, 0.22) | -0.06 (-0.35, 0.28) | -0.07 (-0.39, 0.28) | -0.02 (-0.29, 0.23) | -0.03 (-0.28, 0.22) | -0.05 (-1.20, 1.05) |
|  | *E. faecium* | 0.03 (-0.02, 0.11) | 0.04 (-0.03, 0.14) | 0.02 (-0.04, 0.08) | 0.02 (-0.05, 0.09) | 0.01 (-0.03, 0.07) | 0.00 (-0.03, 0.04) | 0.08 (-0.01, 0.23) |
|  | *Enterococcus* spp. | -0.19 (-0.80, 0.65) | -0.15 (-0.81, 0.80) | -0.14 (-1.32, 0.90) | -0.19 (-1.83, 1.26) | 0.34 (-0.55, 1.30) | -0.19 (-0.80, 0.65) | -0.20 (-1.55, 1.54) |
|  | *H. influenzae* | 0.21 (-0.14, 0.48) | 0.19 (-0.17, 0.48) | 0.17 (-0.21, 0.50) | 0.20 (-0.23, 0.63) | 0.29 (-0.06, 0.56) | 0.21 (-0.14, 0.48) | 0.41 (-0.10, 0.88) |
|  | *K. pneumoniae* | 0.01 (-0.15, 0.28) | -0.02 (-0.19, 0.25) | -0.07 (-0.31, 0.20) | -0.04 (-0.30, 0.24) | 0.05 (-0.16, 0.35) | 0.01 (-0.15, 0.28) | 0.02 (-0.16, 0.30) |
|  | *Morganella* spp. | 0.00 (-0.00, 0.00) | 0.00 (-0.00, 0.00) | 0.00 (-0.00, 0.00) | 0.00 (-0.00, 0.00) | -0.00 (-0.00, 0.00) | 0.00 (0.00, 0.00) | 0.00 (-0.00, 0.00) |
|  | *P. aeruginosa* | 0.04 (-0.23, 0.30) | 0.04 (-0.24, 0.31) | 0.02 (-0.27, 0.31) | 0.02 (-0.30, 0.32) | 0.07 (-0.29, 0.35) | 0.04 (-0.23, 0.30) | 0.06 (-0.25, 0.36) |
|  | *S. aureus* | 0.75 (0.16, 1.58) | 0.73 (0.15, 1.58) | 0.71 (0.14, 1.59) | 0.78 (0.22, 1.67) | 0.88 (0.26, 1.65) | 0.75 (0.16, 1.58) | 0.89 (0.17, 1.92) |
|  | *S. pneumoniae* | 0.32 (-0.31, 0.93) | 0.33 (-0.32, 0.94) | 0.34 (-0.32, 0.93) | 0.35 (-0.37, 0.98) | 0.27 (-0.26, 0.87) | 0.32 (-0.31, 0.93) | 0.58 (-0.60, 1.50) |
| Quinolones | *Acinetobacter* spp. | 1.16 (0.58, 1.46) | 1.14 (0.52, 1.46) | 1.10 (0.45, 1.45) | 1.08 (0.37, 1.48) | 1.07 (0.53, 1.37) | 1.16 (0.58, 1.46) | 1.37 (0.72, 1.83) |
|  | *E. coli* | 0.70 (0.40, 0.83) | 0.68 (0.39, 0.82) | 0.68 (0.39, 0.87) | 0.67 (0.34, 0.84) | 0.53 (0.31, 0.69) | 0.66 (0.40, 0.80) | 0.74 (0.43, 0.88) |
|  | *E. faecalis* | 0.45 (0.19, 0.66) | 0.42 (0.15, 0.62) | 0.38 (0.06, 0.59) | 0.43 (0.04, 0.67) | 0.40 (0.13, 0.61) | 0.45 (0.19, 0.66) | 0.59 (0.27, 0.87) |
|  | *E. faecium* | 0.01 (-0.08, 0.08) | 0.01 (-0.08, 0.07) | -0.01 (-0.10, 0.05) | -0.02 (-0.12, 0.04) | 0.01 (-0.07, 0.07) | 0.01 (-0.08, 0.08) | 0.02 (-0.07, 0.10) |
|  | *Enterococcus* spp. | 0.83 (-0.09, 1.33) | 0.75 (-0.30, 1.33) | 0.79 (-0.47, 1.51) | 0.98 (-0.71, 2.12) | 0.57 (-0.37, 1.15) | 0.83 (-0.09, 1.33) | 2.04 (0.03, 3.04) |
|  | *K. pneumoniae* | 0.12 (-0.20, 0.70) | 0.12 (-0.19, 0.73) | 0.08 (-0.25, 0.82) | 0.10 (-0.24, 0.81) | 0.48 (0.10, 0.78) | 0.55 (0.28, 0.76) | 0.10 (-0.25, 0.75) |
|  | *Morganella* spp. | 0.56 (0.17, 1.00) | 0.56 (0.09, 1.21) | 0.53 (-0.09, 1.30) | 0.23 (-0.47, 1.36) | 0.72 (0.27, 1.11) | 0.56 (0.17, 1.00) | 1.10 (0.37, 1.93) |
|  | *P. aeruginosa* | 0.63 (0.31, 0.81) | 0.63 (0.31, 0.81) | 0.62 (0.30, 0.81) | 0.59 (0.27, 0.78) | 0.58 (0.23, 0.77) | 0.63 (0.31, 0.81) | 0.68 (0.33, 0.87) |
|  | *S. agalactiae* | 0.32 (-0.10, 0.63) | 0.35 (-0.07, 0.68) | 0.33 (-0.03, 0.78) | 0.31 (-0.06, 0.91) | 0.21 (-0.05, 0.72) | 0.32 (-0.10, 0.63) | 2.23 (0.57, 2.91) |
|  | *S. aureus* | 0.36 (-0.01, 0.71) | 0.33 (-0.02, 0.68) | 0.29 (-0.07, 0.64) | 0.30 (-0.05, 0.67) | 0.37 (0.01, 0.75) | 0.36 (-0.01, 0.71) | 0.46 (0.04, 0.90) |
|  | *S. pneumoniae* | 0.28 (-0.06, 0.50) | 0.26 (-0.10, 0.49) | 0.22 (-0.21, 0.49) | 0.26 (-0.13, 0.52) | 0.27 (0.05, 0.48) | 0.28 (-0.06, 0.50) | 1.94 (0.31, 2.85) |
|  | *S. pyogenes* | 0.05 (-0.05, 0.15) | 0.04 (-0.07, 0.15) | 0.04 (-0.16, 0.19) | 0.08 (-0.29, 0.30) | 0.07 (-0.03, 0.21) | 0.05 (-0.05, 0.15) | 0.14 (-0.26, 0.51) |
|  | *Salmonella* spp. | 0.79 (-0.53, 1.42) | 0.83 (-0.70, 1.86) |  |  | 0.81 (-2.98, 6.86) | 0.63 (-0.61, 1.28) | 0.86 (-0.75, 1.58) |
| Sulfonamides and Trimethoprim | *Acinetobacter* spp. | 0.11 (-0.08, 0.31) |  |  |  | 0.16 (-0.22, 0.45) | 0.04 (-0.13, 0.17) | 0.18 (-0.13, 0.57) |
|  | *E. coli* | 0.00 (-0.36, 0.24) | 0.10 (-0.28, 0.35) |  |  | 0.00 (-0.47, 0.48) | -0.02 (-0.29, 0.20) | -0.01 (-0.48, 0.40) |
|  | *K. pneumoniae* | 0.09 (-0.30, 0.38) | 0.17 (-0.49, 0.74) |  |  | 0.12 (-0.34, 0.52) | 0.09 (-0.30, 0.38) | 0.02 (-0.63, 0.53) |
|  | *Morganella* spp. | -0.13 (-0.43, 0.35) |  |  |  | 0.15 (-0.57, 0.65) | -0.13 (-0.43, 0.35) | -0.17 (-0.80, 0.57) |
|  | *S. aureus* | -0.09 (-0.32, 0.48) | -0.14 (-0.37, 0.44) | -0.17 (-0.42, 0.48) |  | 0.22 (-0.22, 0.60) | -0.09 (-0.32, 0.48) | -0.14 (-0.58, 0.93) |
| Tetracyclines | *Acinetobacter* spp. | 0.10 (-0.24, 0.64) | 0.10 (-0.24, 0.68) | 0.05 (-0.32, 0.61) | -0.12 (-0.51, 0.48) | 0.12 (-0.24, 0.59) | 0.10 (-0.25, 0.50) | 0.18 (-0.38, 1.07) |
|  | *E. coli* | -0.11 (-0.23, 0.09) | -0.07 (-0.20, 0.11) | -0.08 (-0.20, 0.09) | -0.07 (-0.20, 0.10) | -0.07 (-0.24, 0.15) | -0.11 (-0.23, 0.09) | -0.09 (-0.42, 0.38) |
|  | *E. faecalis* | -0.05 (-0.22, 0.14) | -0.02 (-0.19, 0.16) | 0.03 (-0.13, 0.19) | 0.06 (-0.15, 0.20) | -0.04 (-0.28, 0.16) | -0.05 (-0.22, 0.14) | -0.09 (-0.42, 0.22) |
|  | *E. faecium* | 0.16 (-0.08, 0.38) | 0.17 (-0.11, 0.44) | 0.29 (0.03, 0.56) | 0.30 (0.04, 0.60) | 0.15 (-0.15, 0.43) | 0.16 (-0.08, 0.38) | 0.29 (-0.29, 0.82) |
|  | *Enterococcus* spp. | 0.06 (-0.33, 0.36) | -0.05 (-0.39, 0.33) | -0.30 (-0.72, 0.18) | -0.24 (-0.58, 0.89) | 0.02 (-0.50, 0.34) | 0.06 (-0.33, 0.36) | 0.34 (-0.69, 1.05) |
|  | *H. influenzae* | 0.02 (-0.05, 0.07) | 0.02 (-0.07, 0.07) | 0.02 (-0.08, 0.09) | 0.03 (-0.05, 0.10) | 0.02 (-0.03, 0.06) | 0.02 (-0.05, 0.07) | 0.44 (-0.19, 0.91) |
|  | *K. pneumoniae* | -0.09 (-0.22, 0.09) | -0.08 (-0.23, 0.11) | -0.09 (-0.24, 0.09) | -0.08 (-0.24, 0.10) | -0.07 (-0.24, 0.15) | -0.09 (-0.22, 0.09) | -0.19 (-0.60, 0.27) |
|  | *Morganella* spp. | 0.06 (-0.23, 0.41) | 0.03 (-0.26, 0.36) | -0.03 (-0.39, 0.28) | -0.09 (-0.52, 0.30) | 0.19 (-0.12, 0.52) | 0.06 (-0.23, 0.41) | 0.10 (-0.78, 1.18) |
|  | *S. agalactiae* | 0.05 (-0.10, 0.25) | 0.05 (-0.13, 0.25) | 0.02 (-0.21, 0.24) | 0.04 (-0.31, 0.30) | 0.08 (-0.11, 0.33) | 0.05 (-0.10, 0.25) | 0.06 (-0.28, 0.45) |
|  | *S. aureus* | 0.09 (-0.21, 0.35) | 0.09 (-0.23, 0.42) | 0.09 (-0.29, 0.48) | 0.03 (-0.35, 0.47) | 0.11 (-0.17, 0.35) | 0.09 (-0.21, 0.35) | 0.31 (-0.44, 0.91) |
|  | *S. pyogenes* | -0.02 (-0.14, 0.15) | -0.02 (-0.10, 0.10) | -0.01 (-0.07, 0.09) | 0.00 (-0.07, 0.05) | 0.02 (-0.13, 0.16) | -0.02 (-0.14, 0.15) | -0.02 (-0.60, 0.76) |

Lagged analyses match data from 1, 3, or 5 years previous with the corresponding AMR data. Some antibiotic class-pathogen combinations do not have data spanning these lengths of time, these appear as blank cells in the table. The extra variables analysis uses the first ten principal components of the World Bank Health, Nutrition, and Population Statistics variables rather than the first three. The removed influential observations analysis is run excluding observations with Cook’s distance above 1. The lower continuity analysis uses a continuity correction of 0.01 instead of 1 for the log-transform of the AMR percent prevalence.
