# Supplementary material for: Burden of bacterial antimicrobial resistance associated with modifiable human antibiotic consumption: An observational study
Source: PLoS Med. 2026 Oct 26;23(10):e1005140. doi: 10.1371/journal.pmed.1005140 (PMC13719055; doi:10.1371/journal.pmed.1005140)
Supplement: Supplementary file 42 [file pmed.1005140.s019.docx]

**Table S17: Comparison of calculated elasticities with log-odds coefficients in binomial and beta-binomial models.**

| **Antibiotic class** | **Pathogen** | **Elasticity (95% CI)** | **Binomial coefficient, log odds scale (95% CI)** | **Beta-binomial coefficient, log odds scale (95% CI)** | **Beta-binomial dispersion** |
| --- | --- | --- | --- | --- | --- |
| Tetracyclines | *Acinetobacter* spp. | 0.10 (-0.24, 0.64) | -0.05 (-0.10, -0.01) | 0.03 (-0.19, 0.34) | 2.99 |
|  | *E. coli* | -0.11 (-0.23, 0.09) | -0.21 (-0.24, -0.17) | -0.08 (-0.27, 0.08) | 6.69 |
|  | *E. faecalis* | -0.05 (-0.22, 0.14) | -0.03 (-0.07, 0.01) | 0.02 (-0.13, 0.15) | 2.91 |
|  | *E. faecium* | 0.16 (-0.08, 0.38) | 0.16 (0.08, 0.24) | 0.10 (-0.06, 0.30) | 6.65 |
|  | *Enterococcus* spp. | 0.06 (-0.33, 0.36) | -0.08 (-0.33, 0.16) | -0.08 (-0.50, 0.32) | 6.41 |
|  | *H. influenzae* | 0.02 (-0.05, 0.07) | 0.18 (-0.12, 0.47) | 0.19 (-0.18, 0.58) | 47.68 |
|  | *K. pneumoniae* | -0.09 (-0.22, 0.09) | -0.10 (-0.13, -0.06) | -0.07 (-0.21, 0.08) | 6.23 |
|  | *Morganella* spp. | 0.06 (-0.23, 0.41) | 0.20 (-0.13, 0.52) | 0.19 (-0.26, 0.72) | 36.62 |
|  | *S. agalactiae* | 0.05 (-0.10, 0.25) | 0.12 (0.08, 0.16) | 0.17 (-0.02, 0.46) | 2.93 |
|  | *S. aureus* | 0.09 (-0.21, 0.35) | 0.19 (0.13, 0.25) | 0.05 (-0.26, 0.43) | 11.46 |
|  | *S. pyogenes* | -0.02 (-0.14, 0.15) | 0.54 (-0.28, 1.35) | 0.19 (-8.44, 5.60) | 55.23 |
| Penicillins | *Acinetobacter* spp. | 0.32 (0.02, 0.61) | 0.46 (0.41, 0.52) | 0.57 (0.12, 1.00) | 2.94 |
|  | *E. coli* | 0.02 (-0.04, 0.09) | 0.02 (-0.01, 0.06) | 0.05 (-0.15, 0.25) | 20.28 |
|  | *E. faecalis* | -0.03 (-0.28, 0.22) | -0.09 (-0.27, 0.09) | -0.09 (-0.45, 0.41) | 8.71 |
|  | *E. faecium* | 0.03 (-0.02, 0.11) | -0.07 (-0.19, 0.04) | -0.02 (-0.30, 0.29) | 10.36 |
|  | *Enterococcus* spp. | -0.19 (-0.80, 0.65) | -0.31 (-0.59, -0.03) | -0.12 (-0.64, 0.65) | 2.20 |
|  | *H. influenzae* | 0.21 (-0.14, 0.48) | 0.10 (0.03, 0.17) | 0.04 (-0.23, 0.34) | 10.18 |
|  | *K. pneumoniae* | 0.01 (-0.15, 0.28) | -0.22 (-0.25, -0.19) | 0.13 (-0.04, 0.33) | 2.29 |
|  | *Morganella* spp. | 0.00 (-0.00, 0.00) | 0.01 (-∞, ∞) | 1.76 (0.67, 6.95) | 5537689.35 |
|  | *P. aeruginosa* | 0.04 (-0.23, 0.30) | 0.00 (-0.03, 0.03) | -0.02 (-0.21, 0.18) | 18.70 |
|  | *S. aureus* | 0.75 (0.16, 1.58) | 0.64 (0.62, 0.65) | 0.48 (-0.02, 1.04) | 4.31 |
|  | *S. pneumoniae* | 0.32 (-0.31, 0.93) | 0.58 (0.54, 0.62) | 0.28 (-0.07, 0.71) | 5.89 |
| Non-Penicillin Beta-Lactams | *Acinetobacter* spp. | 0.30 (-0.31, 0.73) | 0.37 (0.35, 0.39) | 0.38 (-0.05, 0.66) | 1.27 |
|  | *E. coli* | 0.28 (0.08, 0.38) | 0.35 (0.35, 0.35) | 0.11 (-0.05, 0.22) | 2.15 |
|  | *K. pneumoniae* | 0.10 (-0.18, 0.55) | 0.15 (0.14, 0.15) | 0.20 (-0.08, 0.41) | 1.65 |
|  | *Morganella* spp. | 0.02 (-0.05, 0.07) | 0.10 (-0.03, 0.23) | 0.10 (-0.10, 0.22) | 252.29 |
|  | *P. aeruginosa* | 0.35 (0.13, 0.55) | 0.37 (0.36, 0.38) | 0.18 (-0.04, 0.35) | 5.14 |
|  | *S. aureus* | -0.01 (-0.07, 0.02) | -1.72 (-2.77, -0.68) | -1.05 (-3.98, 25.10) | 66.94 |
|  | *S. pneumoniae* | 0.19 (-0.13, 0.61) | 0.39 (0.32, 0.45) | 0.44 (0.22, 0.73) | 6.14 |
|  | *Salmonella* spp. | -0.21 (-0.67, 0.42) | -0.19 (-0.30, -0.07) | -0.11 (-0.53, 0.88) | 16.94 |
| Sulfonamides and Trimethoprim | *Acinetobacter* spp. | 0.11 (-0.08, 0.31) | 0.31 (0.20, 0.42) | 0.20 (-0.07, 0.50) | 5.11 |
|  | *E. coli* | 0.00 (-0.36, 0.24) | 0.03 (0.02, 0.04) | -0.04 (-0.20, 0.14) | 22.69 |
|  | *K. pneumoniae* | 0.09 (-0.30, 0.38) | 0.23 (0.22, 0.25) | 0.09 (-0.14, 0.28) | 14.43 |
|  | *Morganella* spp. | -0.13 (-0.43, 0.35) | -0.25 (-0.43, -0.06) | -0.13 (-0.47, 0.35) | 12.64 |
|  | *S. aureus* | -0.09 (-0.32, 0.48) | -0.09 (-0.15, -0.04) | 0.08 (-0.39, 0.71) | 40.54 |
| Macrolides | *E. faecalis* | 0.09 (0.01, 0.26) | 0.23 (0.05, 0.41) | 0.25 (-0.07, 0.76) | 6.10 |
|  | *E. faecium* | 0.04 (-0.06, 0.11) | 0.23 (-0.01, 0.47) | 0.24 (-0.37, 0.58) | 40.68 |
|  | *S. agalactiae* | -0.20 (-0.82, 0.61) | -0.14 (-0.33, 0.05) | -0.19 (-0.92, 0.54) | 5.43 |
|  | *S. aureus* | 0.20 (-0.17, 0.57) | 0.25 (0.20, 0.29) | 0.42 (-0.14, 0.98) | 7.03 |
|  | *S. pneumoniae* | 0.68 (-0.03, 1.27) | 1.05 (1.02, 1.07) | 0.75 (0.27, 1.31) | 2.77 |
|  | *S. pyogenes* | -0.26 (-1.03, 0.64) | -0.28 (-0.46, -0.10) | -0.47 (-1.22, 0.47) | 4.05 |
| Aminoglycosides | *Acinetobacter* spp. | 0.15 (-0.26, 0.52) | 0.31 (0.29, 0.33) | 0.18 (-0.10, 0.39) | 2.86 |
|  | *E. coli* | 0.21 (-0.01, 0.32) | 0.21 (0.20, 0.22) | 0.13 (-0.02, 0.28) | 24.13 |
|  | *E. faecalis* | 0.08 (-0.33, 0.44) | 0.13 (0.10, 0.15) | 0.19 (-0.16, 0.50) | 19.51 |
|  | *E. faecium* | 0.31 (-0.17, 0.69) | 0.41 (0.37, 0.44) | 0.32 (-0.23, 0.92) | 9.03 |
|  | *K. pneumoniae* | 0.43 (0.12, 0.72) | 0.44 (0.42, 0.46) | 0.32 (0.16, 0.52) | 5.49 |
|  | *Morganella* spp. | 0.28 (-0.04, 0.55) | 0.20 (-0.01, 0.42) | 0.31 (-0.08, 0.69) | 12.62 |
|  | *P. aeruginosa* | 0.22 (-0.09, 0.56) | 0.36 (0.34, 0.39) | 0.21 (0.02, 0.39) | 11.19 |
|  | *S. aureus* | 0.09 (-0.34, 0.46) | 0.07 (0.04, 0.11) | 0.11 (-0.38, 0.42) | 9.76 |
| Quinolones | *Acinetobacter* spp. | 1.16 (0.58, 1.46) | 2.00 (1.95, 2.04) | 0.82 (0.43, 1.24) | 3.14 |
|  | *E. coli* | 0.70 (0.40, 0.83) | 0.98 (0.97, 0.98) | 0.54 (0.33, 0.76) | 13.30 |
|  | *E. faecalis* | 0.45 (0.19, 0.66) | 0.45 (0.38, 0.51) | 0.35 (0.15, 0.60) | 13.51 |
|  | *E. faecium* | 0.01 (-0.08, 0.08) | -0.02 (-0.13, 0.08) | -0.14 (-0.44, 0.18) | 15.26 |
|  | *Enterococcus* spp. | 0.83 (-0.09, 1.33) | 0.30 (-0.03, 0.63) | 0.28 (-0.31, 0.86) | 7.18 |
|  | *K. pneumoniae* | 0.12 (-0.20, 0.70) | 0.43 (0.41, 0.44) | 0.51 (0.22, 0.79) | 8.24 |
|  | *Morganella* spp. | 0.56 (0.17, 1.00) | 0.36 (0.18, 0.54) | 0.45 (0.13, 0.86) | 9.82 |
|  | *P. aeruginosa* | 0.63 (0.31, 0.81) | 0.69 (0.67, 0.71) | 0.52 (0.26, 0.74) | 15.02 |
|  | *S. agalactiae* | 0.32 (-0.10, 0.63) | -0.20 (-0.40, 0.01) | 0.68 (-0.46, 1.44) | 7.32 |
|  | *S. aureus* | 0.36 (-0.01, 0.71) | 0.31 (0.29, 0.33) | 0.48 (0.09, 0.87) | 4.98 |
|  | *S. pneumoniae* | 0.28 (-0.06, 0.50) | 0.17 (-0.07, 0.41) | 0.98 (0.06, 1.82) | 34.77 |
|  | *S. pyogenes* | 0.05 (-0.05, 0.15) | 0.19 (-1.98, 2.36) | -0.30 (-3.19, 4.17) | 63.57 |
