# Supplementary material for: Burden of bacterial antimicrobial resistance associated with modifiable human antibiotic consumption: An observational study
Source: PLoS Med. 2026 Oct 26;23(10):e1005140. doi: 10.1371/journal.pmed.1005140 (PMC13719055; doi:10.1371/journal.pmed.1005140)
Supplement: Supplementary file 43 [file pmed.1005140.s020.docx]

**S18 Table: Difference in AMR burden estimated using elasticities calculated under different sensitivity analyses.**

| **Region** | **1y lag** | **3y lag** | **5y lag** | **Extra PCA variables** | **Removed influential** | **Lower continuity** | **Half elasticity** | **Double elasticity** |
| --- | --- | --- | --- | --- | --- | --- | --- | --- |
| Andean Latin America | 351 (269, 442) | 353 (270, 444) | 358 (274, 457) | 383 (299, 473) | 366 (281, 456) | 472 (369, 584) | 183 (141, 232) | 691 (530, 861) |
| Australasia | 51 (36, 68) | 52 (43, 61) | 55 (39, 73) | 59 (45, 76) | 53 (39, 70) | 68 (51, 90) | 27 (20, 36) | 102 (75, 134) |
| Caribbean | 255 (208, 307) | 251 (206, 285) | 262 (209, 313) | 277 (232, 328) | 268 (223, 316) | 372 (313, 431) | 133 (109, 158) | 503 (411, 593) |
| Central Asia | 339 (287, 394) | 338 (315, 377) | 340 (282, 402) | 364 (313, 420) | 351 (301, 403) | 482 (414, 552) | 176 (149, 206) | 666 (564, 773) |
| Central Europe | 585 (477, 718) | 574 (495, 639) | 594 (473, 735) | 624 (527, 743) | 607 (502, 731) | 783 (648, 939) | 304 (248, 371) | 1151 (943, 1387) |
| Central Latin America | 1107 (877, 1344) | 1089 (838, 1298) | 1138 (919, 1397) | 1186 (978, 1420) | 1158 (940, 1412) | 1508 (1236, 1802) | 582 (468, 710) | 2195 (1784, 2647) |
| Central Sub-Saharan Africa | 607 (516, 697) | 608 (538, 693) | 641 (552, 736) | 692 (607, 783) | 636 (553, 722) | 1165 (1007, 1333) | 316 (274, 362) | 1199 (1040, 1366) |
| East Asia | 5134 (3508, 6860) | 4918 (3038, 6805) | 5410 (3772, 7174) | 5943 (4547, 7648) | 5581 (4249, 7144) | 7162 (5206, 9137) | 2763 (2024, 3593) | 10379 (7599, 13372) |
| Eastern Europe | 1089 (912, 1288) | 1078 (997, 1158) | 1099 (913, 1315) | 1130 (950, 1333) | 1087 (909, 1275) | 1409 (1170, 1645) | 560 (466, 657) | 2126 (1778, 2480) |
| Eastern Sub-Saharan Africa | 1813 (1569, 2098) | 1767 (1625, 2093) | 1908 (1676, 2148) | 2133 (1898, 2375) | 1901 (1657, 2173) | 3191 (2787, 3604) | 946 (818, 1082) | 3592 (3113, 4095) |
| High-income Asia Pacific | 1289 (838, 1775) | 1154 (630, 1440) | 1345 (930, 1866) | 1451 (991, 1943) | 1333 (904, 1838) | 1746 (1220, 2371) | 679 (456, 937) | 2547 (1735, 3454) |
| High-income North America | 1581 (1145, 2131) | 1600 (1323, 1739) | 1679 (1209, 2194) | 1793 (1350, 2341) | 1655 (1231, 2215) | 2068 (1536, 2764) | 837 (615, 1129) | 3143 (2336, 4169) |
| North Africa and Middle East | 2275 (1872, 2723) | 2379 (1911, 2635) | 2355 (1909, 2817) | 2505 (2111, 2952) | 2331 (1891, 2791) | 3251 (2681, 3842) | 1186 (954, 1438) | 4469 (3618, 5359) |
| Oceania | 61 (52, 71) | 60 (56, 66) | 66 (56, 76) | 68 (59, 77) | 64 (55, 74) | 97 (85, 112) | 32 (27, 37) | 121 (104, 140) |
| South Asia | 11532 (9960, 13248) | 11069 (9595, 12155) | 11693 (10011, 13524) | 12351 (10511, 14302) | 11397 (9828, 13074) | 16904 (14858, 19172) | 6009 (5229, 6918) | 22765 (19799, 26116) |
| Southeast Asia | 2968 (2467, 3506) | 2672 (2382, 2957) | 3005 (2425, 3634) | 3209 (2710, 3762) | 3058 (2550, 3588) | 4100 (3432, 4806) | 1544 (1274, 1828) | 5827 (4834, 6883) |
| Southern Latin America | 465 (336, 616) | 471 (392, 572) | 475 (355, 620) | 507 (377, 655) | 478 (356, 611) | 623 (466, 788) | 239 (177, 308) | 901 (674, 1140) |
| Southern Sub-Saharan Africa | 351 (280, 424) | 339 (301, 369) | 359 (296, 430) | 399 (336, 461) | 370 (306, 436) | 518 (430, 610) | 184 (150, 218) | 693 (569, 814) |
| Tropical Latin America | 1122 (897, 1397) | 1084 (912, 1424) | 1143 (889, 1448) | 1229 (993, 1476) | 1161 (933, 1417) | 1499 (1201, 1814) | 583 (461, 716) | 2199 (1754, 2667) |
| Western Europe | 1412 (1118, 1770) | 1589 (1229, 2203) | 1425 (1097, 1779) | 1518 (1203, 1876) | 1443 (1151, 1805) | 1904 (1541, 2336) | 731 (580, 920) | 2764 (2211, 3427) |
| Western Sub-Saharan Africa | 3026 (2616, 3429) | 3158 (2924, 3440) | 3161 (2781, 3536) | 3449 (3050, 3813) | 3147 (2777, 3525) | 5136 (4515, 5789) | 1566 (1371, 1770) | 5947 (5215, 6700) |
| Total | 37413 (34811, 40158) | 36679 (34183, 39334) | 38512 (35769, 41765) | 41270 (38515, 44047) | 38446 (35945, 41030) | 54457 (51116, 57813) | 19583 (18248, 20962) | 73980 (69074, 79030) |

Lagged analyses match consumption data from 1, 3, or 5 years previous with the corresponding AMR data. Some antibiotic class-pathogen combinations do not have data spanning these lengths of time, these appear as blank cells in the table. The extra variables analysis uses the first ten principal components of the World Bank Health, Nutrition, and Population Statistics variables rather than the first three. The removed influential analysis excludes observations with Cook’s distance measures greater than 1. The lower continuity correction analysis uses a continuity correction of 0.01% instead of 1% for the log-transform of the AMR percent prevalence. The half and double elasticity scenarios assume that elasticities are half or double across all antibiotic class-pathogen combinations, respectively (equivalent to dividing $\epsilon$ estimates from the primary analyses presented in **Figure 1** by 2 and multiplying $\epsilon$ by 2, respectively). We omit an additional sensitivity analysis presented in **S16 Table**, which showed elasticity coefficient values obtained in regression models scaling antibiotic use by values in 2000 (instead of 2018), because there were no changes in coefficient estimates to the reported level of precision.
